# Pyroptosis‐Inducing Platinum(IV) Prodrugs via GSDME Pathway for Chemoimmunotherapy and Metastasis Inhibition in Triple‐Negative Breast Cancer

**DOI:** 10.1002/advs.202505567

**Published:** 2025-05-28

**Authors:** Xinda Yang, Chuansheng Xu, Youliang Zeng, Chunhui Wang, Yan Gao, Jie Ding, Sirui Chen, Yuheng Pan, Xin Zhang, Zongwan Mao, Shuo Shi

**Affiliations:** ^1^ School of Chemical Science and Engineering Department of Laboratory Medicine Shanghai Tenth People's Hospital of Tongji University Tongji University Shanghai 200092 P. R. China; ^2^ MOE Key Laboratory of Bioinorganic and Synthetic Chemistry Guangdong Basic Research Center of Excellence for Functional Molecular Engineering GBRCE for Functional Molecular Engineering School of Chemistry Sun Yat‐Sen University Guangzhou 510275 P. R. China

**Keywords:** caspase‐3 cleaving GSDME, chemoimmunotherapy, metastasis inhibition, prodrugs, pyroptosis

## Abstract

Pyroptosis has attracted significant attention for its role in cancer chemotherapy and immunotherapy. However, few drugs have been reported to induce pyroptosis via the Caspase‐3/gasdermin E (GSDME) pathway. Herein, three novel Pt^IV^ prodrugs, **MRP**, **DRP**, and **HRP** are rationally designed by conjugating DNA methyltransferase (DNMT) inhibitor (RG108) and/or histone deacetylase (HDAC) inhibitor (PhB) to the Pt^IV^ center. These prodrugs can be easily reduced to cisplatin (**CDDP**) due to the high glutathione (GSH) levels in tumors, liberating the coordinated ligands. Released RG108 reactivates the GSDME gene and reduces pyroptosis in low GSDME‐expressing tumor cells. Meanwhile, PhB‐induced chromatin loosening enhances CDDP‐DNA binding, which not only increases Caspase‐3 expression, but also upregulates GSDME. **HRP** demonstrates superior ability to suppress tumor growth and metastasis while reducing systemic toxicity compared with **CDDP**. By reactivating GSDME and loosening chromatin, **HRP** effectively boosts tumor cell pyroptosis and exhibits the most pronounced anticancer performance. These findings highlight **HRP**’s potential as a therapeutic agent for triple‐negative breast cancer (TNBC) and offer innovative strategies for combining chemotherapy with immunotherapy. To the best of current knowledge, this is the first report of platinum complexes inducing pyroptosis via the Caspase‐3/GSDME pathway in low GSDME‐expressing tumor cells.

## Graphical abstract

**Three Pt^IV^ prodrugs** MRP, DRP, and HRP are designed to induce pyroptosis in low GSDME‐expressing tumor cells via the Caspase‐3/GSDME pathway. By relaxing chromatin and reactivating GSDME, HRP can effectively induce pyroptosis, enhance pro‐inflammatory cytokine production, and exhibit potent antitumor effects. This strategy provides a new approach for immunotherapy targeting low GSDME‐expressing tumors, offering potential for improved treatment outcomes.

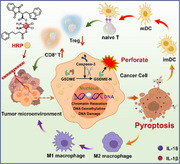

## 1 Introduction

Triple‐negative breast cancer (TNBC) is a highly aggressive and worrying subtype of breast cancer identified by the lack of estrogen receptor (ER), progesterone receptor (PR), and human epidermal growth factor receptor 2 (HER2).^[^ ^1^ ^]^ These receptor deficit causes TNBC to be unresponsive to traditional endocrine treatment and HER2‐targeted therapy, causing poor prognosis and significant recurrence rates.^[^ ^2^ ^]^ Due to its characteristics of high invasiveness, rapid proliferation, enhanced angiogenesis, and effective immune escape mechanisms, TNBC cells can rapidly invade peripheral tissues and blood vessels, penetrate the basement membrane, and spread to the vasculature and the lymphatic system, resulting in metastasis in vital organs such as the liver, kidneys, and lungs.^[^ ^3^ ^]^ Therefore, it is highly demanded to develop fresh therapeutic strategies for increasing the clinical outcomes of TNBC sufferers.^[^ ^4^ ^]^

Among several therapeutic options, chemotherapy remains the mainstay in the treatment of malignant tumors.^[^ ^5^ ^]^ It may deliver rapid benefits and successfully manage hard‐to‐treat tumors, greatly raising the cure rate.^[^ ^6^ ^]^ However, chemotherapy typically encounters challenges such as drug resistance and side effects, which not only diminish its long‐term impact but also may cause patients to suffer from poor quality of life.^[^ ^7^ ^]^ Recently, immunotherapy, which can successfully motivate the autoimmune system has drawn great interest because of its high specificity and long‐lasting effectiveness.^[^ ^8^ ^]^ However, the effects of immunotherapy depend on the individual patient, which may cause severe immune‐related adverse effects and usually takes a long time for the therapeutic effects to be realized.^[^ ^9^ ^]^ Combining chemotherapy with immunotherapy takes advantage of the rapid anti‐tumor effects of chemotherapy and the long‐term immune response of immunotherapy for cancer treatment,^[^ ^10^ ^]^ which may significantly enhance the efficiency and prolong patients' survival.^[^ ^11^ ^]^ Therefore, discovering new chemotherapeutic drugs with immunomodulatory properties has become an urgent focus in modern oncology research.^[^ ^12^ ^]^

Recently, it has been reported that some chemotherapeutic drugs capable of activating Caspase‐3 could also enhance immune against malignancies by inducing pyroptosis in certain tumor cells.^[^ ^13^ ^]^ Pyroptosis is a newly discovered form of programmed cell death characterized by gradual cell swelling followed by cell membrane rupture due to pores formed by cleaved gasdermin proteins.^[^ ^14^ ^]^ The rupture of the cell membrane leads to the rapid release of intracellular contents, including mature pro‐inflammatory cytokines (e.g., interleukin IL‐1β and IL‐18), which trigger a strong inflammatory response. This inflammatory response further stimulates antigen‐presenting cells, promotes the activation of specific T cells, and ultimately leads to a strong immune response.^[^ ^15^ ^]^ Different from pyroptosis classically caused by the cleavage of GSDMD (gasdermin D) via Caspases‐1/11/4/5,^[^ ^16^ ^]^ recent studies have found that Caspase‐3, which is usually associated with apoptosis, can also induce pyroptosis by cleaving the tumor suppressor gene gasdermin E (GSDME).^[^ ^17^ ^]^ GSDME, also known as DFNA5 (Deafness, Autosomal Dominant 5), is a member of the gasdermin family and plays an important role in executing the process of focal death. Unfortunately, the GSDME gene is frequently hypermethylated in the promoter region in many cancer cell lines and primary tumors, significantly reducing its expression. Low expression of GSDME resulted in these cells becoming resistant to Caspase‐3‐induced pyroptosis therapy activated by many specific chemotherapeutic drugs. Thus, how to reactivate the GSDME gene becomes the primary task. Fortunately, N‐phthaloyl‐L‐tryptophan (RG108), a non‐nucleotide DNA methyltransferase (DNMT) inhibitor was discovered to disrupt DNA methylation and revive suppressed genes specifically.^[^ ^18^ ^]^ Unlike conventional nucleotide DNMT inhibitors such as 5‐azacytidine and 5‐azodeoxycytidine, RG108 binds directly to the catalytic domain of DNMT, inhibiting the methylation step without embedding in the DNA molecule, thus lowering possible toxicity and side effects.^[^ ^19^ ^]^ Notably, RG108 molecule include carboxyl groups, which facilitates chemical structure modification. Therefore, the synergistic design of RG108 in conjunction with certain medicines might lead to the creation of new chemotherapeutic drugs that can promote tumor cell pyroptosis, exhibit immunotherapeutic qualities, and drastically lessen toxicity side effects.

Cisplatin (**CDDP**) is a commonly used platinum‐based chemotherapeutic drugs for effectively treating many malignancies.^[^ ^20^ ^]^ However, acquired and intrinsic resistance often limit its clinical utility as a first‐line treatment, thus, **CDDP** is usually taken alongside other drugs to boost efficacy.^[^ ^21^ ^]^ Pt^IV^ complex, which is considered to be a prodrug, is obtained by oxidizing cisplatin with hydrogen peroxide. It is inert in the plasma but can be reduced to Pt^II^ complex in the high glutathione (GSH) environment of tumors.^[^ ^22^ ^]^ It should be noted that the reducibility of Pt^IV^ prodrugs was not universal, as it largely depended on their specific ligand composition and redox potential. These reducibility qualities dramatically lowered the harmful side effects of Pt^II^ drugs. Additionally, the axial hydroxyl groups of Pt^IV^ prodrugs afford chances for molecular alterations, leading to the creation of novel chemotherapeutic drugs with varied treatment modalities. As a histone deacetylase (HDAC) inhibitor, phenylbutyric acid (PhB) can effectively effectively inhibit the activity of HDAC, leading to the loosening of chromatin structure that makes DNA more easily exposed.^[^ ^23^ ^]^ The exposure of DNA not only makes **CDDP** more easily enter chromatin and enhances interaction with DNA but also provides favorable conditions for the transcriptional regulation of start genes.^[^ ^24^ ^]^

**Scheme 1:**
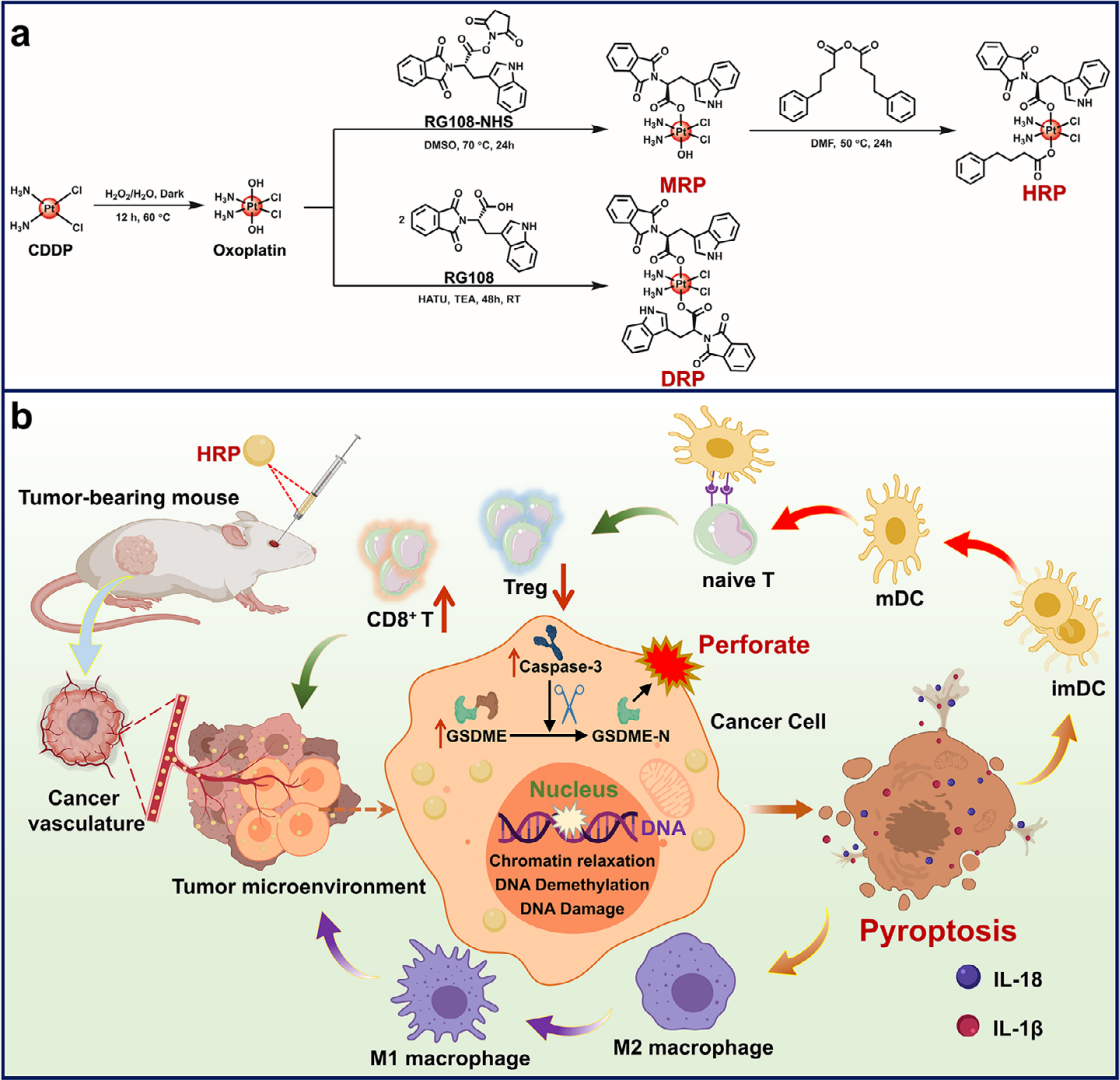
Synthetic routes and mechanism diagram. a) Synthetic routes of **CDDP**, **MRP**, **DRP**, and **HRP**; b) Mechanism diagram of **HRP**‐induced pyroptosis in 4T1 cells and triggering tumor immunotherapy.

While in our previous work, we reported a platinum complex that induces pyroptosis in melanoma cells with high GSDME expression through Caspase‐3 cleavage of GSDME protein,^[^ ^25^ ^]^ no innovative platinum‐based drugs have yet been observed to produce similar effects in tumor cells with low GSDME expression. In this work, three Pt^IV^ prodrugs were designed to induce cell pyroptosis via the Caspase‐3 cleaved GSDME protein, named **MRP**, **DRP**, and **HRP** (**Scheme** **1**a). **MRP** and **DRP** were synthesized by coupling one or two RG108 molecules to the Pt^IV^ center, respectively. As a DNMT inhibitor, RG108 can effectively inhibit DNA methylation and reactivate the GSDME gene, which can be cleaved by Caspase‐3 induced by **CDDP**, then triggers the pyroptosis in tumor cells and produces immunotherapeutic effects. What is more, to address the insensitivity of single‐agent **CDDP** in inducing apoptosis in tumor cells, the remaining hydroxyl group on **MRP** was coupled with PhB to prepare an axially asymmetric molecule **HRP**, in the hope that the chromatin loosening induced by PhB would make **CDDP** more readily available for binding to DNA, enhance its pro‐apoptotic effect, and increase the expression of Caspase‐3. At the same time, the DNA exposed by chromatin relaxation facilitates transcription initiation and gene expression regulation, enhancing RG108's ability to upregulate GSDME expression by inhibiting DNA methylation, further promoting GSDME protein production (Scheme 1b). It is expected that this design could increase the cleavage of GSDME by Caspase‐3, thereby enhancing the induction of pyroptosis and activating a stronger cancer immune effect.

## 2 Results and Discussion

### 2.1 Synthesis and Characterization

**MRP** and **DRP** were synthesized by linking one or two RG108 moieties as axial ligands to **CDDP**, followed by the reaction of **MRP** with 4‐phenylbutyric acid anhydride to obtain the asymmetric molecule **HRP**. Briefly, **CDDP** was oxidized with hydrogen peroxide to obtain oxoplatin, which was then coupled with RG108 on both axial positions in the presence of 2‐(7‐Azabenzotriazol‐1‐yl)‐N, N, N’, N’‐tetramethyluronium hexafluorophosphate and triethylamine to obtain the target complex **DRP**. **MRP** was prepared by activating RG108 with N‐Hydroxy succinimide to obtain RG108‐NHS, which was then reacted with oxoplatin. **HRP** was prepared by reacting **MRP** with 4‐phenylbutyric acid anhydride. The synthetic routes are summarized in Scheme 1a.

**MRP**, **DRP**, and **HRP** were fully characterized by ^1^H‐, ^13^C‐, ^195^Pt NMR, and HR‐ESI‐MS (Figures S1–S18, Supporting Information). The major peaks at m/z 650.0526 and 672.0423 in the HR‐ESI‐MS spectrum assigned to [M + H]^+^ and [M + Na]^+^ of **MRP**, respectively. The peak at m/z 989.1204 assigned to [M + Na]^+^ of **DRP**, while the peaks at m/z 797.12609 and 819.11258 assigned to [M + H]^+^ and [M + Na]^+^ of **HRP**, respectively. The presence of Pt^IV^ in **MRP**, **DRP**, and **HRP** was confirmed by the ^195^Pt NMR signals at ≈δ 1043.49, 1218.02, and 1117.86 ppm, respectively. The ammonia ligands in the complexes were indicated by broad peaks, ranging from δ 5.65 to 6.75 in the ^1^H NMR spectra. All HR‐ESI‐MS and NMR data demonstrated the successful synthesis of **MRP**, **DRP**, and **HRP**. Three Pt^IV^ prodrugs can be soluble in a range of organic solvents such as methanol, dimethylformamide (DMF), and dimethyl sulfoxide (DMSO). The lipophilicity of the synthesized complexes was evaluated by measuring their octanol‐water partition coefficients (log*P*_O/W_). The log*P*_O/W_ values for **MRP**, **DRP**, and **HRP** were identified as 0.95, 1.65, and 1.76, respectively (Table S1, Supporting Information). These data suggested much greater lipophilicity than **CDDP**, which had a log*P*_O/W_ of −2.33. Generally, increased lipophilicity is related to improved passive diffusion across cellular membranes. Thus, **MRP**, **DRP**, and **HRP** are likely to penetrate cell membranes more readily than **CDDP**, possibly leading to higher cellular accumulation. Conjunction of RG108 and PhB significantly increases the lipophilicity of these complexes indicating higher cellular absorption and increased biological activity.

### 2.2 Stability and Reducibility

Typically, the anticancer effect of Pt^IV^ cisplatin prodrugs stems from their reduction by reducing agents such as ascorbic acid (ASA) or GSH, generating Pt^II^ species that interact with DNA. In order to explore the changes in **MRP**, **DRP**, and **HRP** under reducing conditions, further in vitro studies were carried out. Pt^IV^ prodrugs were incubated with or without the cytoreductive agent ASA, and the proportion of intact Pt^IV^ prodrugs remaining was determined using reversed‐phase high‐performance liquid chromatography (RP‐HPLC) and isocratic elution. The data showed that **MRP**, **DRP**, and **HRP** exhibited excellent stability over 72 h, retaining ≈83.33%, 80.56%, and 91.22% of the Pt^IV^ content, respectively (Figures S19–S22, Supporting Information). In the presence of ASA, all three complexes exhibited rapid reductive degradation, with the Pt^IV^ content completely reduced to 0% within 48 h, while **MRP** even disappeared within 24 h (Figures S19–S22, Supporting Information). The intracellular stability of **MRP**, **DRP**, and **HRP** was evaluated by RP‐HPLC after incubation with 4T1 cells for different periods (6, 24, and 48 h). As shown in Figures S23–S25 (Supporting Information), the characteristic peaks of the complexes were still detectable after 6 h of incubation. With prolonged incubation, the peaks corresponding to the intact complexes gradually decreased, and almost complete degradation was observed at 48 h. Notably, **MRP** underwent complete degradation within 24 h. These results indicated that the three Pt^IV^ prodrugs could be effectively reduced and degraded under the intracellular reductive environment, consistent with the findings from extracellular simulation experiments. Subsequently, ¹⁹⁵Pt NMR was used to monitor the reduction of Pt^IV^ complexes incubated with AsA (Figures S26–S28, Supporting Information). The individual complexes **MRP**, **DRP**, and **HRP** exhibited characteristic Pt^IV^ NMR signals. After incubation, the Pt^IV^ signal of **MRP** disappeared within 24 h, while the Pt^IV^ signals of **DRP** and **HRP** completely disappeared within 72 h, accompanied by the appearance of corresponding Pt^II^ signals. These results were consistent with HPLC data, further confirming that all three prodrugs could be reduced and degraded. In addition, the reaction of 1 mm of **MRP**, **DRP**, and **HRP** with 4 mm of 5′‐GMP in an aqueous solution containing 10 mm of ASA for 96 h was also verified. The ESI‐MS spectra of the reaction mixtures showed new signals at m/z 922.08 and 956.14, which could be related to the generation of cis‐Pt(NH_3_)_2_(5′‐GMP)_2_ and Pt(5′‐GMP)_2_ adducts, suggesting that **CDDP** was released from **MRP**, **DRP**, and **HRP** and further bound to DNA (Figures S29–S31, Supporting Information). These findings confirmed that these prodrugs are highly stable in the absence of reducing agents and can bind to DNA by rapidly reducing and releasing active Pt^II^ molecules under reducing conditions. This reduction‐activation process suggests that three Pt^IV^ prodrugs have the potential to remain stable in circulation and to be selectively activated at the cancer site, thus increasing their tumor‐specific therapeutic efficiency.

### 2.3 In Vitro Cytotoxicity

Cytotoxicity of **MRP**, **DRP**, and **HRP** was assessed using the CCK8 assay. The cell lines tested included several tumor cell lines with low GSDME expression: three human breast cancer cell lines: MDA‐MB‐231 (ER/PR‐negative, HER2‐negative, TNBC), BT‐474 (ER‐positive, HER2‐positive, PR negative), and MCF‐7 (ER‐positive, PR‐positive, HER2‐negative), the human bladder cancer cell line T24, the human prostate cancer cell DU145, the mouse mammary carcinoma cell line 4T1. One high GSDME‐expressing tumor cell line, the human lung cancer cell line A549 was included. Saline, **CDDP**, and RG108 were used for comparison. As presented in **Table** **1**, **MRP**, **DRP**, and **HRP** demonstrated much greater cytotoxicity than **CDDP** in drugs. The axial disubstituted products **HRP** and **DRP** were more active than monosubstituted product **MRP**. The slightly higher activity of **HRP** compared to **DRP** may be due to the coupling with PhB. In addition, the cytotoxicity of **MRP**, **DRP**, and **HRP** toward normal mouse hepatocytes was further evaluated. The results showed that the IC_50_ values of three Pt^IV^ prodrugs were significantly higher than that of CDDP, indicating lower toxicity toward normal cells while maintaining strong cytotoxicity against tumor cells (Table 1). These findings further demonstrate the favorable selectivity of the designed prodrugs and their potential to reduce systemic toxicity. Although previously reported Pt^IV^ prodrugs (such as Pt1‐Pt3) have been shown to induce pyroptosis through the GSDMD pathway and exhibit strong cytotoxicity against tumor cells, they also demonstrated high toxicity toward normal cells, resulting in limited therapeutic selectivity.^[^ ^26^ ^]^ In contrast, the Pt^IV^ prodrugs designed in this study activated pyroptosis via the GSDME pathway and effectively induced pyroptosis even in tumor cells with low GSDME expression, while exhibiting low toxicity toward normal cells. These features further emphasize the unique therapeutic potential and novelty of the strategy proposed in this study. Given the challenges of treating triple‐negative breast cancer and the greater sensitivity of breast cancer cell lines to the novel prodrugs, the mouse breast cancer cell line 4T1 was selected for detailed experiments to investigate the potential pathways in greater depth.

**Table 1:** IC_50_ (µm) of **CDDP**, RG108, **MRP**, **DRP**, and **HRP** against different colorectal cancer cell lines and Mice hepatocyte at 72 h^a)^.

Complexes	CDDP	RG108	MRP	DRP	HRP
4T1	11.90 ± 0.93	>125	4.14 ± 2.12	1.80 ± 1.01	0.59 ± 0.15
MDA‐MB‐231	21.02 ± 3.70	>125	10.21 ± 1.8	3.90 ± 0.52	2.07 ± 0.67
BT‐474	28.24 ± 3.82	>125	10.49 ± 2.18	3.86 ± 3.68	1.99 ± 0.19
MCF‐7	10.20 ± 1.82	>125	5.2 ± 1.24	1.3 ± 0.15	0.65 ± 0.11
T24	28.25 ± 2.81	>125	12.63 ± 6.06	5.21 ± 0.51	2.19 ± 0.13
A549	34.72 ±3.01	>125	10.32 ± 1.23	6.42 ± 0.67	4.46 ± 0.23
DU145	30.92 ± 1.52	>125	9.32 ± 0.89	5.10 ± 2.13	3.12 ± 1.56
Mice hepatocyte	48.6 ± 1.45	>500	79.8 ± 2.01	76.5 ± 0.98	68.9 ± 1.14

^a)^ C_50_ values are represented by mean SD of three independent experiments.

### 2.4 In Vitro Cell Proliferation and Migration Assay

**Figure 1:**
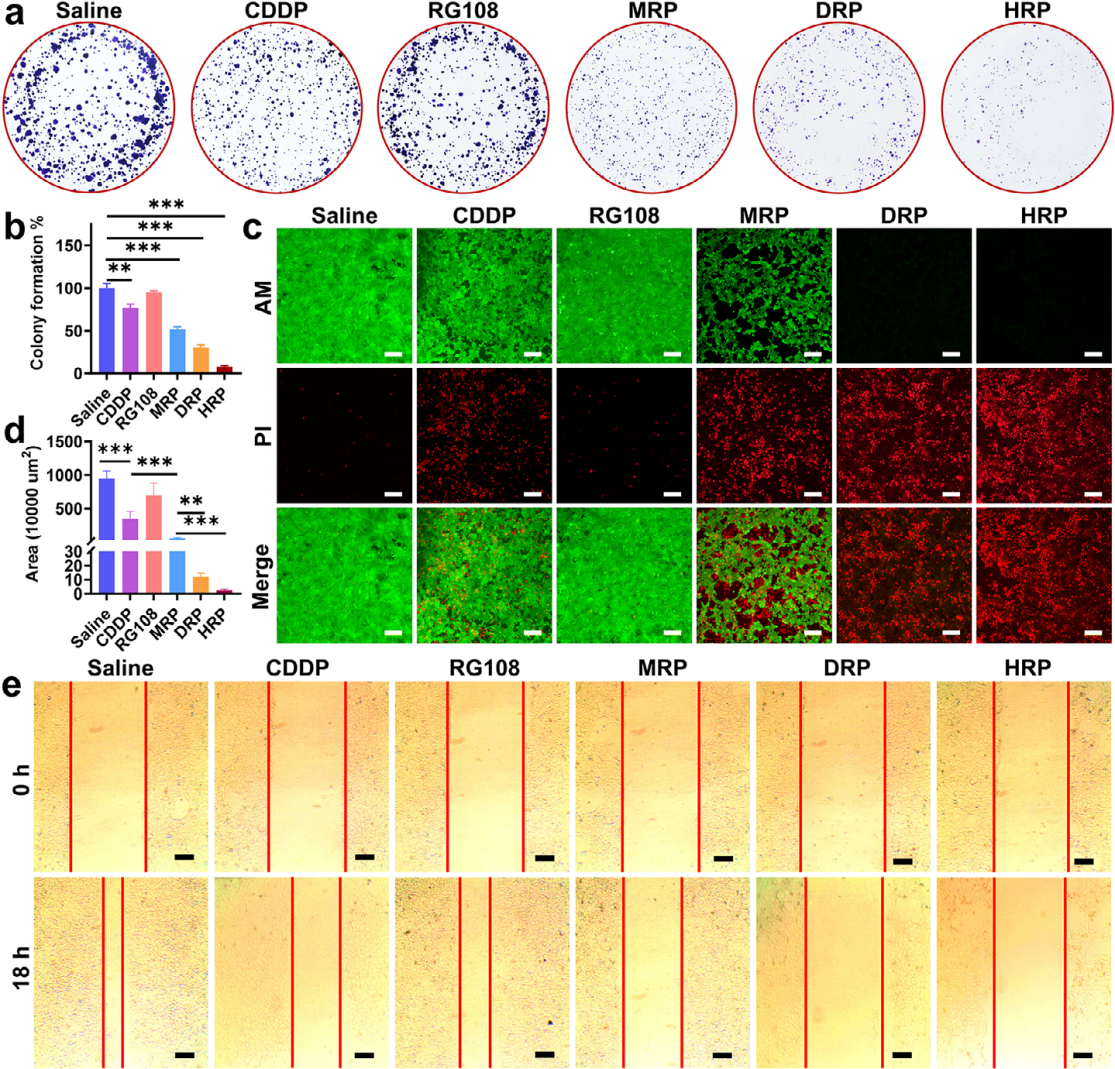
In vitro anti‐tumor activity of Pt^IV^ complexes. a,b) Colony formation assay of 4T1 cells after treated with different complexes (5 µm) (*n* = 3); c) Fluorescence images of 4T1 cells after 48 h treatment with different complexes (5 µm) via live/dead cell staining assay, scale bar: 200 µm. The cells were co‐stained with AM (green, live cells) and PI (red, dead cells); d,e) Migration results of 4T1 cells after 18 h treatment with different complexes (5 µm) (*n* = 4–5), Scale bar: 500 µm. One‐way ANOVA with Tukey's multiple comparison test (b, d). Statistical significance is demarked as *P < 0.05, **P < 0.01, and ***P < 0.001, throughout the text. Error bar indicated SEM.

The tumor cell inhibitory capacity of different complexes was further validated by a 4T1 cell colony formation assay. It was found that **MRP**, **DRP**, and **HRP** demonstrated a stronger ability to inhibit the proliferation of very‐low‐density tumor cells compared to **CDDP** (**Figure** **1**a,b), suggesting a wide range of destructive effects on tumorigenesis. Live and dead cells were further observed using calcein AM/propidium iodide (AM/PI) staining (Figure 1c). Compared to the control group, the RG108 group were predominantly green fluorescent, indicating that most of the cells were alive; the **MRP** group showed an increased number of red fluorescent‐stained cells than **CDDP** group, indicating more cell death. Notably, the **DRP** and **HRP** groups had a large number of red fluorescent‐stained cells, and the **HRP** group in particular, almost all of 4T1 cells were dead, suggesting that it had the strongest effect in inducing cell death. These results are consistent with the CCK8 antiproliferative assay.

**Figure 2:**
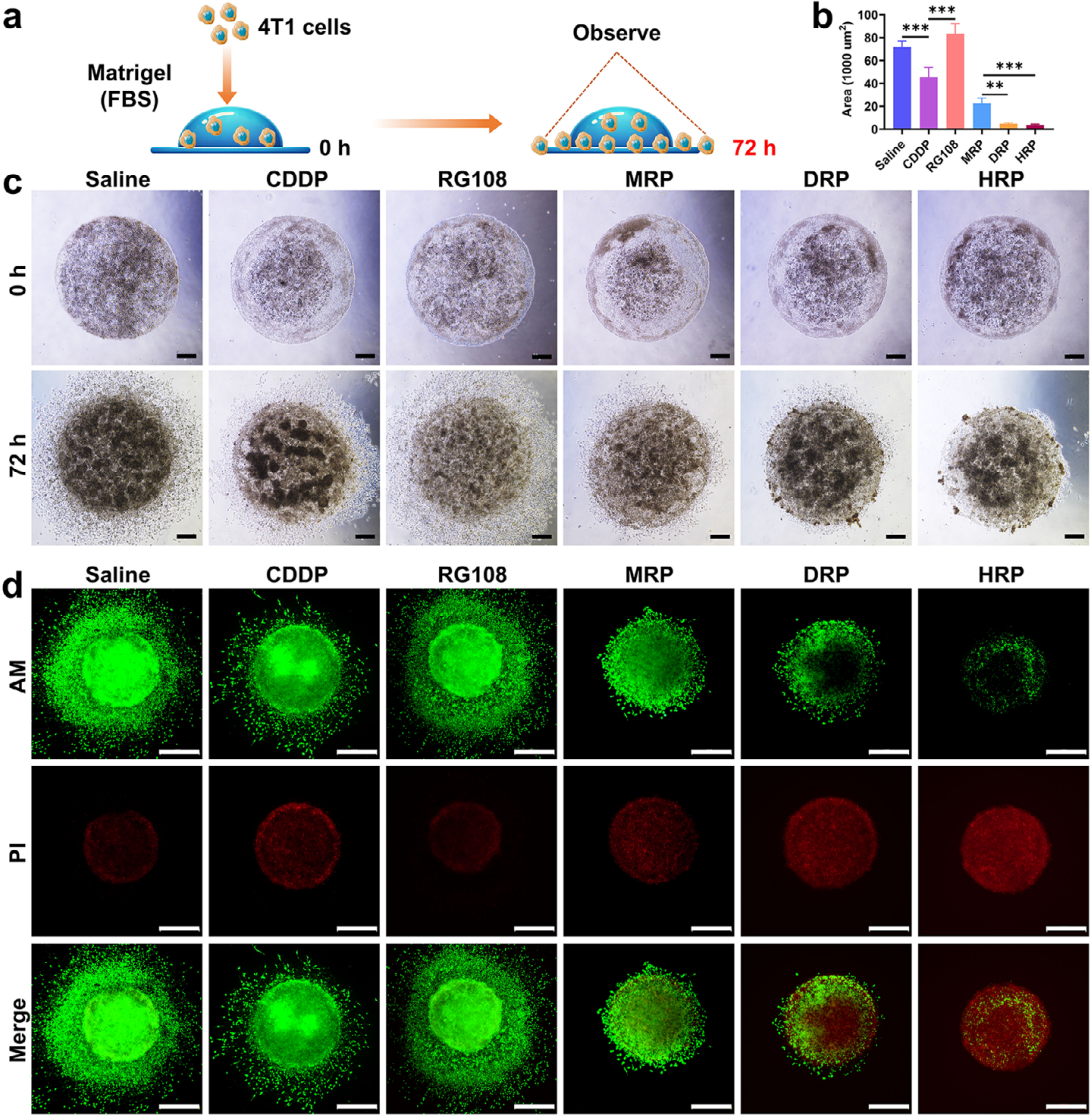
Tumor spheroid 3D migration assay of Pt^IV^ complexes. a) Schematic diagram of the tumor spheroid 3D migration assay; b,c) 3D tumor spheroid migration assay of 4T1 cells after 72 h in the presence of different complexes (5 µm) (*n* = 7–8), scale bar: 500 µm; d) Live/dead cell staining in 3D tumor spheroid migration assay of 4T1 cells after 72 h treatment with different complexes (5 µm), scale bar: 1000 µm. One‐way ANOVA with Tukey's multiple comparison test (b). Statistical significance is demarked as *P < 0.05, **P < 0.01, and ***P < 0.001, throughout the text. Error bar indicated SEM.

The effects of **MRP**, **DRP**, and **HRP** on 4T1 cell migration were subsequently investigated using wound healing assays. Experimental results showed that the gap at the edge of the scratch was significantly reduced after 24 h. The wound healing rates of control, **CDDP**, RG108, **MRP**, **DRP**, and **HRP** were 100%, 63.65%, 116.11%, 31.53%, 6.65%, and 5.43%, respectively (Figure 1d,e). These findings indicated that **MRP**, **DRP**, and **HRP** significantly inhibited the motility and migration ability of 4T1 cells, with **HRP** being the most effective. Compared with wound healing experiments, 3D migration (**Figure** **2**a) experiments provide a more realistic picture of cell migration and invasion behavior. The effects of **MRP**, **DRP**, and **HRP** on the migratory and invasive abilities of 4T1 tumor cells in the surrounding stroma were assessed using a 3D spherical model (Figure 2b,c). After transferring the newly generated cells into Matrigel and incubating them, it was obvious that compared with **CDDP**, the migration process of the cells in the model was significantly slowed down after treatment with **MRP**, **DRP**, and **HRP**. In comparison, RG108 alone displayed a minimal inhibitory effect on 4T1 cell migration. **HRP** exhibited the strongest inhibitory effect on migration, and the number of migrating cells around the spheroids was almost negligible and only a minimal number of cells survived (Figure 2d). These findings indicated that **MRP**, **DRP**, and **HRP** not only effectively inhibited the proliferative activity of 4T1 cells but also greatly restricted the migration and invasion ability of 4T1 cells, and **HRP** has the most obvious effect.

### 2.5 Transcriptomic Analysis

**Figure 3:**
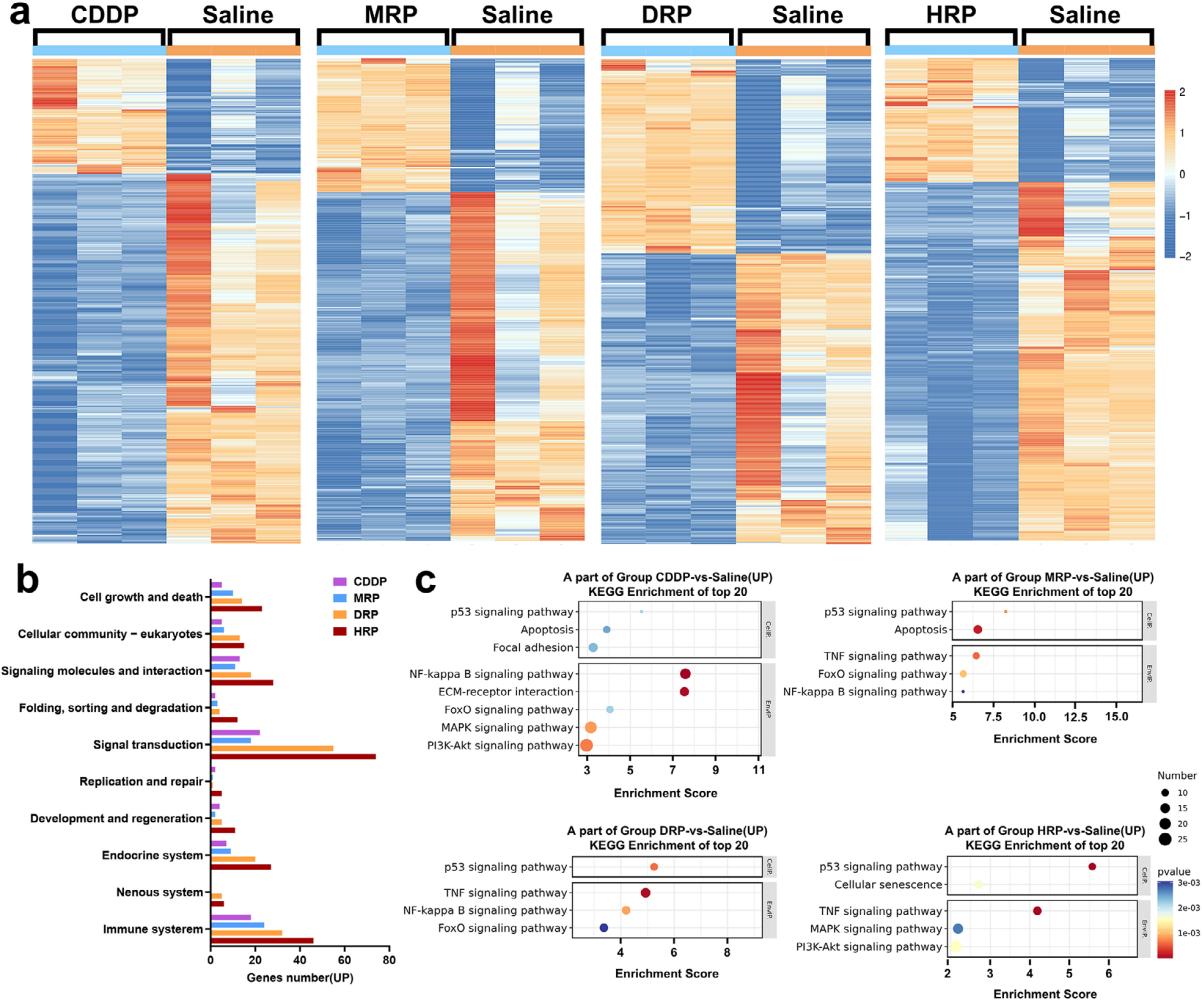
Differential gene expression in 4T1 cells treated with CDDP and Pt^IV^ complexes. a) Comparison of gene expression regulation before and after treatment with saline, **CDDP**, **MRP**, **DRP**, and **HRP**. Hierarchical cluster analysis of 860, 698, 1002, and 2474 differentially expressed mRNAs with statistically significant differences (P < 0.05, fold change > 2 or < 0.5) in **CDDP**‐, **MRP**‐, **DRP**‐, and **HRP**‐treated 4T1 cells, compared to untreated (Saline) cells. b) Three independent experiments were conducted for saline, **CDDP**, **MRP**, **DRP**, and **HRP** treatments; KEGG pathway classification analysis: 10 pathways showing obvious enrichment (logP value > 2) were selected. Gene count: number of differentially expressed genes mapped to each pathway; c) A subset of the top 20 KEGG enrichment results for the Saline, **MRP**, **DRP**, and **HRP** groups.

To further understand the action mechanism of **MRP**, **DRP**, and **HRP**, differentially expressed mRNAs among **CDDP**‐, **MRP**‐, **DRP**‐, **HRP**‐treated, and control cells (4T1) were characterized using mRNA sequencing. The actual data volumes for each sample ranged from 6.88 to 7.06 G, with Q30 base distribution between 94.83% and 95.35% and an average GC content of 50.70%. The genome alignment rates for each sample were determined by comparing the readings to the reference genome, ranging from 97.35% to 97.95%. According to the alignment results, the expression levels of protein‐coding genes were analyzed. Differential screening was performed according to the expression levels of protein‐coding genes in different samples. Compared to the control group, 860, 698, 1002, and 2473 differential genes were detected in the **CDDP**, **MRP**, **DRP**, and **HRP** groups, respectively. Q value < 0.05 and fold change > 2 or fold change < 0.5 were set as the threshold for significantly differential expression genes (DEGs). All expression values of these differentially expressed genes were converted to a log form and input as hierarchical clustering algorithms (**Figure** **3**a), revealing 244 up‐regulated and 656 down‐regulated proteins in the case of **CDDP**, 194 up‐regulated and 504 down‐regulated proteins in the case of **MRP**, and 401 up‐regulated and 635 down‐regulated proteins in the case of **DRP**, and 601 up‐regulated and 1838 down‐regulated proteins in the case of **HRP**.

According to gene ontology (GO), the most prevalent biological processes in which these differentially expressed genes participate are oxidation‐reduction and biomolecule metabolic processes, including nucleobase‐containing small molecule, nucleotide, nucleoside phosphate, ribose phosphate, ribonucleotide, and rRNA metabolism, etc. (Figures S32–S35, Supporting Information). Kyoto Encyclopedia of Genes and Genomes (KEGG) analysis found that **MRP**, **DRP**, and **HRP** upregulated approximately ten signaling pathways, including those related to the immune system, nervous system, cell growth and death, and signal transduction, compared with **CDDP** (Figure 3b). Additionally, **MRP**, **DRP**, and **HRP** treatments significantly upregulated the TNF signaling pathway, which was often associated with physiological processes such as inflammation and immune responses in 4T1 cells compared to **CDDP** treatment (Figure 3c; Figures S36–S39, Supporting Information). The results suggested that **MRP**, **DRP**, and **HRP** might promote the expression of inflammatory factors, potentially related to the inflammatory response triggered by pyroptosis.

### 2.6 In Vitro Validation of Pyroptosis Induction

**Figure 4:**
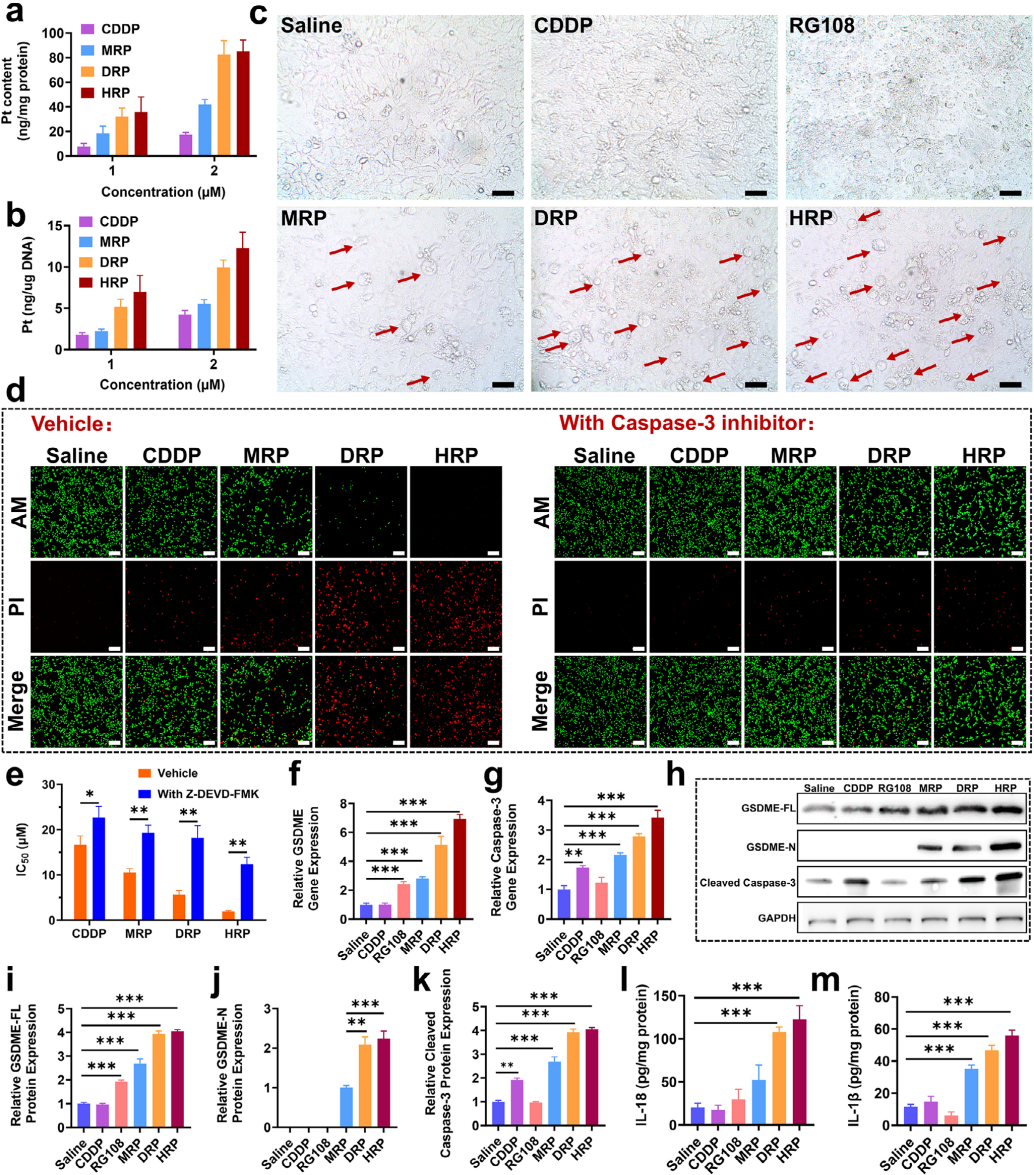
Verification of the pyroptosis mechanism mediated by Pt^IV^ complexes. Cellular (a) and DNA (b) accumulation of platinum in 4T1 cells after treatment with 1 and 2 µm (0.5‰ v/v DMSO) of **MRP**, **DRP**, **HRP**, and **CDDP** for 24 h (*n* = 3); c) Representative photographs of 4T1 cells treated with different complexes (5 µm). Red arrows indicate pyroptotic cells. Scale bar: 100 µm; d) Live/dead cell staining assay of 4T1 cells after 48 h treatment with different complexes (5 µm) with or without Z‐DEVD‐FMK, scale bar: 200 µm. e) IC_50_ (µm) of different complexes against 4T1 cells with or without Z‐DEVD‐FMK; f,g) qPCR statistical analysis of GSDME and Caspase‐3 (*n* = 6); h–k) Protein expression levels of GSDME‐FL, GSDME‐N, and Cleaved Caspase‐3, in 4T1 cells after treatment with different complexes (5 µm) (*n* = 6); l,m) ELISA detection of IL‐18 and IL‐1β release in the supernatant (*n* = 3–6). One‐way ANOVA with Tukey's multiple comparison test (a, b, f, g, i, j, k, l, m), unpaired Student's t test (e). Statistical significance is demarked as *P < 0.05, **P < 0.01, and ***P < 0.001, throughout the text. Error bar indicated SEM.

The cellular mechanisms of **MRP**, **DRP**, and **HRP** were further explored due to their superior antiproliferative abilities over **CDDP**. The accumulation of **MRP**, **DRP**, and **HRP** in 4T1 cells was assessed by measuring platinum (Pt) content via ICP‐MS (**Figure** **4**a) and the results showed that at a concentration of 2 µm, the cellular uptake of **MRP**, **DRP**, and **HRP** was ≈2.5, 4.8, and 5.0‐fold higher than that of **CDDP**, respectively. **HRP** and **MRP** exhibited nearly identical uptake levels, which could be attributed to their similar lipophilicity. The effects of **MRP**, **DRP**, and **HRP** on the intracellular DNA of 4T1 cells were further investigated (Figure 4b). The levels of intracellular platinum accumulation in DNA correlated with the initial concentration of the complexes and cellular uptake in the order of **HRP** > **DRP** > **MRP** > **CDDP**, consistent with their in vitro antiproliferative activities. Although **MRP** was reduced faster than **DRP**, it induced lower DNA platination and antiproliferative activity, suggesting the reduction kinetics of Pt^IV^ prodrugs were not the determining factors for DNA platination and cytotoxicity. Notably, despite having chromatin caused by PhB, which facilitated the attachment of active Pt^II^ fragments to DNA, resulting in a stronger antiproliferative effect. Observation of the morphological characteristics of 4T1 cells after treatment with different drugs revealed that cells treated with **CDDP** and RG108 exhibited varying degrees of collapse or atrophy (Figure 4c). Interestingly, cells treated with **MRP**, **DRP**, and **HRP** gradually expanded and formed many bubble‐like protrusions (Figure 4c). These different cellular morphological features indicated that cell death in the control, **CDDP**, and RG108 groups was due to apoptosis, while cell death induced by **MRP**, **DRP**, and **HRP** was characterized by pyroptosis. In addition, more pyroptotic cells were observed in the **HRP**‐treated groups (Figure S40, Supporting Information), further confirming the greatest cytotoxicity of **HRP**.

To further verify the pyroptosis of 4T1 cells was caused by Caspase‐3 cleavage GSDME, Caspase‐3 specific inhibitor Z‐DEVD‐FMK was used to treat different groups to further confirm the role of Caspase‐3 in the cell death process. It was clearly observed that live/dead staining revealed that the degree of cell death in **MRP**, **DRP**, and **HRP**‐treated cells markedly reduced after treatment with Z‐DEVD‐FMK (Figure 4d). Inhibition of Caspase‐3 activity was also significantly reduced cytotoxicity in **MRP**, **DRP**, and **HRP**‐treated 4T1 cells (Figure 4e; Table S2, Supporting Information). This result indicated that pyroptosis induced by **MRP**, **DRP**, and **HRP** was closely related to the activation of Caspase‐3. In the GSDME‐induced pyroptosis pathway, the cleavage of GSDME by Caspase‐3 to generate GSDME‐N is critical for the induction of pyroptosis. Western blot results showed that, compared with the control group, the expression of Caspase‐3 in 4T1 cells treated with **CDDP** alone increased significantly with increasing **CDDP** concentration (Figure S41, Supporting Information). Meanwhile, the expression of the GSDME protein almost disappeared, likely due to methylation of the GSDME gene promoter (Figure 4h,i), which is consistent with the characteristics of **CDDP**‐induced apoptosis. Expectedly, the expression of GSDME increased dose‐dependently when 4T1 cells were treated with RG108 alone, confirming that RG108 was a potent inhibitor of DNMT (Figure S42, Supporting Information). Compared with **CDDP** treatment, **MRP**, **DRP**, and **HRP** treatments significantly increased GSDME expression, along with the presence of GSDME‐N and cleaved Caspase‐3 fragments (Figure 4h**–k**). Compared to the **MRP** group, the expression of GSDME protein (GSDME‐FL + GSDME‐N) and cleaved Caspase‐3 in the **DRP** group was higher, which was consistent with the qPCR data (Figure 4f,g). This difference may be due to the number of RG108 conjugates in the **DRP** molecule. At the same drug concentration, the uptake of cells in the **DRP** group was higher than that in the **MRP** group, releasing more RG108 and **CDDP**, which promoted the expression of GSDME and Caspase‐3 proteins. Interestingly, although the uptake of cells in the **HRP** group was only slightly higher than that in the **DRP** group, and there was only one RG108 molecule in the **HRP** molecule, the **HRP** group expressed higher levels of GSDME protein (Figure 4h–j). This may be attributed to the conjugation of PhB in the **HRP** molecule, which relaxes the chromatin in the cell and thus improves the transcription efficiency of the GSDME gene. Based on the results from both western blot and qPCR, it was found that the **HRP** group also expressed higher levels of Caspase‐3 than the **DRP** group (Figure 4h,k). Under the combined action of GSDME and Caspase‐3, the **HRP** group expressed higher levels of GSDME‐N protein, which is a key factor in directly inducing pyroptosis. The changes in the level of GSDME‐N indicated that **MRP**, **DRP**, and **HRP** could effectively utilize the Caspase‐3/GSDME pathway to transition cell death from apoptosis to pyroptosis. In addition, **HRP** induced the highest levels of GSDME‐N fragments and cleaved Caspase‐3 than **MRP** and **DRP**, suggesting that it was more effective in inducing pyroptosis in 4T1 cells.

During pyroptosis, the pro‐inflammatory factors IL‐18 and IL‐1β are activated and released into the tumor microenvironment, enhancing the inflammatory response. Analysis of the released IL‐18 and IL‐1β in the different treatment groups by ELISA showed that the levels of IL‐18 and IL‐1β production in 4T1 cells treated with **CDDP** and RG108 were not significantly different from those in the control group (Figure 4l,m). However, the production of these pro‐inflammatory factors was dramatically increased in the **MRP**, **DRP**, and **HRP** groups, with the **HRP** group showing the greatest increase, which was also consistent with the qPCR data (Figure S43, Supporting Information).

**Figure 5:**
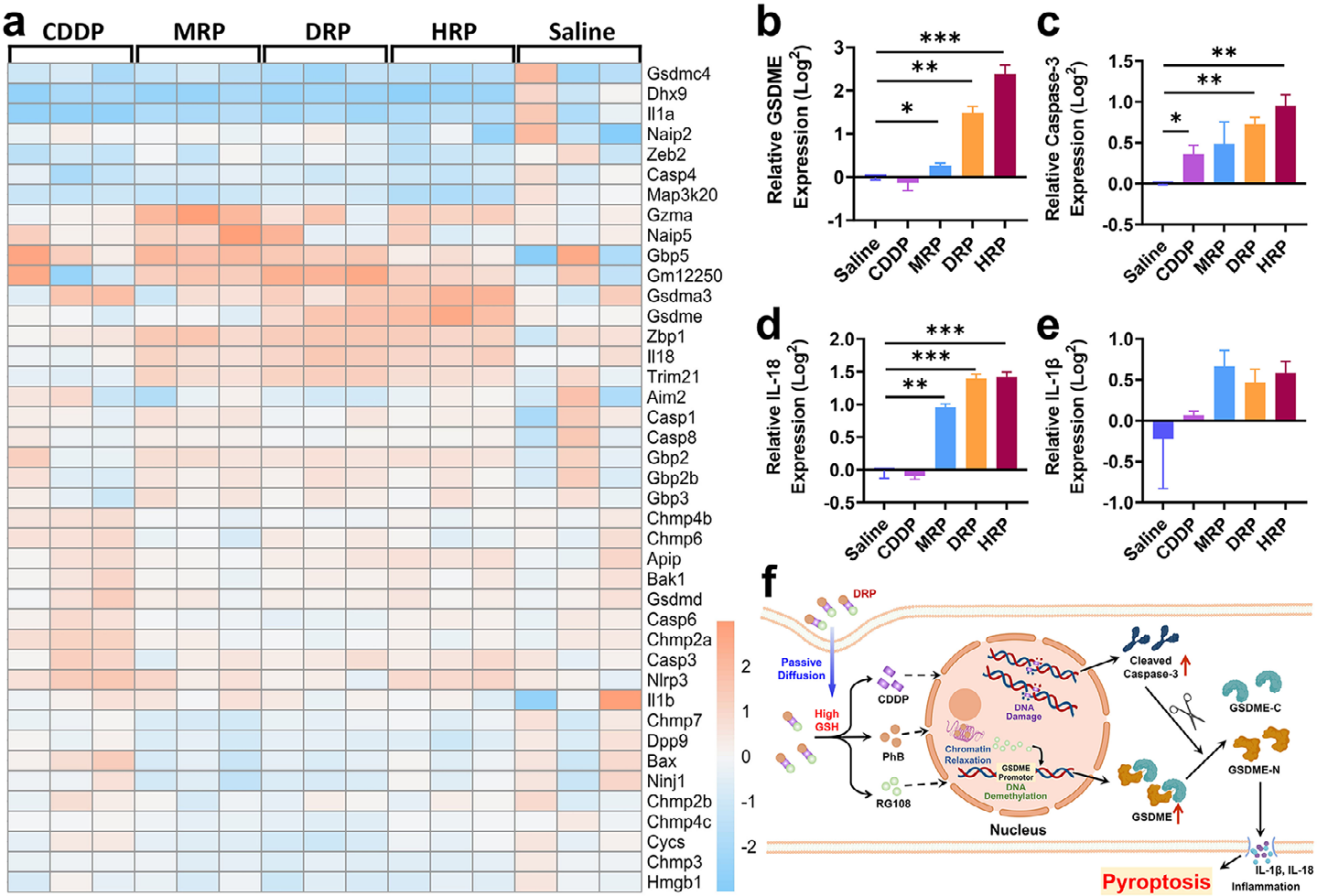
Expression of pyroptosis‐related protein genes. a) Comparison of gene expression regulation before and after treatment with saline, **CDDP**, **MRP**, **DRP**, and **HRP**; Expression levels of pyroptosis‐related proteins GSDME (b), Caspase‐3 (c), and cytokines IL‐18 (d), IL‐1β (e) (*n* = 3); f) Diagram illustrating the mechanism of pyroptosis induction by **HRP** in 4T1 cells. One‐way ANOVA with Tukey's multiple comparison test (b, c, d, e). Statistical significance is demarked as *P < 0.05, **P < 0.01, and ***P < 0.001, throughout the text. Error bar indicated SEM.

Analysis of the expression of protein‐coding genes showed that compared with the **CDDP** group, the expression of GSDME in 4T1 cells treated with **MRP**, **DRP**, and **HRP** was significantly increased (**Figure** **5**a,b), with the **HRP** group being particularly notable. This result is consistent with the western blot assay, which further demonstrates that PhB in **HRP** promotes the transcriptional efficiency of GSDME by inducing chromatin relaxation. In addition, the expression of Caspase‐3 in the **HRP** group was also significantly higher than that in the **MRP** and **DRP** groups (Figure 5c), indicating that **HRP** has the strongest effect in inducing pyroptosis based on the Caspase‐3/GSDME pathway. Meanwhile, the expression of the inflammatory cytokines IL‐18 and IL‐1β was significantly upregulated (Figure 5d,e), which is well aligned with the in vitro experimental findings. These results indicated that these novel prodrugs can effectively induce pyroptosis in 4T1 cells and activate the intracellular inflammatory response, further enhancing their anti‐tumor effect. Notably, **HRP** exhibited the best efficacy, and the possible mechanisms of pyroptosis induction by **HRP** in 4T1 cells is shown in Figure 5f.

### 2.7 In Vivo Anti‐Tumor Activity

**Figure 6:**
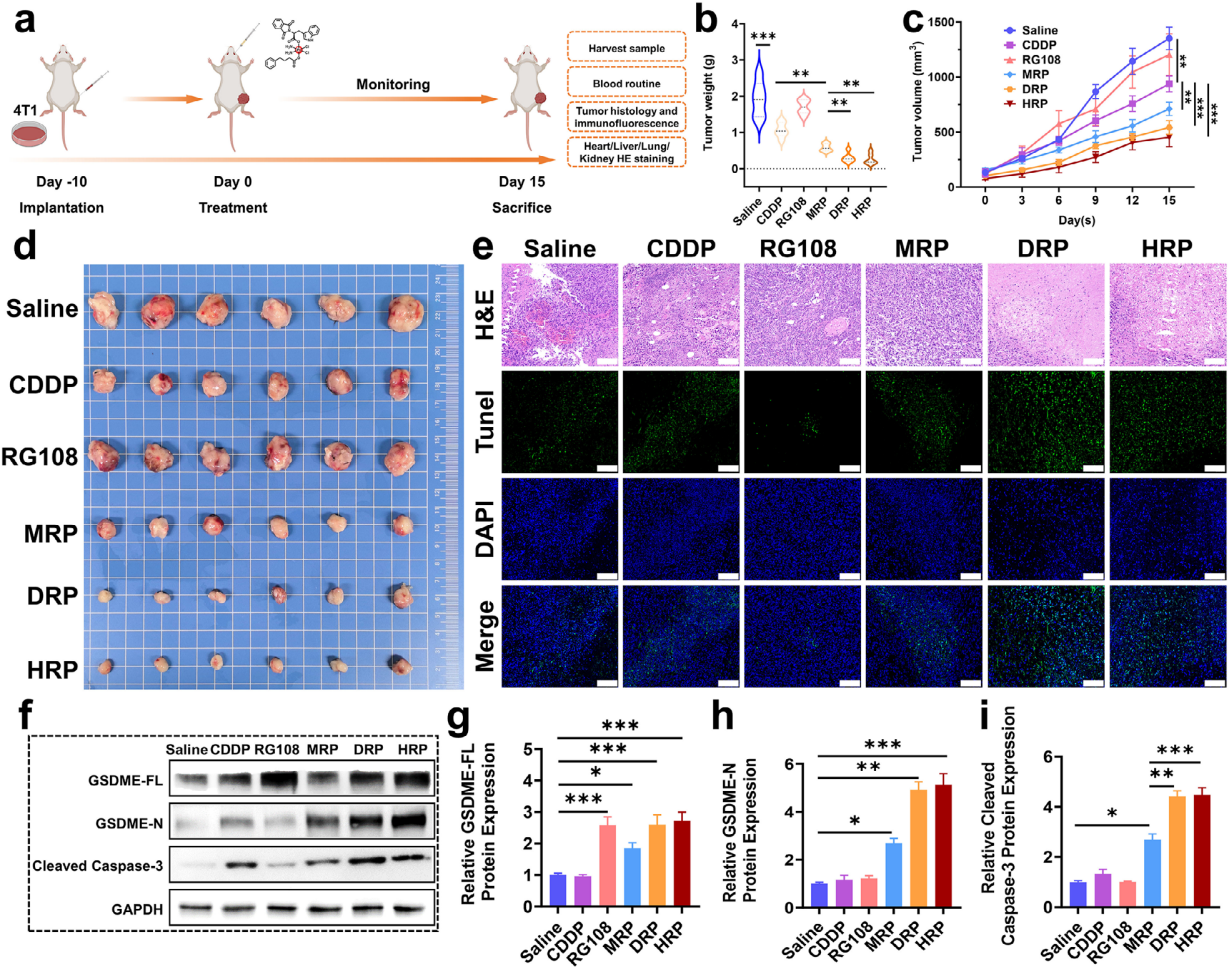
Anti‐tumor effects of Pt^IV^ complexes in vivo. a) Schematic of in vivo anti‐tumor procedures using **HRP** as an example; b) Tumor weight after various treatments on day 15, data are presented as mean ± standard deviation (*n* = 6); c) Tumor growth curves of 4T1 tumor‐bearing mice in corresponding treatment groups (*n* = 6); d) Tumor size in mice from each treatment group after 15 days of treatment; e) H&E and TUNEL staining of tumor slices after treatments (Scale bar: 100 µm); f–i) Protein expression levels of GSDME‐FL, GSDME‐N, and Cleaved Caspase‐3, in tumor tissues (*n* = 6). One‐way ANOVA with Tukey's multiple comparison test (b, g, h, i), Two‐way repeated measures ANOVA Bonferroni's multiple comparisons test (c). Statistical significance is demarked as *P < 0.05, **P < 0.01, and ***P < 0.001, throughout the text. Error bar indicated SEM.

To better verify the antitumor efficacy of **MRP**, **DRP**, and **HRP** in vivo, especially their immunological effects, BALB/c mouse model was used for treatment studies. The treatment flow chart is shown in **Figure** **6**a. After 15 days of treatment, **MRP**, **DRP**, and **HRP** showed more pronounced growth inhibition, with tumor volumes reduced to 711.01 ± 134.69 mm^3^, 542.07 ± 151.37 mm^3^ and 453.61 ± 208.97 mm^3^, respectively, and tumor weights reduced to 0.59 ± 0.11 g, 0.30 ± 0.13 g and 0.26 ± 0.14 g, respectively (Figure 6b–d). In comparison, the tumor volumes of the control group, **CDDP**, and RG108, were 1351.27 ± 249.78 mm^3^, 937.66 ± 175.11 mm^3^, 1203.30 ± 462.30 mm^3^, and the tumor weights were 1.92 ± 0.49 g, 1.07 ± 0.21 g, 1.71 ± 0.20 g respectively. These data confirmed that **MRP**, **DRP**, and **HRP** displayed substantial antitumor efficacy in vivo, with **HRP** showing the most pronounced effect. To further verify the mechanism of action, the tumor tissues were stained with hematoxylin and eosin (H&E) and TdT‐mediated dUTP nick end labeling (TUNEL) (Figure 6e). The H&E results showed that the tumor cells in the **DRP** and **HRP** groups exhibited more obvious structural changes and were in a worse condition than those in the **CDDP** and **MRP** groups. The results indicated tumor cell deaths increased during **DRP** and **HRP**‐ mediated pyroptosis treatment. The TUNEL staining results were consistent with the H&E staining results, suggesting that there were a large number of pyroptotic/apoptotic cells with green fluorescent staining in the **DRP** and **HRP** treatment groups, while fewer green fluorescent cells were observed in the other groups (Figure S44, Supporting Information). These findings further confirm that **DRP** and **HRP** have a prominent ability to induce pyroptosis and an outstanding therapeutic effect.

Western blot analysis demonstrated that RG108, **MRP**, **DRP**, and **HRP** could increase the expression level of GSDME (GSDME‐FL + GSDME‐N) in tumor tissue cells, but, unlike the others, RG108 did not significantly increase the expression of Caspase‐3. Compared with **CDDP** alone, **MRP**, **DRP**, and **HRP** not only effectively activated Caspase‐3 in tumor tissue cells but also activated GSDME, producing more GSDME‐N, which directly triggered pyroptosis (Figure 6f–i). In addition, compared with **MRP** and **DRP**, **HRP** induced the highest levels of GSDME and Cleaved Caspase‐3 proteins, and produced the greatest amount of GSDME‐N, which was consistent with the protein blot data from in vitro experiments. The change in GSDME‐N levels indicated that in vivo. **MRP**, **DRP**, and **HRP** can all effectively switch cell death mode from apoptosis to pyroptosis via the Caspase‐3/GSDME pathway, with **HRP** showing the most pronounced effect. In addition, immunofluorescence staining of HMGB1 in tumor tissues showed that HMGB1 was translocated from the nucleus to the cytoplasm and extracellular space in the **MRP**, **DRP**, and **HRP** treatment groups compared to the **CDDP** group, indicating effective pyroptosis induction by these Pt^IV^ prodrugs. Notably, the **HRP**‐treated group exhibited the weakest nuclear fluorescence signal of HMGB1, suggesting the highest degree of HMGB1 release, which further supported the prominent role of **HRP** in promoting DAMPs release and immune activation (Figure S45, Supporting Information).

### 2.8 Antitumor Immunity in Vivo

**Figure 7:**
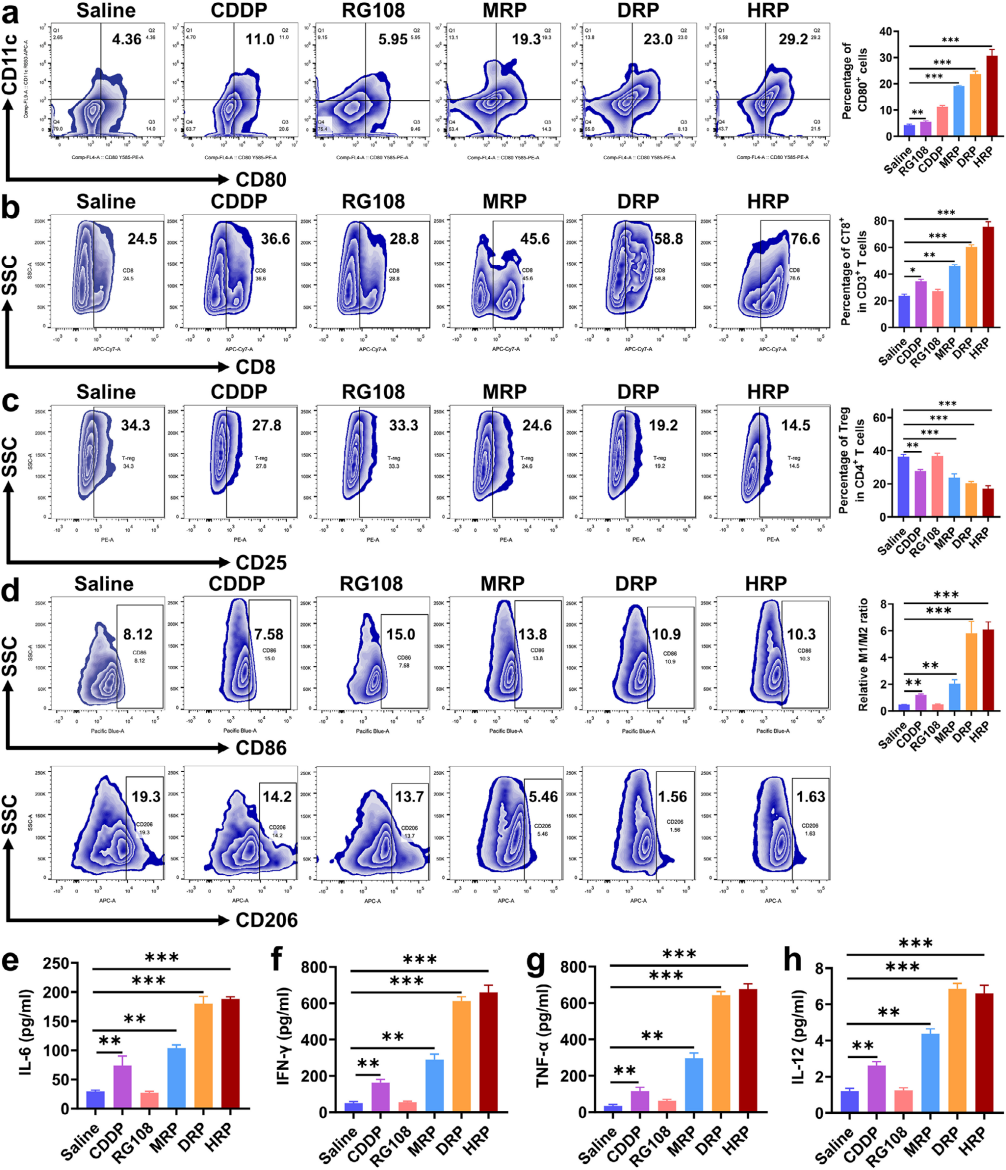
The immune activation effect of Pt^IV^ complexes in vivo. a) Flow cytometry results and quantitative analysis of dendritic cells (CD11b^+^, CD11c^+^, CD80^+^) in 4T1 tumors on day 10 (*n* = 5); b) Flow cytometry results of CD4^+^ (CD3^+^, CD4^+^) and CD8^+^ (CD3^+^, CD8^+^) T cells, including the quantitative analysis of CD8^+^ T cells in 4T1 tumors on day 10 (*n* = 5); c) Flow cytometry results and quantitative analysis of Tregs (CD3^+^, CD4^+^, CD25^+^) in 4T1 tumors on day 10 (*n* = 5); d) Flow cytometry results and quantitative analysis of the M1(CD11b^+^, F4/80^+^, CD86^+^)/M2 (CD11b^+^, F4/80^+^, CD206^+^) macrophage ratio in 4T1 tumors on day 10 (*n* = 5); e–h) The contents of IL‐6 (e), IFN‐γ (f), TNF‐α (g), and IL‐12 (h) in 4T1 tumor tissues after different treatments on day 10, (*n* = 6). One‐way ANOVA with Tukey's multiple comparison test. Statistical significance is demarked as *P < 0.05, **P < 0.01, and ***P < 0.001, throughout the text. Error bar indicated SEM.

To verify the immune effects of **MRP**, **DRP**, and **HRP** in inducing tumor cell pyroptosis, the changes in several immune cells at the tumor site were further evaluated. As shown in **Figure** **7**a, the levels of mature dendritic cells (DCs, CD11c^+^ CD80^+^) in the **MRP**, **DRP**, and **HRP** treatment groups were higher than those in the **CDDP** treatment group. Among them, the **HRP** group had the highest proportion, reaching 29.2%, which was significantly higher than that of the other groups. In comparison, the proportions of mature DCs in the control group, **CDDP** group, RG108 group, **MRP** group, and **DRP** group were 4.36%, 11.0%, 5.95%, 19.3%, and 23.0%, respectively. In addition, the ability of **MRP**, **DRP**, and **HRP** to activate CD8^+^ cytotoxic T lymphocytes (CTLs) was further studied. Compared with the **CDDP** treatment group, the rate of CD8^+^ T cells in the **MRP**, **DRP**, and **HRP** treatment groups was significantly increased, with the highest expression levels in the **HRP** group, which was close to 76.6% (Figure 7b). The results showed that during tumor treatment, **MRP**, **DRP**, and **HRP** are more effective at activating the anti‐tumor immune response than **CDDP**, with **HRP** showing the greatest effect. The tumor immune microenvironment is critical during tumor therapy. As shown in Figure 7c, **MRP**, **DRP**, and **HRP** had a greater inhibitory impact on Tregs than **CDDP** and RG108, with **DRP** and **HRP** being the most effective. Additionally, the M1/M2 ratio was considerably higher in the **DRP** and **HRP** groups compared to the other groups (Figure 7d). These results suggested that **DRP** and **HRP** might efficiently transform M2 macrophages, which promote tumor development and immune evasion, into M1 macrophages, which suppress tumor proliferation. This result also meant that after **DRP** and **HRP** treatment, the tumor microenvironment was effectively changed. Key biomarkers such as TNF‐α, IL‐6, IL‐12, and IFN‐γ play a key role in activating immune cells, enhancing antigen presentation, regulating the tumor microenvironment, promoting immune responses, and inhibiting tumor growth. Compared with other treatment groups, **DRP** and **HRP** treatment significantly increased the levels of these biomarkers (Figure 7e–h). The significant increase in TNF‐α was also consistent with the trend of gene analysis, indicating that **DRP** and **HRP** triggered a more powerful anti‐tumor immune response in vivo.

All these data revealed that, compared to **CDDP** alone, **MRP**, **DRP**, and **HRP** increased the number of innate and adaptive immune cells, such as CD8^+^ T cells and dendritic cells, by causing pyroptosis in tumor cells via the Caspase‐3/GSDME pathway. At the same time, they efficiently reduced the number of Tregs and promoted the development of macrophages from the M2 phenotype to the M1 phenotype, thereby turning the immunosuppressive “cold” TME into an immunogenic “hot” TME and activating tumor immune effects. It is worth mentioning that **DRP** and **HRP** exhibited a more pronounced immune activation effect compared to **MRP**.

### 2.9 Inhibition of Tumor Metastasis

**Figure 8:**
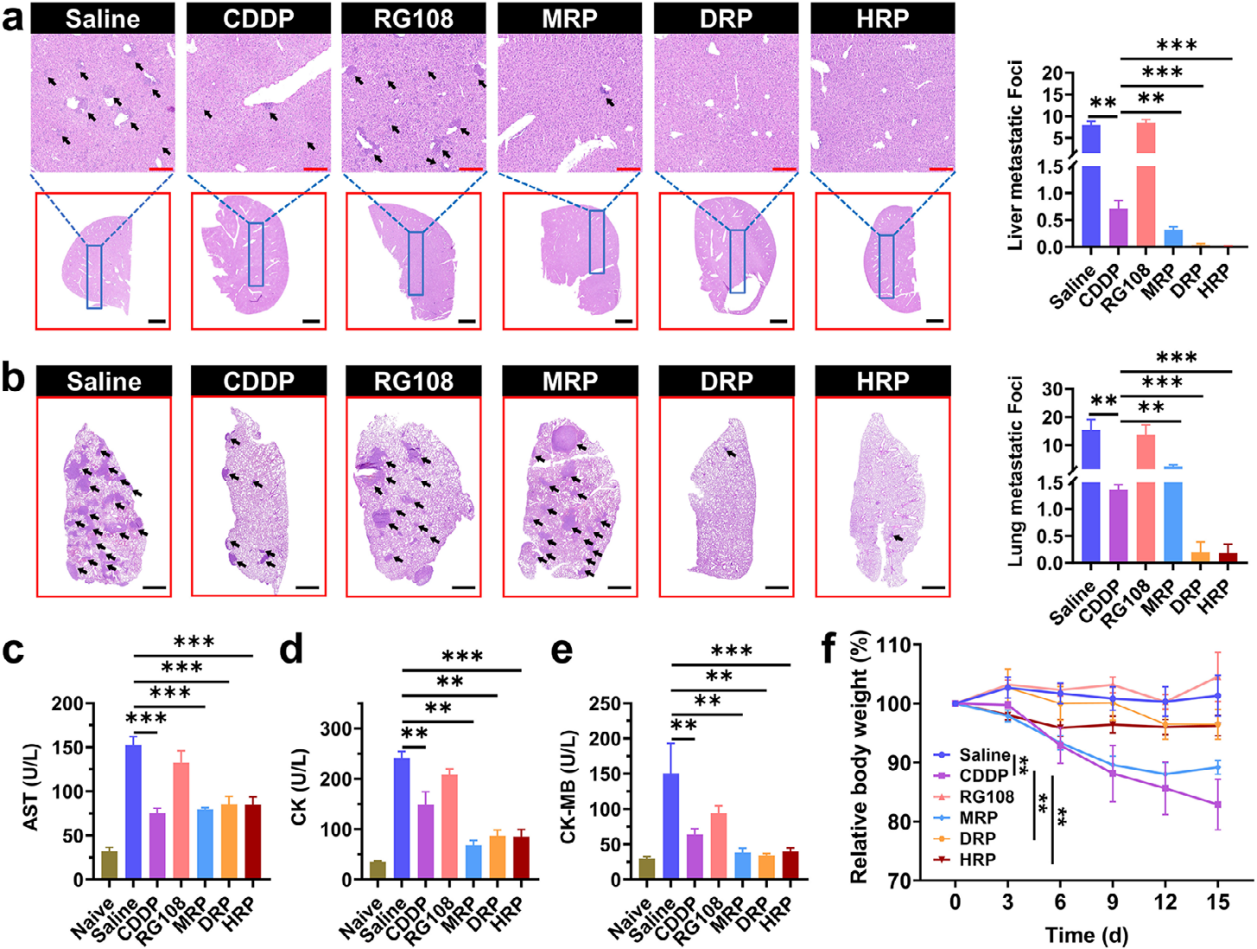
Antitumor metastasis capability and safety assessment of Pt^IV^ complexes in vivo. a) H&E‐stained images of liver tissues after different treatments, black arrows point to major metastases, scale bars: up, 100 µm, down, 2000 µm (*n* = 6); b) H&E‐stained images of lung tissues after different treatments, with black arrows pointing to major metastases, scale bar: 1000 µm (*n* = 6); c) Generation levels of AST in different treatment groups (*n* = 5–7); d) Levels of CK in different treatment groups (*n* = 5–7); e) Generation levels of CK‐MB in different treatment groups (*n* = 5–7); f) Body weight variation curves of 4T1 tumor‐bearing mice in corresponding treatment groups (*n* = 6). One‐way ANOVA with Tukey's multiple comparison test (a, b, c, d, e), Two‐way repeated measures ANOVA Bonferroni's multiple comparisons test (f). Statistical significance is demarked as *P < 0.05, **P < 0.01, and ***P < 0.001, throughout the text. Error bar indicated SEM.

To further investigate the ability of these drugs to inhibit tumor metastasis, H&E staining was used to detect the liver, lung, kidney, and heart tissues of mice after different treatments, and the relevant biochemical indicators were measured (**Figure** **8**a–e; Figures S46–S48, Supporting Information). Results showed that in the control group and the group treated with RG108 or **CDDP** alone, there were many metastases in the lung and liver tissues of the mice, the tumor cells were widely distributed (Figure 8a,b), and the aspartate aminotransferase (AST) was significantly higher than normal(Figure 8c). Although no obvious metastases were observed in heart and kidney sections, the levels of creatine kinase (CK) and creatine kinase‐MB (CK‐MB) were abnormally high, indicating damage to the heart or skeletal muscle at the end of treatment (Figure 8d,e). In comparison, mice treated with **DRP** and **HRP** showed significantly reduced metastases in the lung and liver tissues, and their AST, CK, and CK‐MB levels were also reduced considerably, approaching the levels of normal mice, indicating a better inhibitory effect on tumor metastasis. It is worth noting that there was almost no metastasis in the **DRP** and **HRP** group, suggesting that **MRP**, **DRP**, and **HRP** can activate the immune response and effectively inhibit tumor metastasis, with **DRP** and **HRP** being more effective. In addition, BALB/c mice treated with **CDDP** at a dose of 1.5 mg kg^−1^ showed a significant decrease in body weight, which dropped to 80% of the initial body weight after treatment, while mice treated with **MRP** dropped to 90% of the initial body weight (Figure 8f). In comparison, **DRP** and **HRP** had little effect on the body weight of the mice.

Comprehensive in vivo experimental data demonstrated that, compared with **CDDP**, **HRP** not only effectively induced tumor immunity, significantly suppressed tumor growth and metastasis but also reduced the systemic toxicity of chemotherapy to normal tissues. These results suggested that **HRP** held promise for advancing cancer therapies with improved efficacy and safety profiles.

## 3 Conclusion

In this study, we successfully designed and synthesized three Pt^IV^ prodrugs, **MRP**, **DRP**, and **HRP**, that could induce pyroptosis in tumor cells by activating the Caspase‐3/GSDME pathway. These prodrugs exhibited stronger cytotoxicity than **CDDP** in several tumor cell lines with low GSDME expression. **HRP** demonstrating the most prominent killing ability, which could amplify the inflammatory response through effectively increasing the production of pro‐inflammatory cytokines IL‐18 and IL‐1β, thereby enhancing the inflammatory response. mRNA sequencing revealed that these prodrugs significantly upregulated pathways related to the immune and nervous systems, cell proliferation, and signal transduction, especially enhancing the TNF signaling pathway. In vivo studies in BALB/c mice further confirmed that **DRP** and **HRP** significantly reduced tumor volume and metastatic lesions and caused less systemic toxicity and organ damage than **CDDP**, with **HRP** being the most effective. In addition, **HRP** treatment enhanced the activity of immune cells in tumor tissue, accelerated the maturation of dendritic cells, increased the number of CD8^+^ T cells, reduced the proportion of regulatory T cells, and promoted the transition of macrophages from an M2 to an M1 phenotype. These changes effectively reshaped the tumor microenvironment from an immunosuppressive “cold” state to an immunologically active “hot” state, significantly enhancing the anti‐tumor immune response. Our research results showed that, by virtue of its excellent GSDME gene reactivation and chromatin loosening abilities, **HRP** effectively boosted tumor cell pyroptosis and exhibited the most pronounced anticancer performance. Furthermore, as a potential clinical therapeutic agent to treat TNBC, **HRP** provides a new approach for developing chemotherapy drugs with immunotherapeutic properties for the treatment of tumor cells with low GSDME expression. To the best of our knowledge, this is the first report of platinum complexes inducing pyroptosis via the Caspase‐3/GSDME pathway in tumor cells with low GSDME expression.

## Conflict of Interest

The authors declare no conflict of interest.

## Supporting information



Supporting Information

## Data Availability

The data that support the findings of this study are available from the corresponding author upon reasonable request.
