# Supplementary material for: Pyroptosis‐Inducing Platinum(IV) Prodrugs via GSDME Pathway for Chemoimmunotherapy and Metastasis Inhibition in Triple‐Negative Breast Cancer
Source: Adv Sci (Weinh). 2025 May 28;12(29):e05567. doi: 10.1002/advs.202505567 (PMC12362741; doi:10.1002/advs.202505567)
Supplement: Supplementary file 1 — Supporting Information [file ADVS-12-e05567-s001.docx]

Supporting Information

Pyroptosis-Inducing Platinum(IV) Prodrugs via GSDME Pathway for Chemoimmunotherapy and Metastasis Inhibition in Triple-Negative Breast Cancer

Xinda Yang^a†^, Chuansheng Xu^a†^, Youliang Zeng^b^, Chunhui Wang^a^, Yan Gao^a^, Jie Ding^a^, Sirui Chen^a^, Yuheng Pan^a^, Xin Zhang^a^, Zongwan Mao^b^*, Shuo Shi^a^*

^a^School of Chemical Science and Engineering, Department of Laboratory Medicine, Shanghai Tenth People’s Hospital of Tongji University, Tongji University, Shanghai 200092, P. R. China

^b^MOE Key Laboratory of Bioinorganic and Synthetic Chemistry, Guangdong Basic Research Center of Excellence for Functional Molecular Engineering, GBRCE for Functional Molecular Engineering, School of Chemistry, Sun Yat-Sen University, Guangzhou 510275, P. R. China

*Corresponding authors

E-mail addresses: Shuo Shi: shishuo@tongji.edu.cn,

Zongwan Mao: cesmzw@mail.sysu.edu.cn

^†^These authors contributed equally

.

**Table of Contents**

[Supplementary Tables and Figures 4](#_Toc198038598)

[Partition coefficients (Table S1). 4](#_Toc198038599)

[In vitro cytotoxicity data (Table S2). 4](#_Toc198038600)

[Primer sequence (Table S3). 4](#_Toc198038601)

[Characterization of RG108 (S1-S3). 5](#_Toc198038602)

[Characterization of RG108-NHS (S4-S6). 6](#_Toc198038603)

[Characterization of MRP (S7-S10). 8](#_Toc198038604)

[Characterization of DRP (S11-S14). 10](#_Toc198038605)

[Characterization of HRP (S15-S18). 12](#_Toc198038606)

[Results of Stability and Reducibility (Figures S19-S22). 14](#_Toc198038607)

[Stability of Pt^IV^ complexes in cells (Figures S23-S25). 16](#_Toc198038608)

[¹⁹⁵Pt NMR spectra of degradation experiments (Figures S26-S28). 17](#_Toc198038609)

[Binding assay of MRP, DRP and HRP with 5'-GMP (Figures S29-S31). 18](#_Toc198038610)

[Results of Transcriptomic analysis (Figure S32-39) 21](#_Toc198038611)

[Statistical count of cells undergoing pyroptosis (Figure S40). 25](#_Toc198038612)

[WB results of Caspase-3 induced by CDDP (Figure S41). 25](#_Toc198038613)

[WB results of GSDME induced by RG108 (Figure S42). 26](#_Toc198038614)

[qPCR statistical analysis of IL-18 and IL-β (Figure S43). 26](#_Toc198038615)

[Tunnel positive cell counts (Figure S44). 27](#_Toc198038616)

[Immunofluorescence staining of HMGB1 (Figure S45). 27](#_Toc198038617)

[H&E-stained images of heart tissues (Figure S46). 28](#_Toc198038618)

[H&E-stained images of kidney tissues (Figure S47). 29](#_Toc198038619)

[Expression levels of UREA and CREA (Figure S48). 30](#_Toc198038620)

[The gating strategy for the Flow cytometry analysis (Figures S49-51). 30](#_Toc198038621)

[Methods 32](#_Toc198038622)

[1 Materials 32](#_Toc198038623)

[2 Cell Lines 33](#_Toc198038624)

[3 Animals 33](#_Toc198038625)

[4 General Methods 33](#_Toc198038626)

[5 Synthesis and characterization 33](#_Toc198038627)

[5.1 Synthesis of RG108 33](#_Toc198038628)

[5.2 Synthesis of RG108-NHS 34](#_Toc198038629)

[5.3 Synthesis of Oxoplatin 35](#_Toc198038630)

[5.4 Synthesis of MRP 35](#_Toc198038631)

[5.5 Synthesis of DRP 35](#_Toc198038632)

[5.6 Synthesis of the HRP 36](#_Toc198038633)

[7 Measurement of Partition Coefficient 37](#_Toc198038634)

[8 Stability and Reducibility Degradation of Pt^IV^ Complexes 37](#_Toc198038635)

[9 Cell viability assay 38](#_Toc198038636)

[11 In vitro Clonogenic Assay 39](#_Toc198038637)

[12 In vitro 2D Cell Migration Assay 39](#_Toc198038638)

[13 Tumor spheroid 3D migration assay 40](#_Toc198038639)

[14 Live/dead cell staining assay 40](#_Toc198038640)

[16 Intracellular Accumulation 41](#_Toc198038641)

[17 DNA-binding Assay 41](#_Toc198038642)

[18 mRNA sequencing experimental 42](#_Toc198038643)

[18.1 RNA Isolation and Library Preparation 42](#_Toc198038644)

[18.2 RNA Sequencing and Differentially Expressed Genes Analysis 42](#_Toc198038645)

[19 Western blot assays 43](#_Toc198038646)

[20 qPCR Experiment 43](#_Toc198038647)

[21 IL-18 and IL-1β detection 44](#_Toc198038648)

[22 Flow cytometry assay of immune cells population 44](#_Toc198038649)

[23 In Vivo Antitumor Study 45](#_Toc198038650)

[24 TNF-α, IL-6, IL-12 and IFN-γ detection 45](#_Toc198038651)

[25 Statistical Analysis 46](#_Toc198038652)

[Reference 46](#_Toc198038653)

# Supplementary Tables and Figures

Partition coefficients (Table S1).

| **Table S1.** Partition coefficients of CDDP, MRP, DRP and HRP. | | | | |
| --- | --- | --- | --- | --- |
| **Compounds** | **CDDP** | **MRP** | **DRP** | **HRP** |
| log*P*_O/W_ | -2.33 ± 0.30 | 0.95 ± 0.15 | 1.65 ± 0.20 | 1.76 ± 0.15 |

In vitro cytotoxicity data (Table S2).

| **Table S2** IC_50_ (μM) of CDDP, RG108, MRP, DRP and HRP at 48 h against 4T1 cells with or without Z-DEVD-FMK. | | | | | |
| --- | --- | --- | --- | --- | --- |
| **Complexes** | **CDDP** | **RG108** | **MRP** | **DRP** | **HRP** |
| Vehicle | 16.68 ± 1.95 | ﹥125 | 10.49 ± 0.90 | 5.64 ± 0.65 | 1.99 ± 0.16 |
| Z-DEVD-FMK | 22.67 ± 2.44 | ﹥125 | 19.25 ± 1.75 | 18.18 ± 2.73 | 12.37 ± 1.52 |

Primer sequence (Table S3).

| **Table S3.** Primer sequence. | | |
| --- | --- | --- |
| **Genes** | **Types of Primers** | **Sequence** |
| **β-actin** | Forward | ACCTTCTACAATGAGCTGCG |
|  | Reverse | CTGGATGGCTACGTACATGG |
| **IL-1b** | Forward | TTCAGGCAGGCAGTATCACTC |
|  | Reverse | GAAGGTCCACGGGAAAGACAC |
| **IL-18** | Forward | AGACTACTTCCTGAGCACAAGA |
|  | Reverse | TGTCCTTACCAATGGTTCTCACT |
| **Caspase-3** | Forward | TGGTGATGAAGGGGTCATTTATG |
|  | Reverse | TTCGGCTTTCCAGTCAGACTC |
| **GSDME** | Forward | TGCAACTTCTAAGTCTGGTGACC |
|  | Reverse | CTCCACAACCACTGGACTGAG |

Characterization of RG108 (S1-S3).


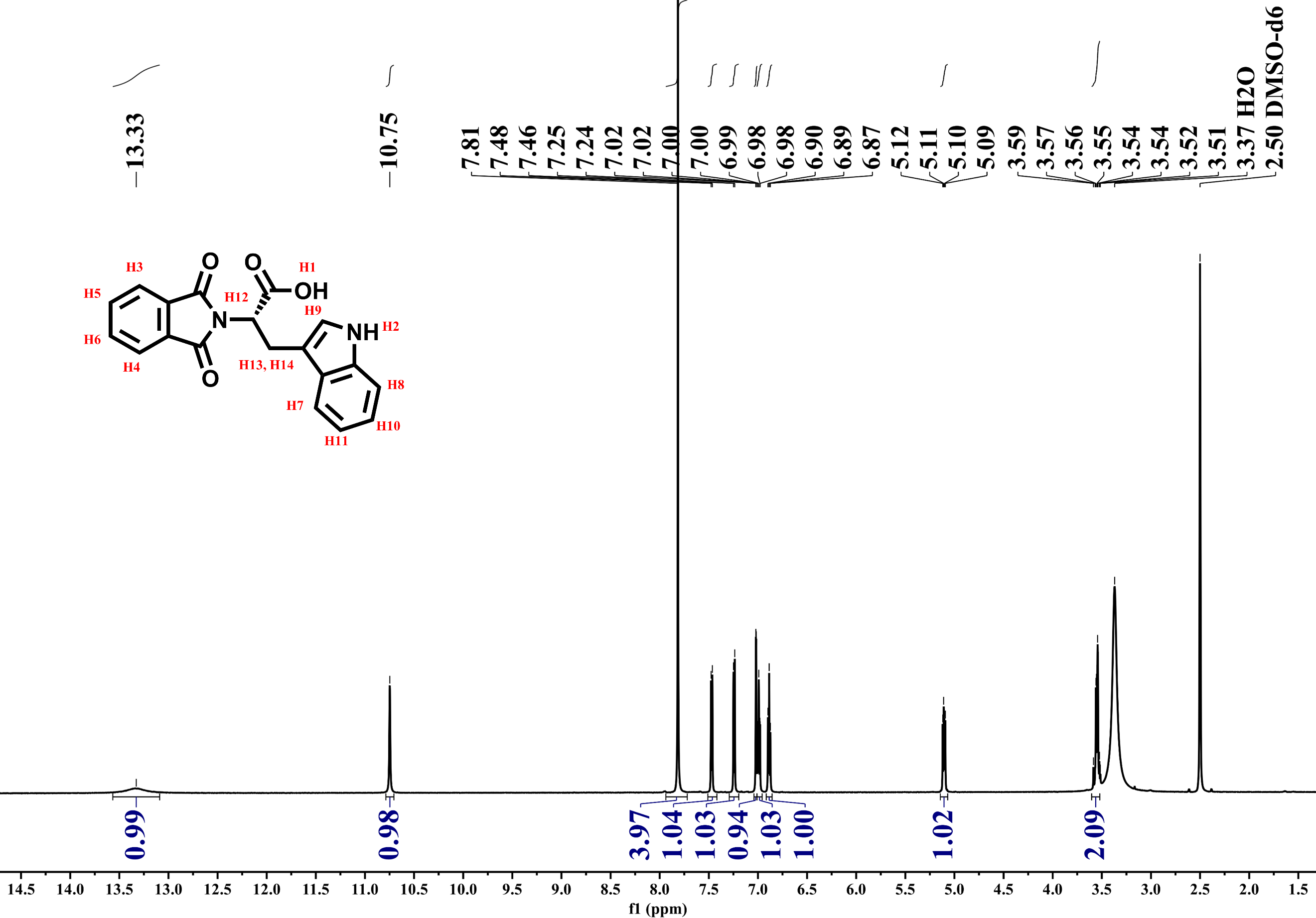


**Figure S1.** ^1^H-NMR spectrum of RG108 in DMSO-*d*_6_.


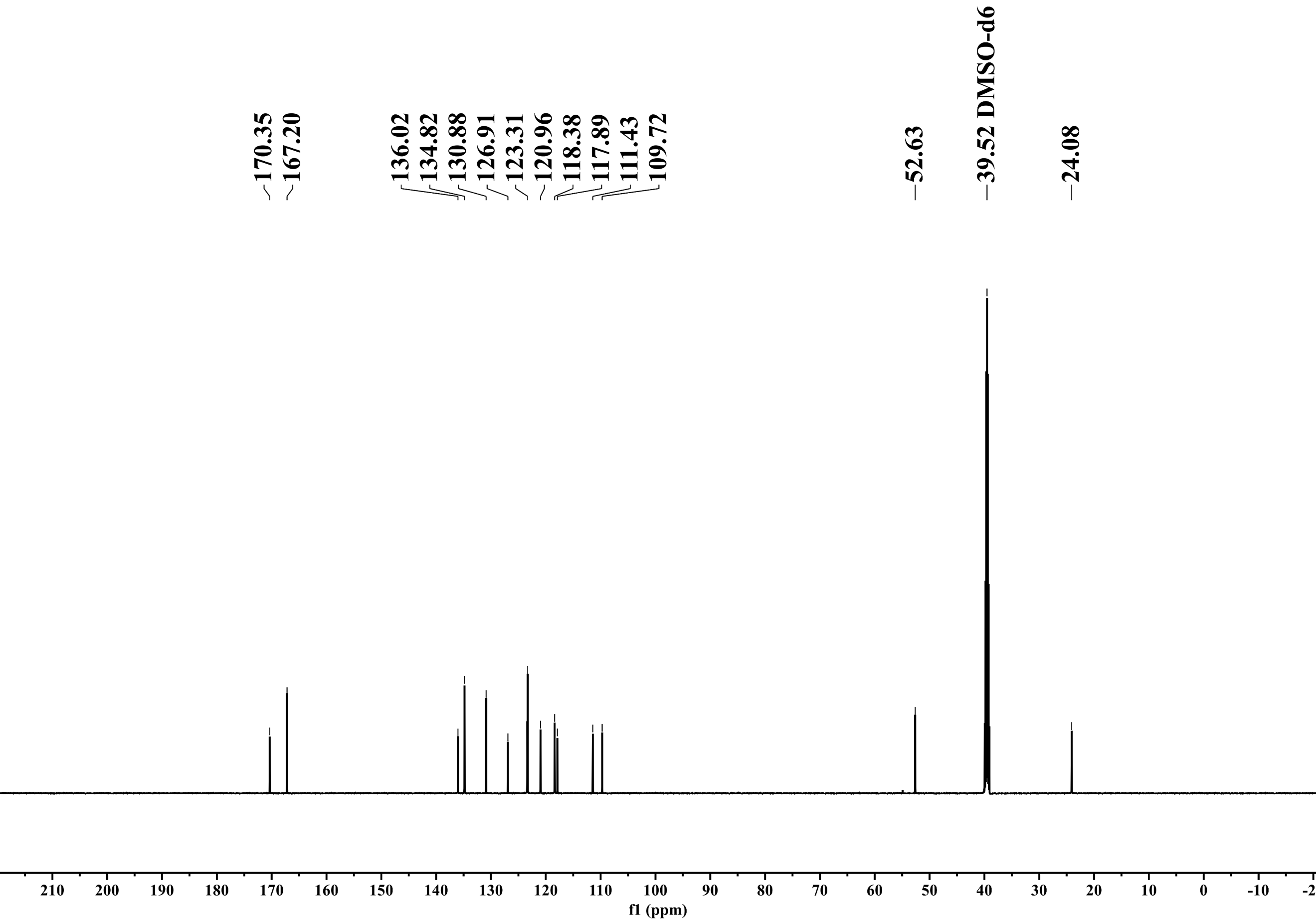


**Figure S2.** ^13^C-NMR spectrum of RG108 in DMSO-*d*_6_.


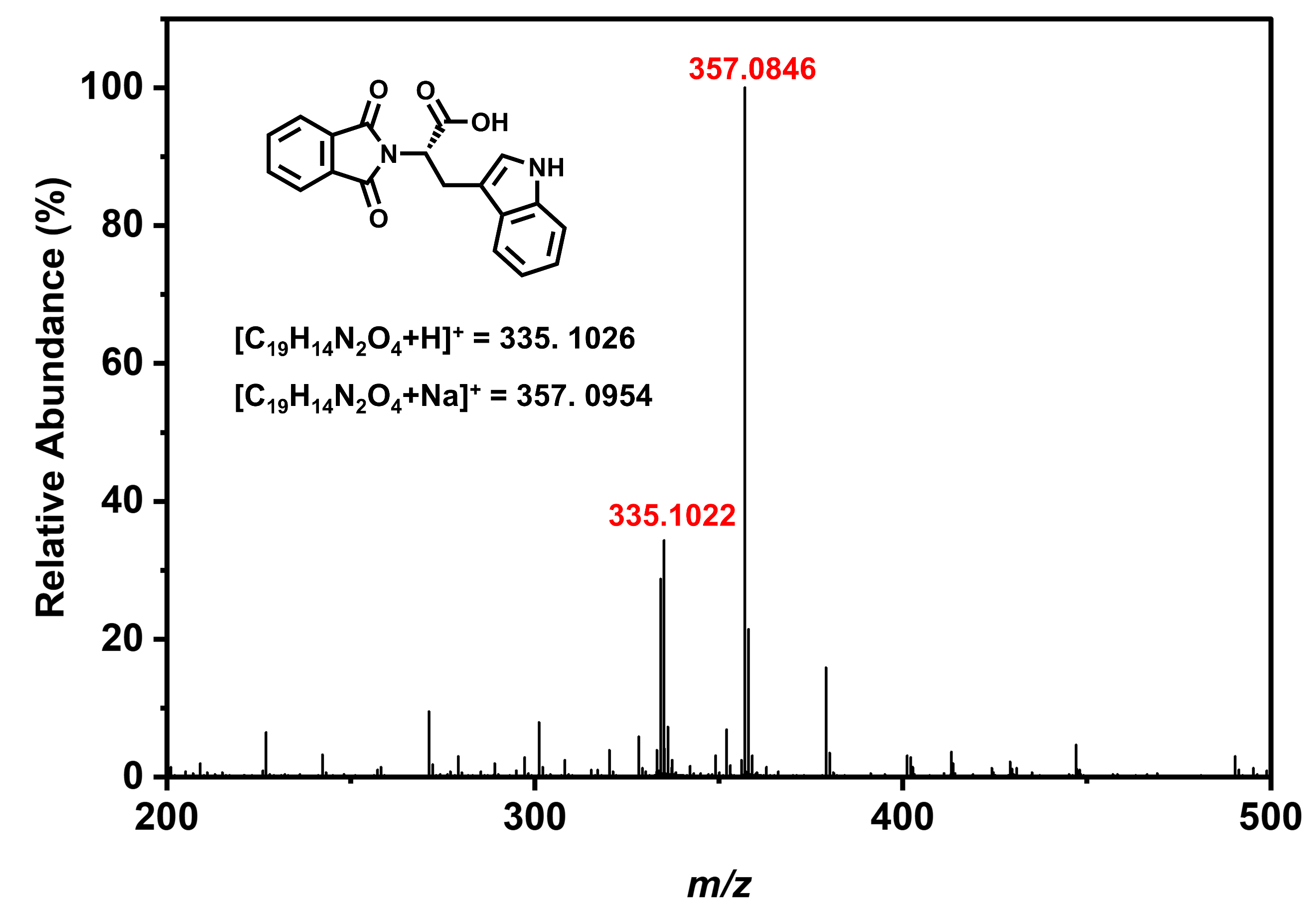


**Figure S3.** ESI-MS (positive mode) spectra of RG108 in methanol.

Characterization of RG108-NHS (S4-S6).


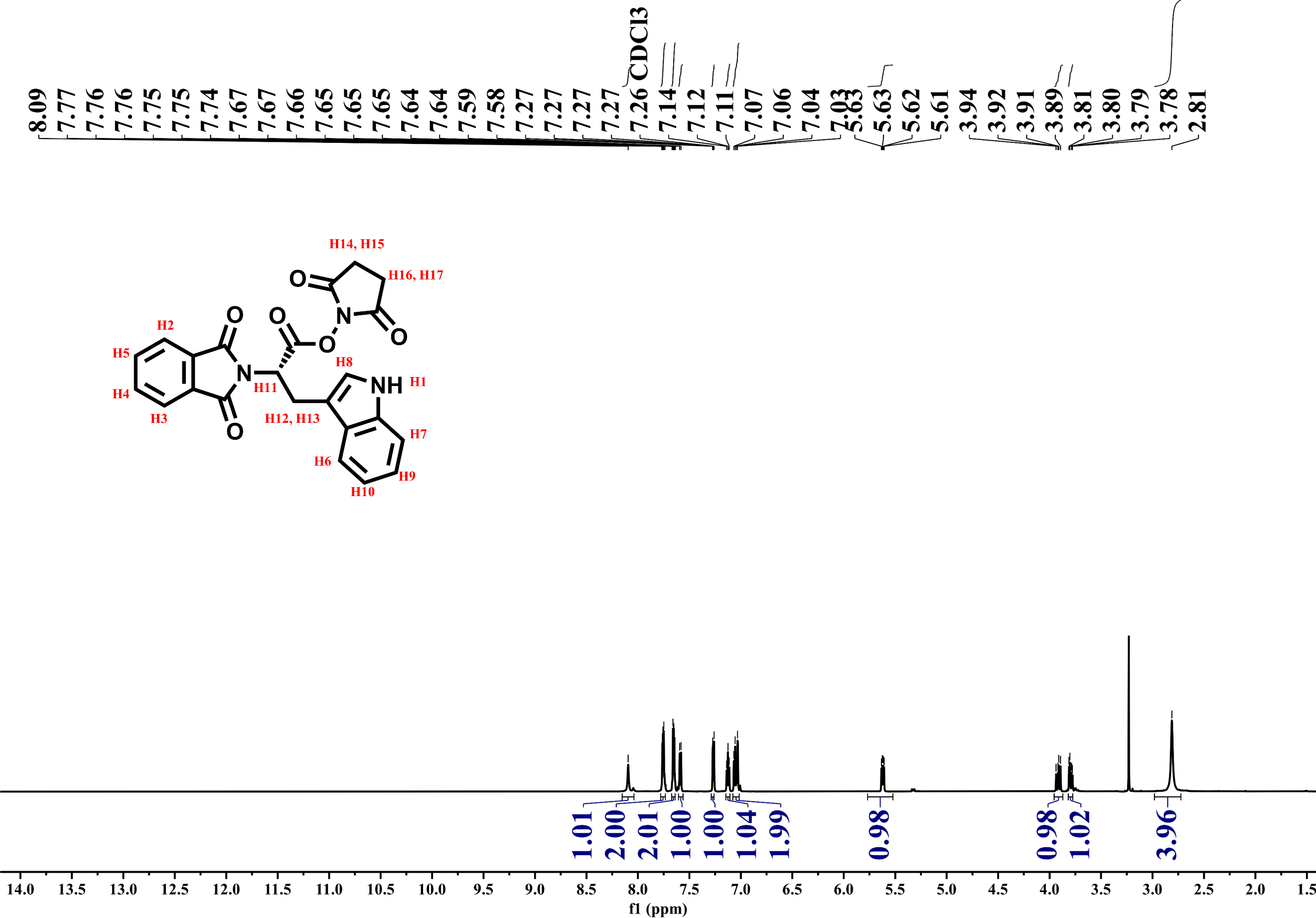


**Figure S4.** ^1^H-NMR spectrum of RG108-NHS in CDCl_3_.


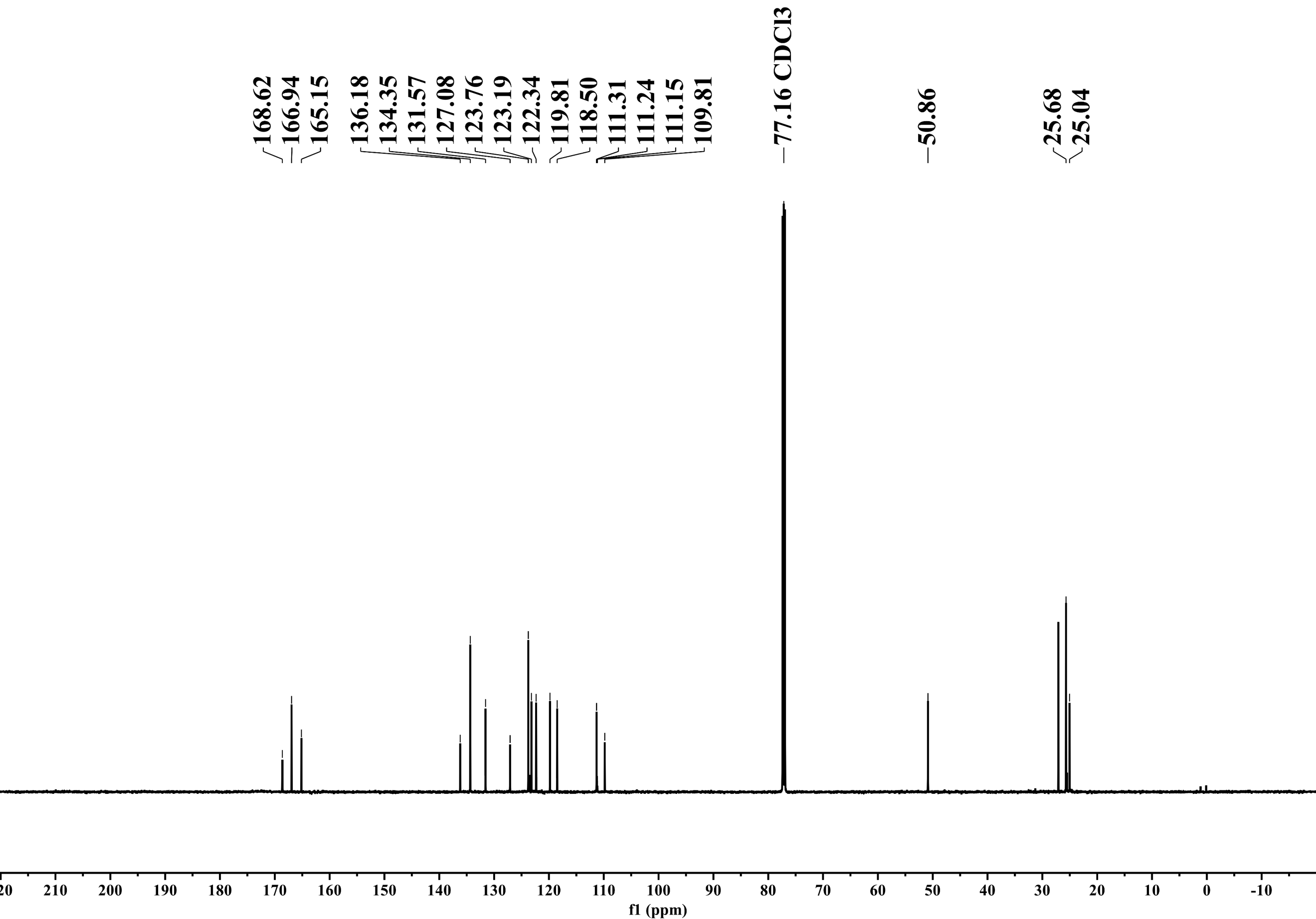


**Figure S5.** ^13^C-NMR spectrum of RG108-NHS in CDCl_3_.


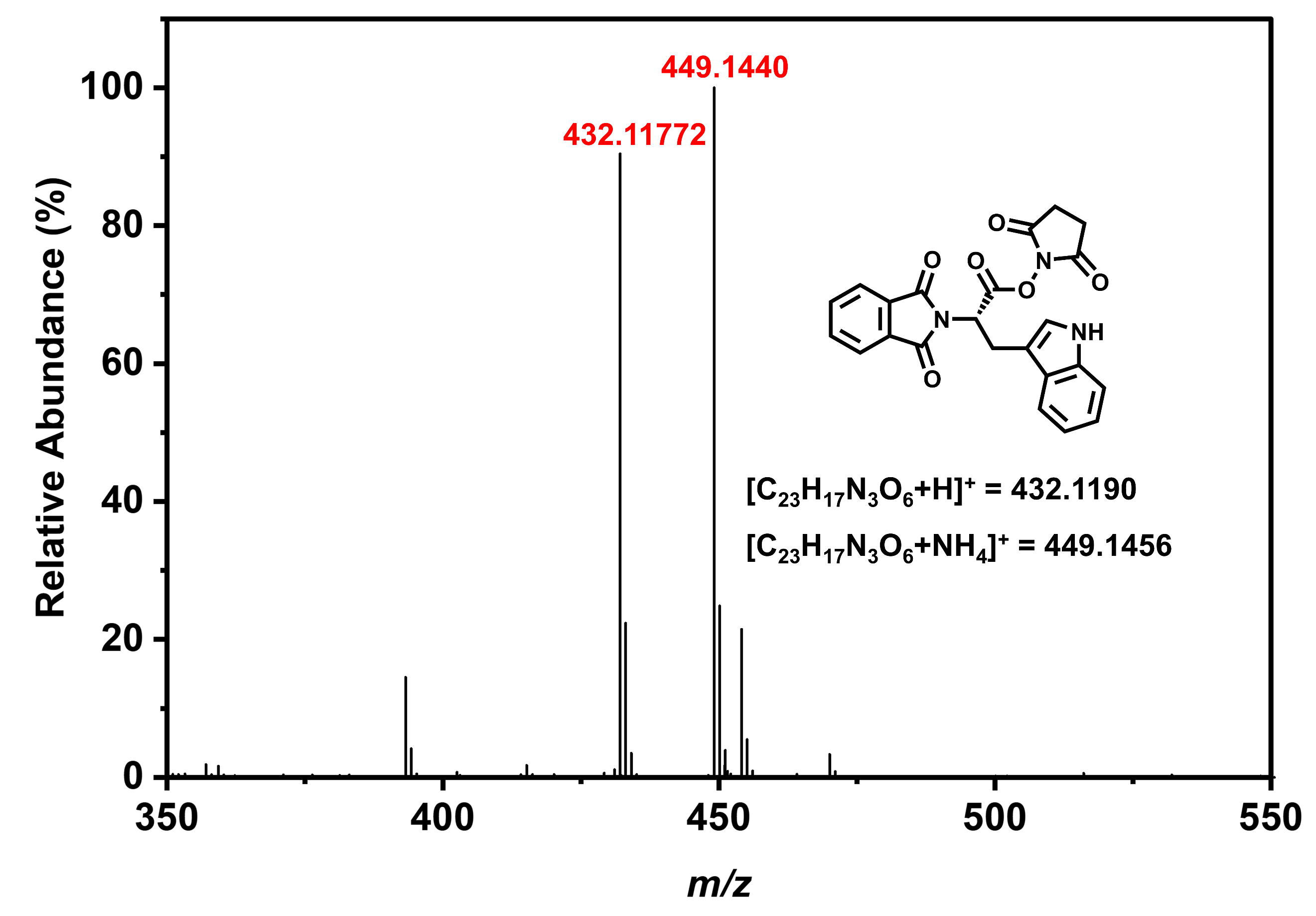


**Figure S6.** ESI-MS (positive mode) spectra of RG108-NHS in methanol.

Characterization of MRP (S7-S10).


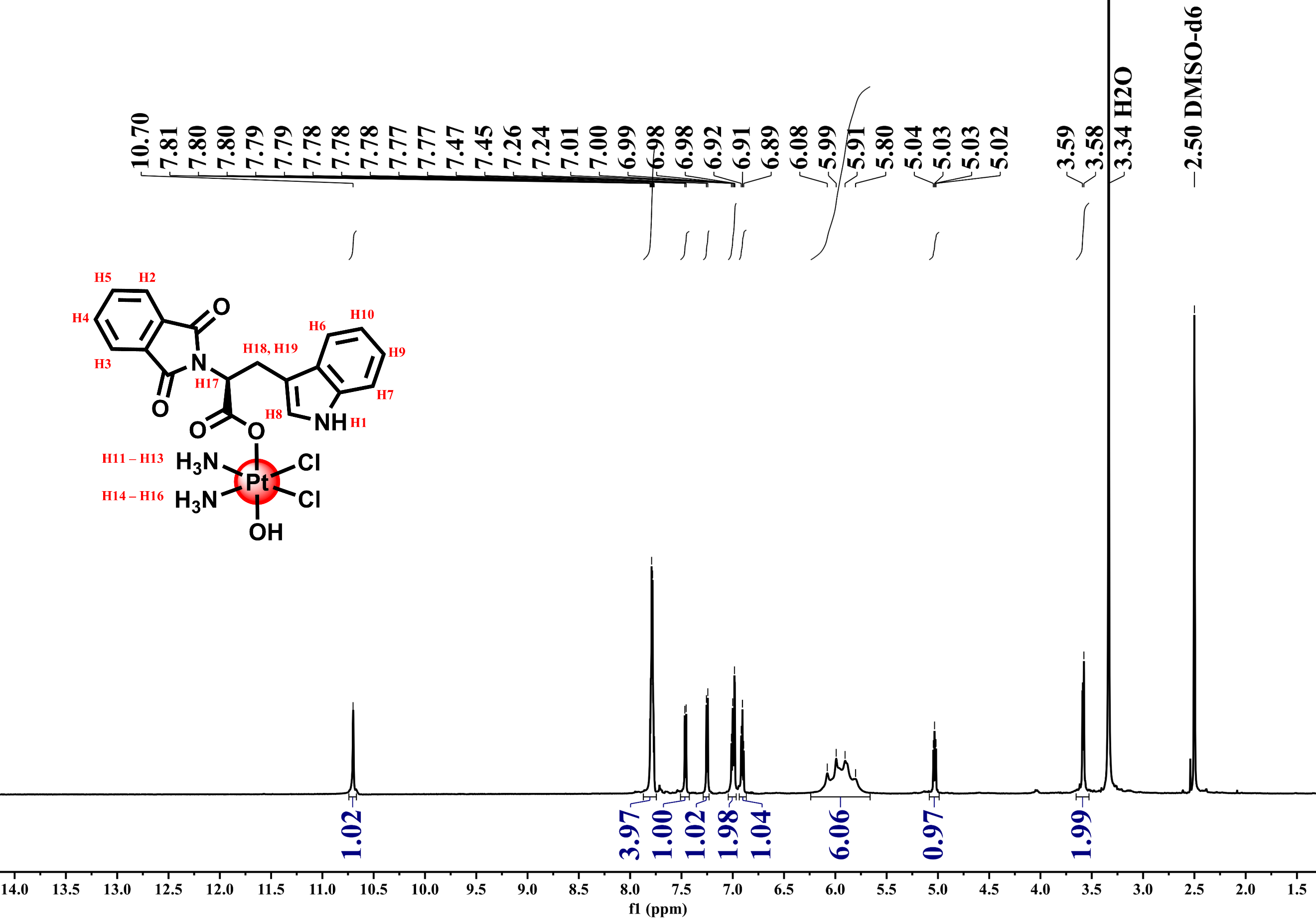

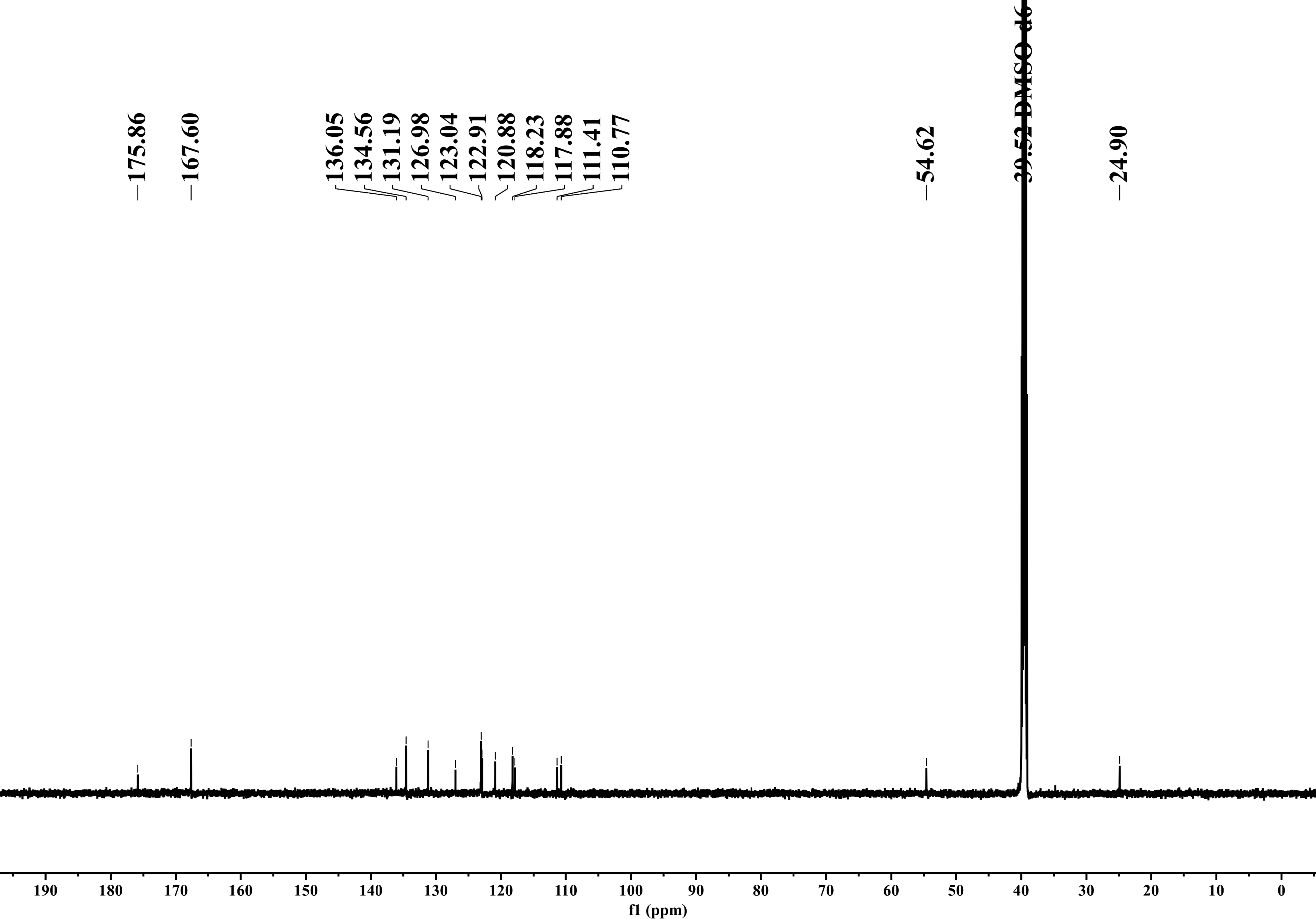


**Figure S7.** ^1^H-NMR spectrum of MRP in DMSO-*d*_6_.


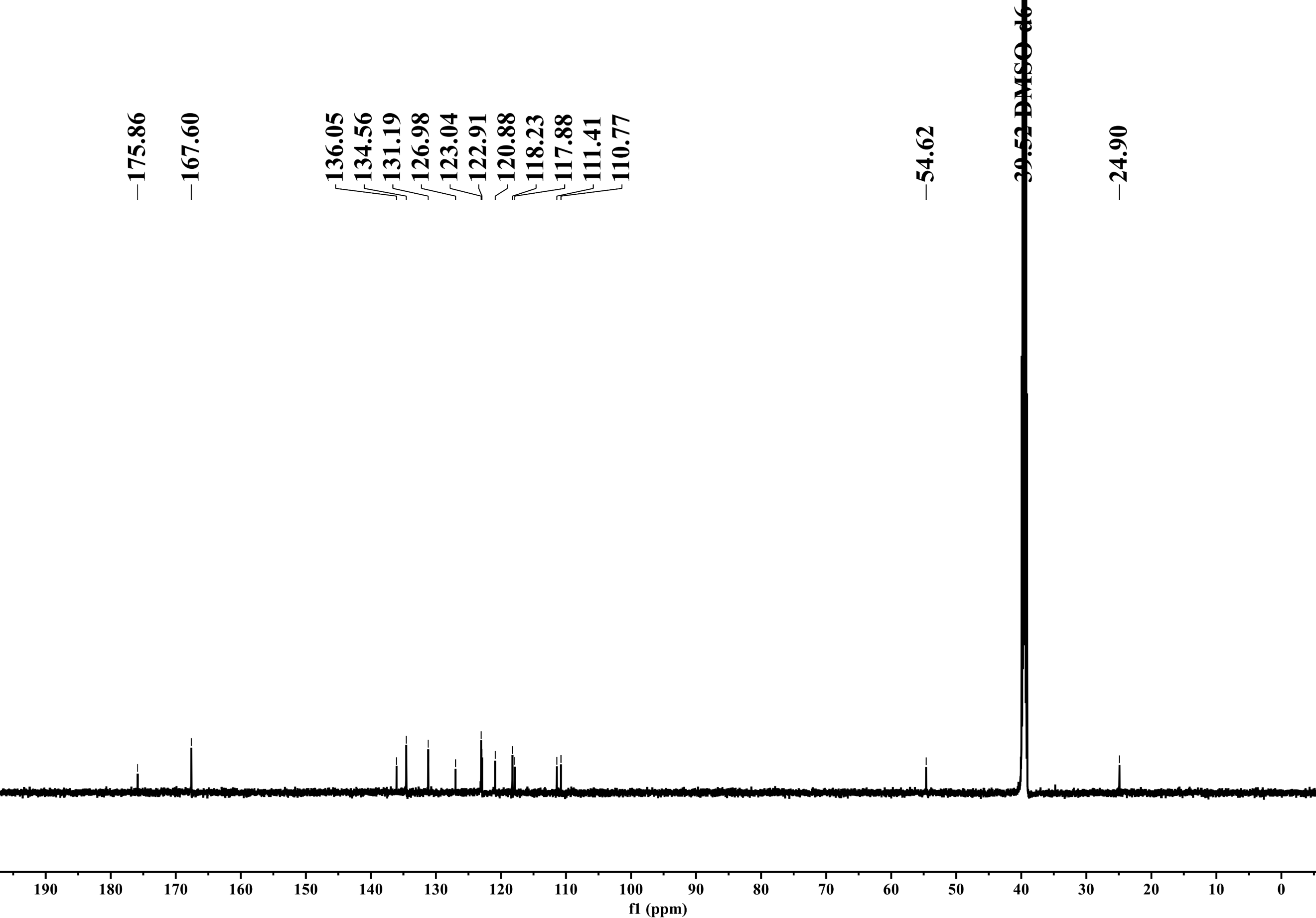


**Figure S8.** ^13^C-NMR spectrum of MRP in DMSO-*d*_6_.


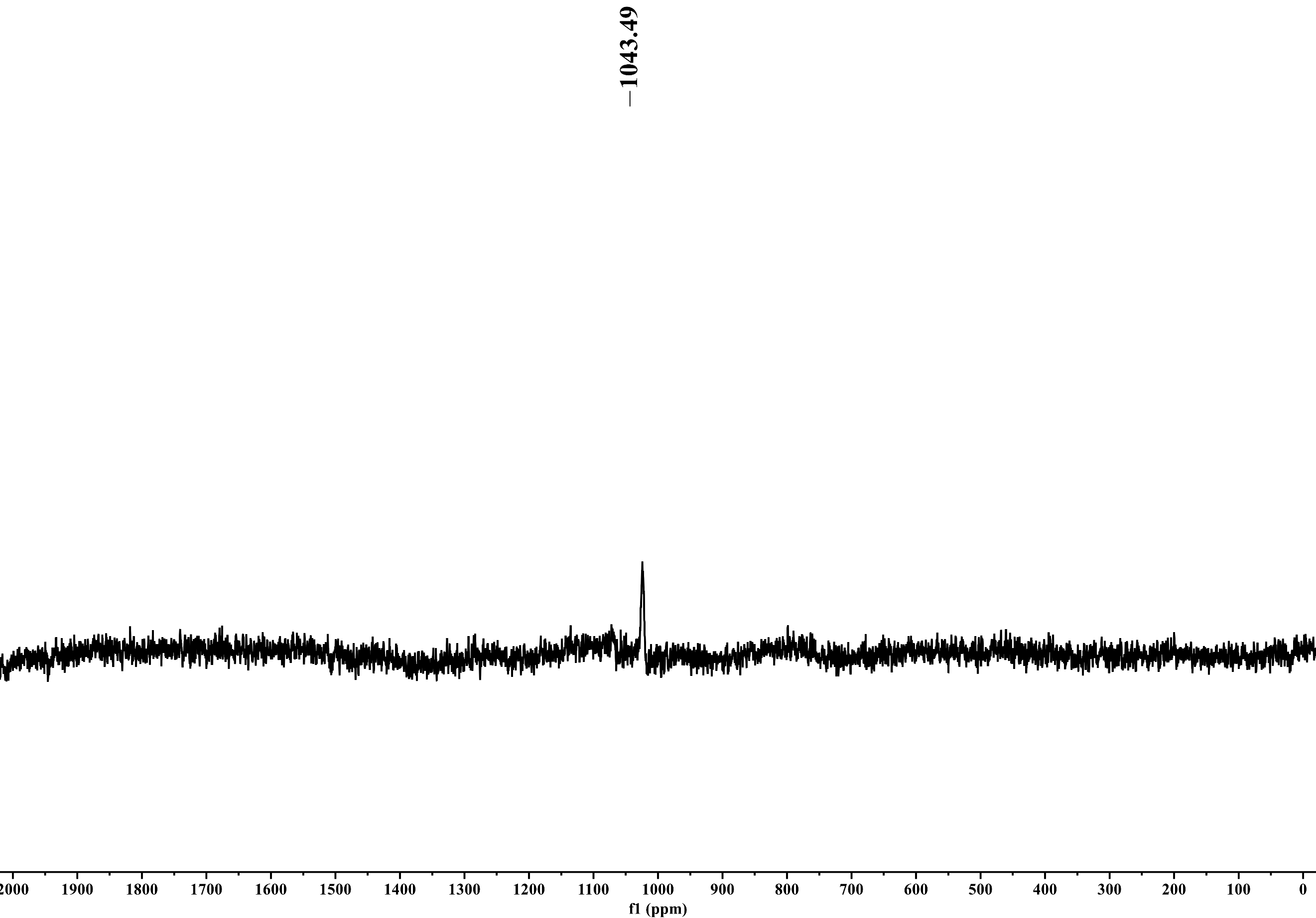


**Figure S9.** ^195^Pt-NMR spectrum of MRP in DMSO-*d*_6_.


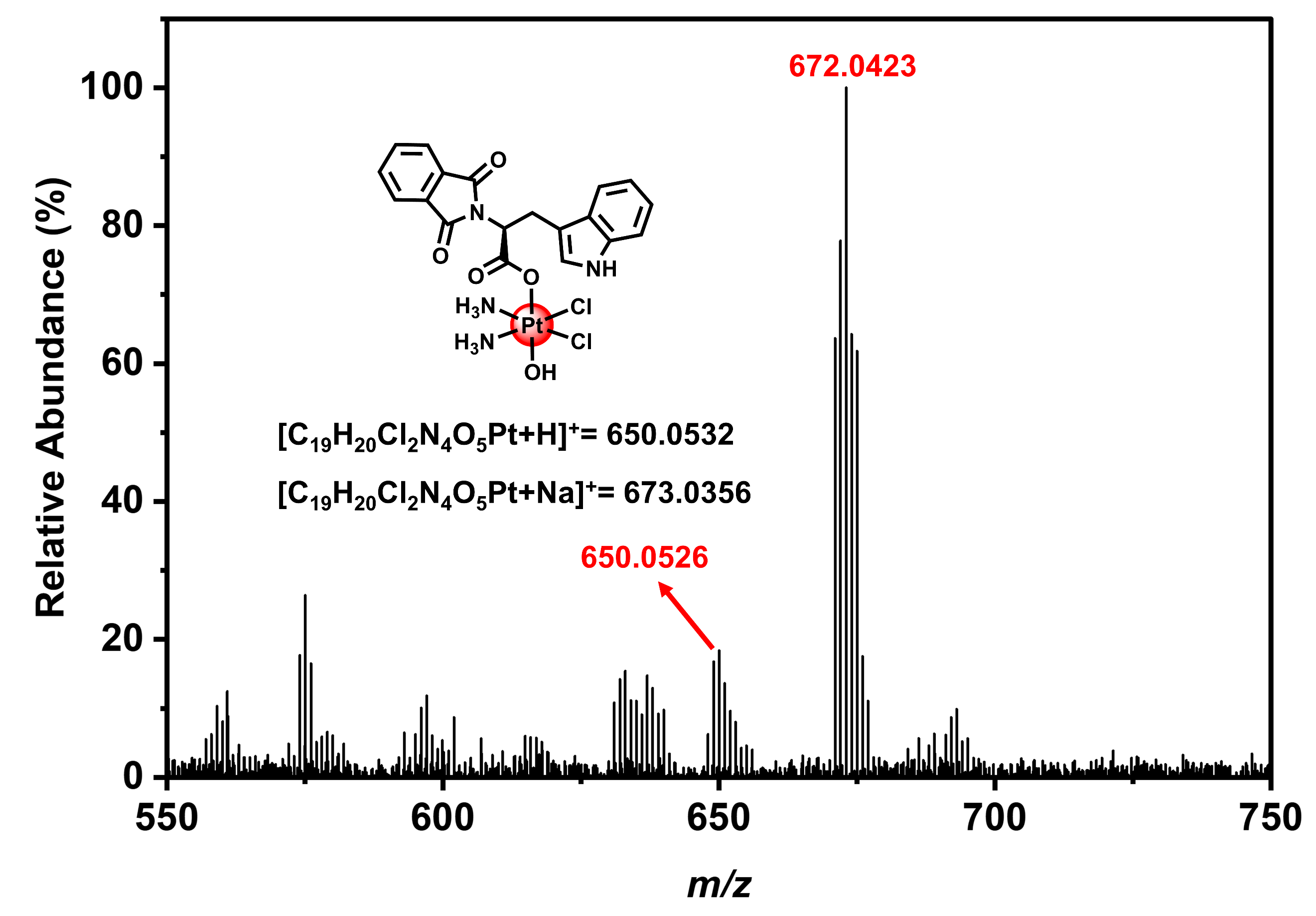


**Figure S10.** ESI-MS (positive mode) spectra of MRP in methanol.

Characterization of DRP (S11-S14).


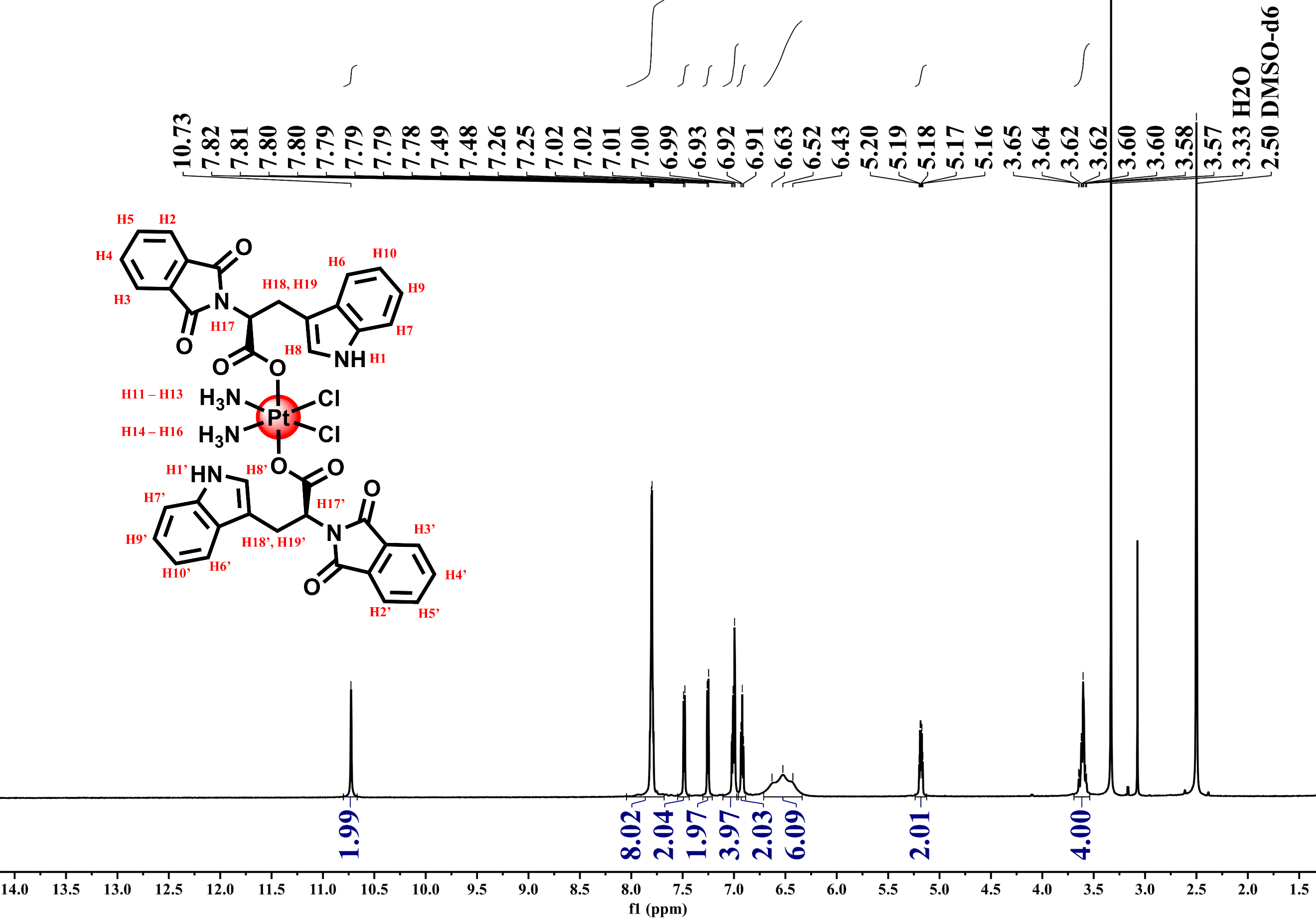


**Figure S11.** ^1^H-NMR spectrum of DRP in DMSO-*d*_6_.


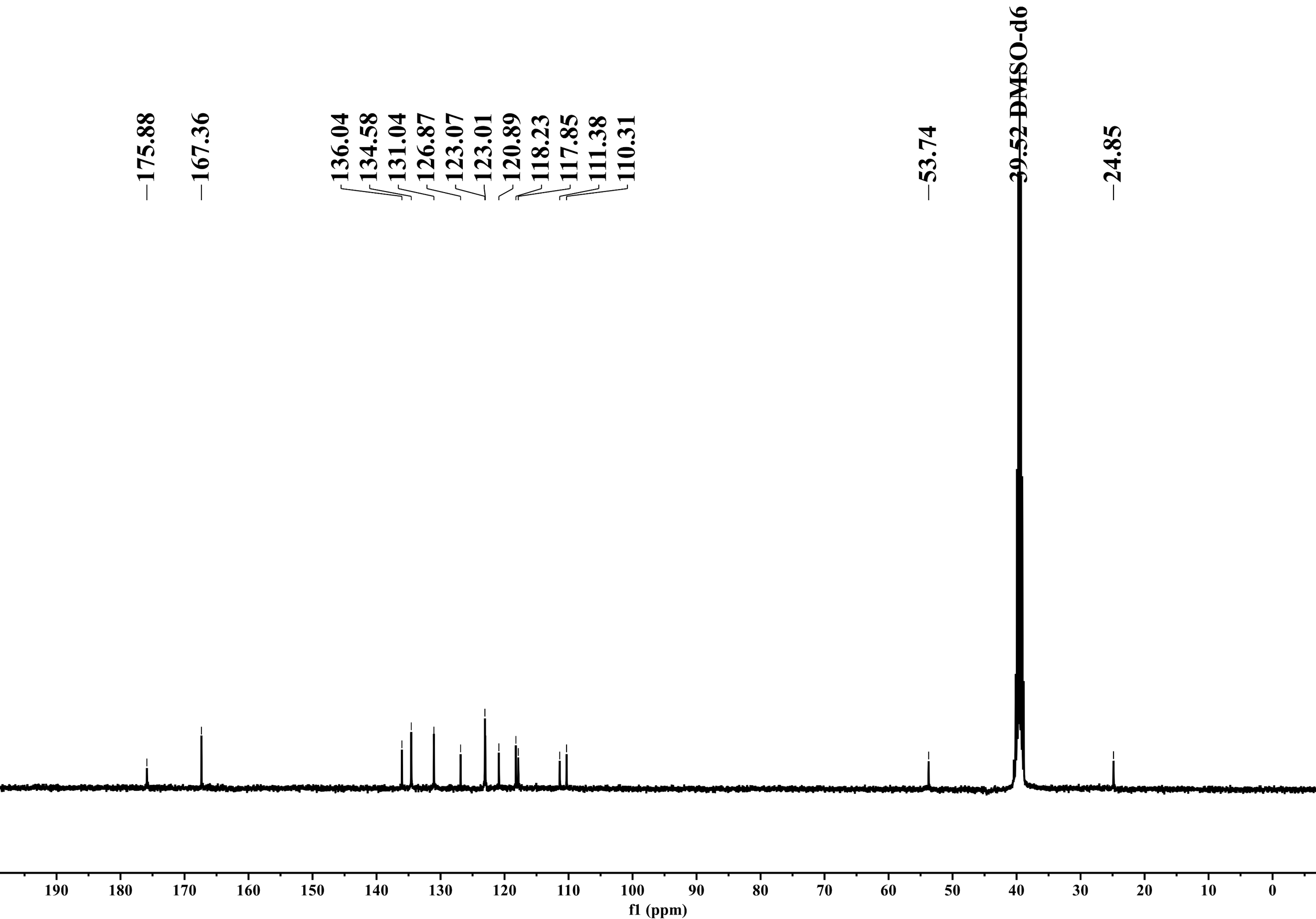


**Figure S12.** ^13^C-NMR spectrum of DRP in DMSO-*d*_6_.


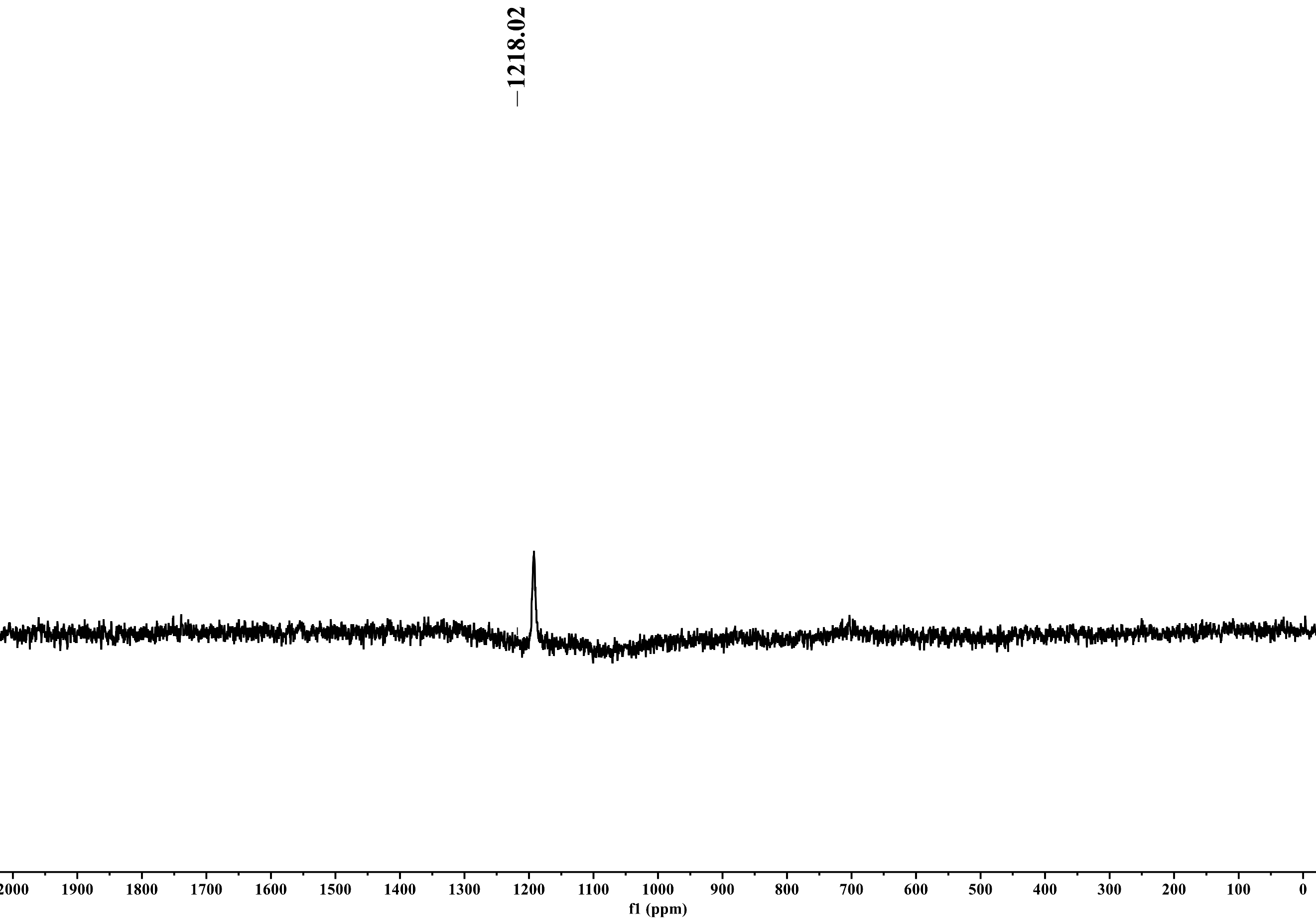


**Figure S13.** ^195^Pt-NMR spectrum of DRP in DMSO-*d*_6_.


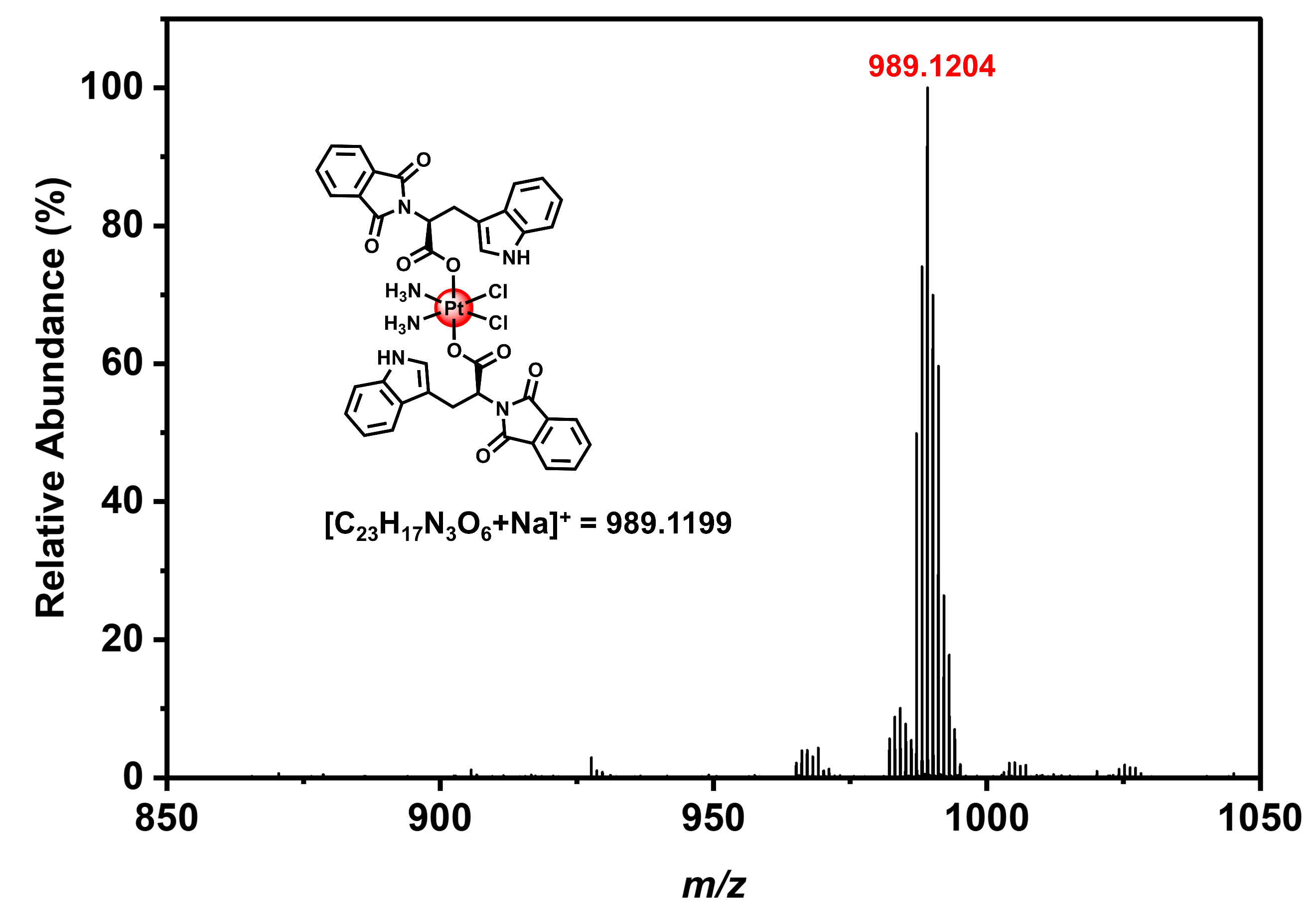


**Figure S14.** ESI-MS (positive mode) spectra of DRP in methanol.

Characterization of HRP (S15-S18).


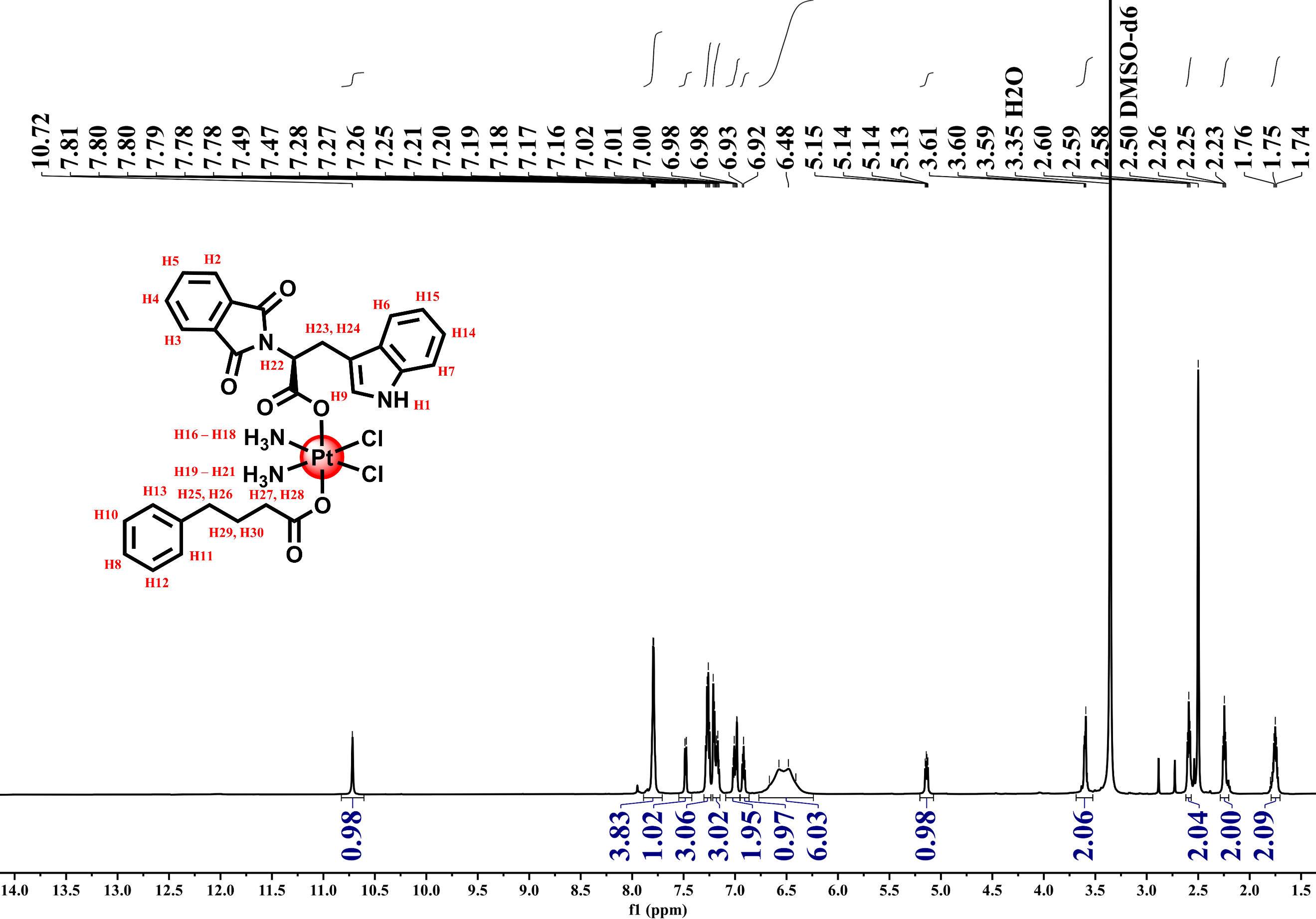


**Figure S15.** ^1^H-NMR spectrum of HRP in DMSO-*d*_6_.


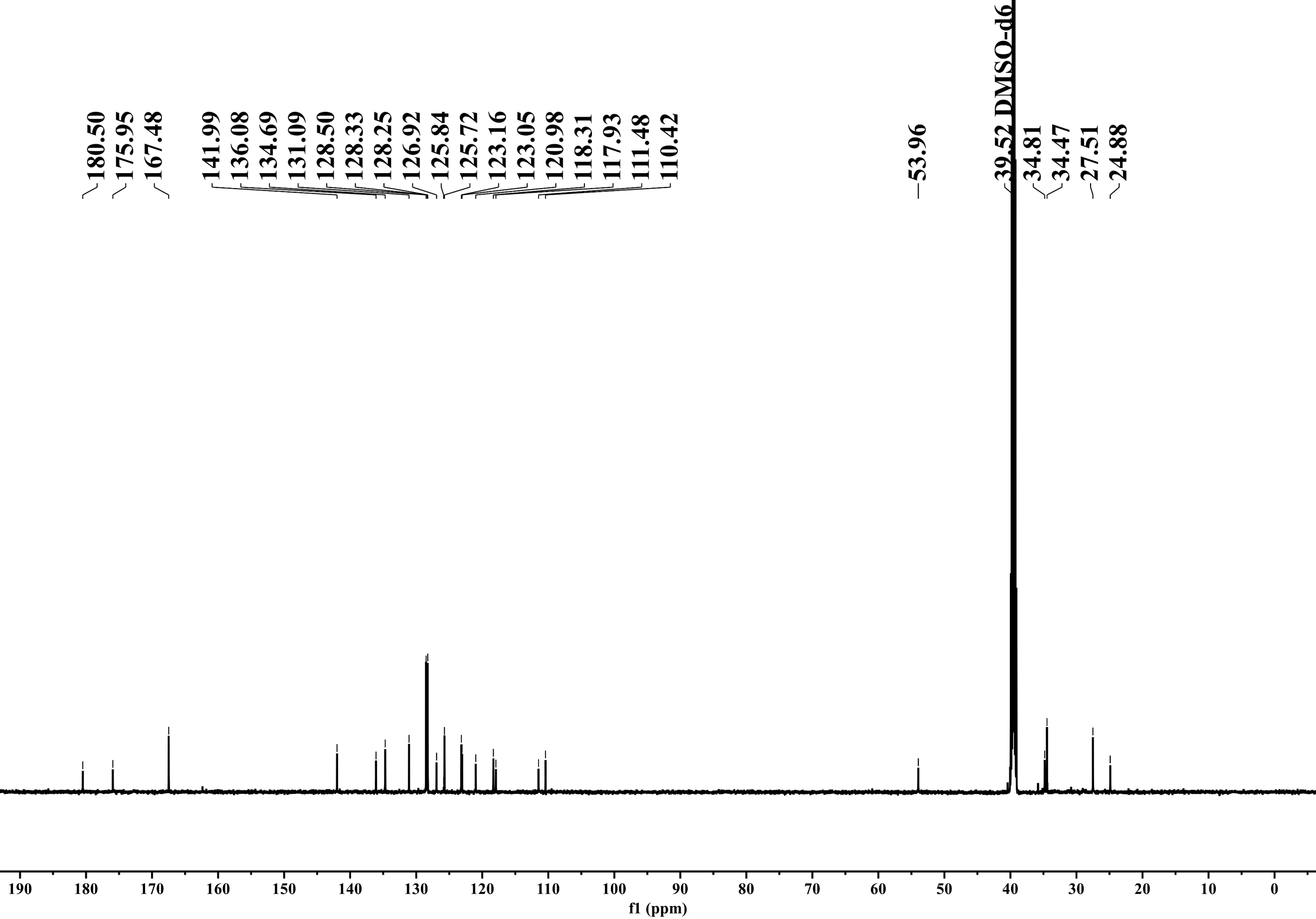


**Figure S16.** ^13^C-NMR spectrum of HRP in DMSO-*d*_6_.


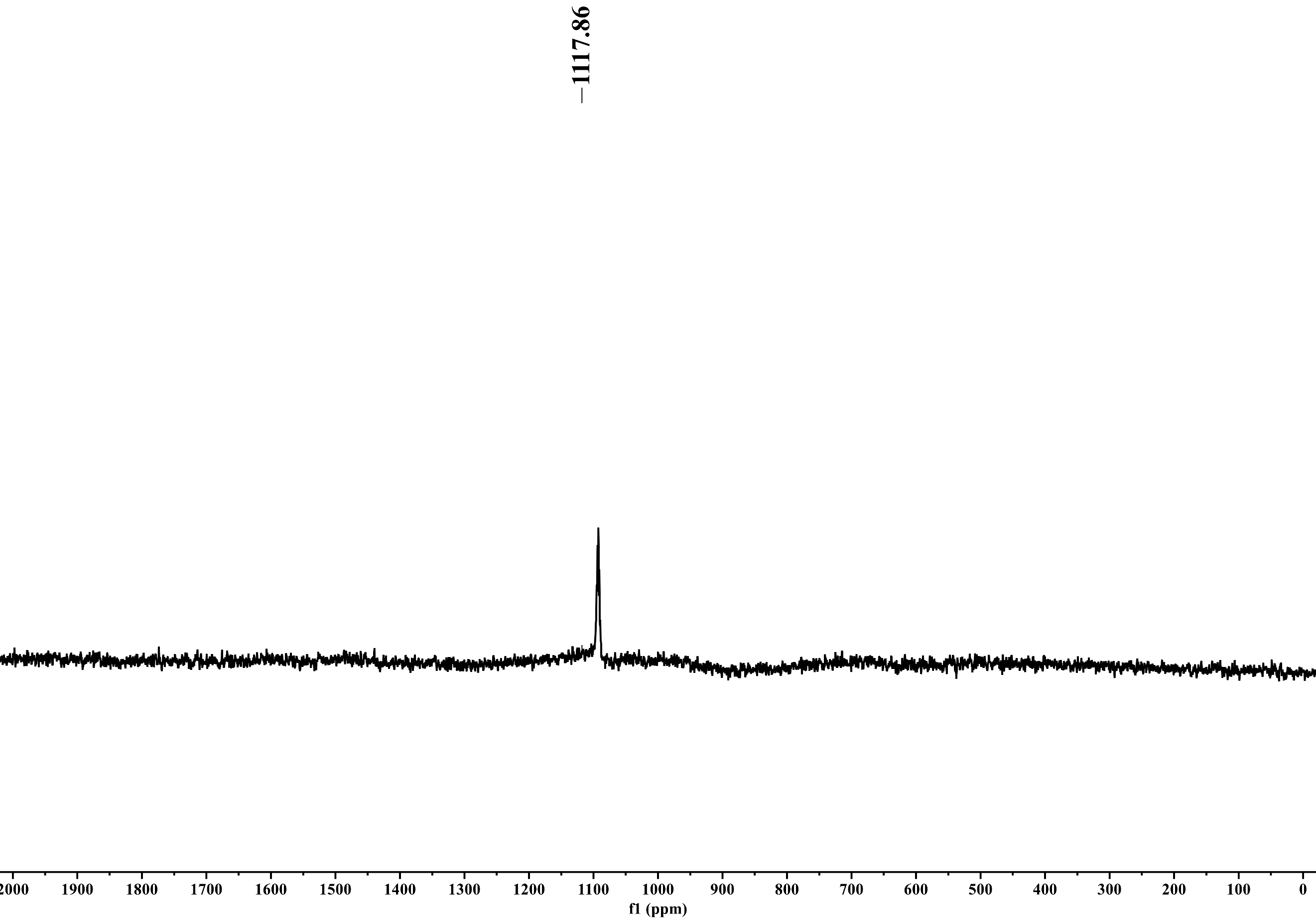


**Figure S17.** ^195^Pt-NMR spectrum of HRP in CD_3_OD.


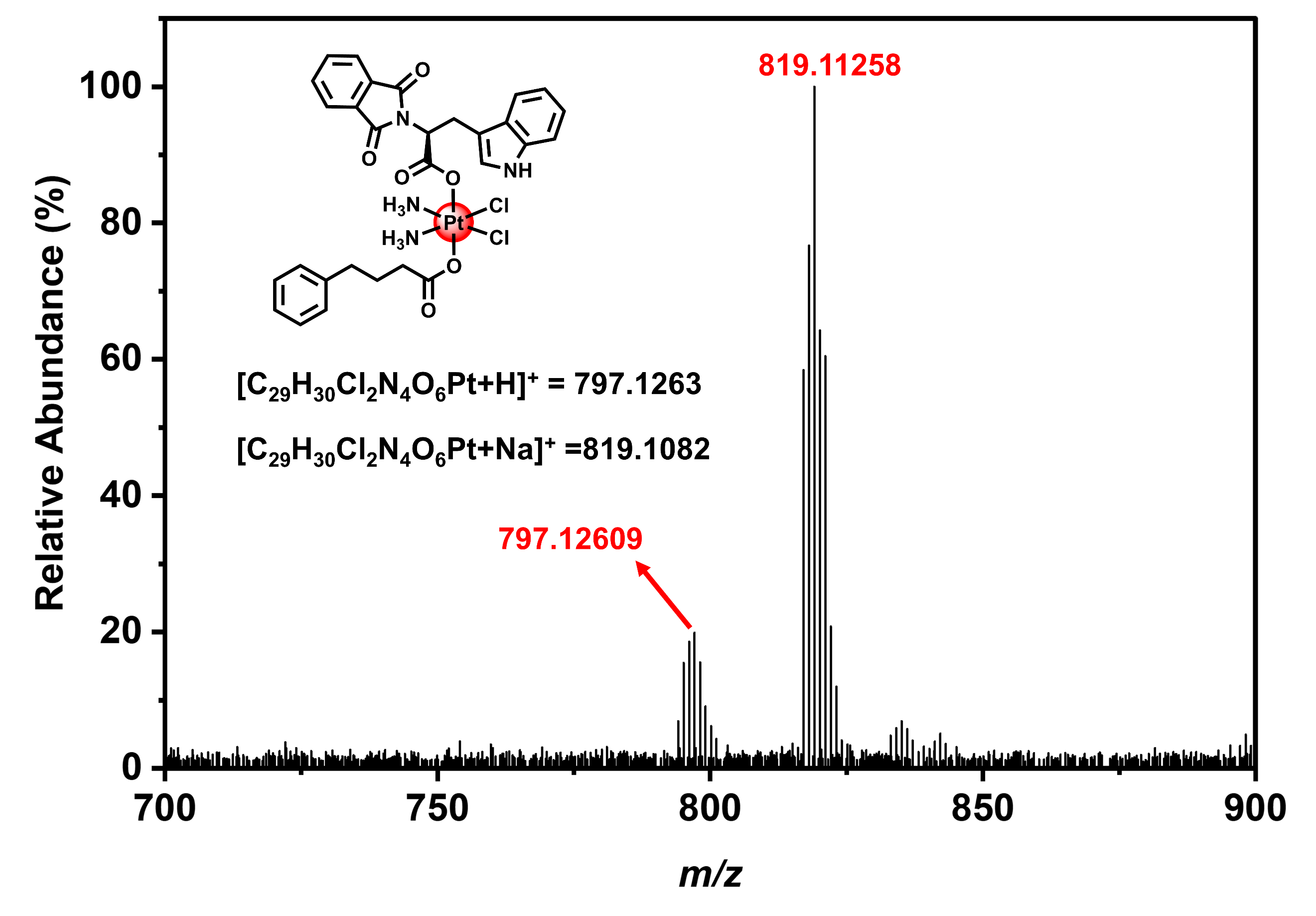


**Figure S18.** ESI-MS (positive mode) spectra of HRP in methanol.

Results of Stability and Reducibility (Figures S19-S22).


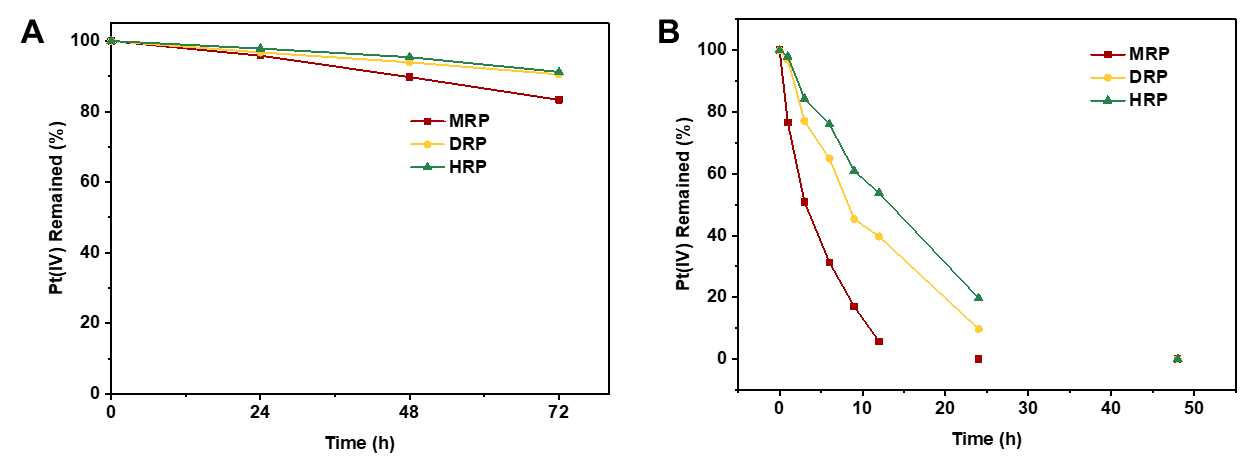


**Figure S19. (**A) Stability of Pt^IV^ complexes in PBS/MeOH (9/1, V/V, pH 7.4) at 37 °C in the dark; (B) Reduction of 50 μM Pt^IV^ complexes in 2 mM ASA in PBS/MeOH (9/1, V/V, pH 7.4) solution at 37 ºC in the dark.

**
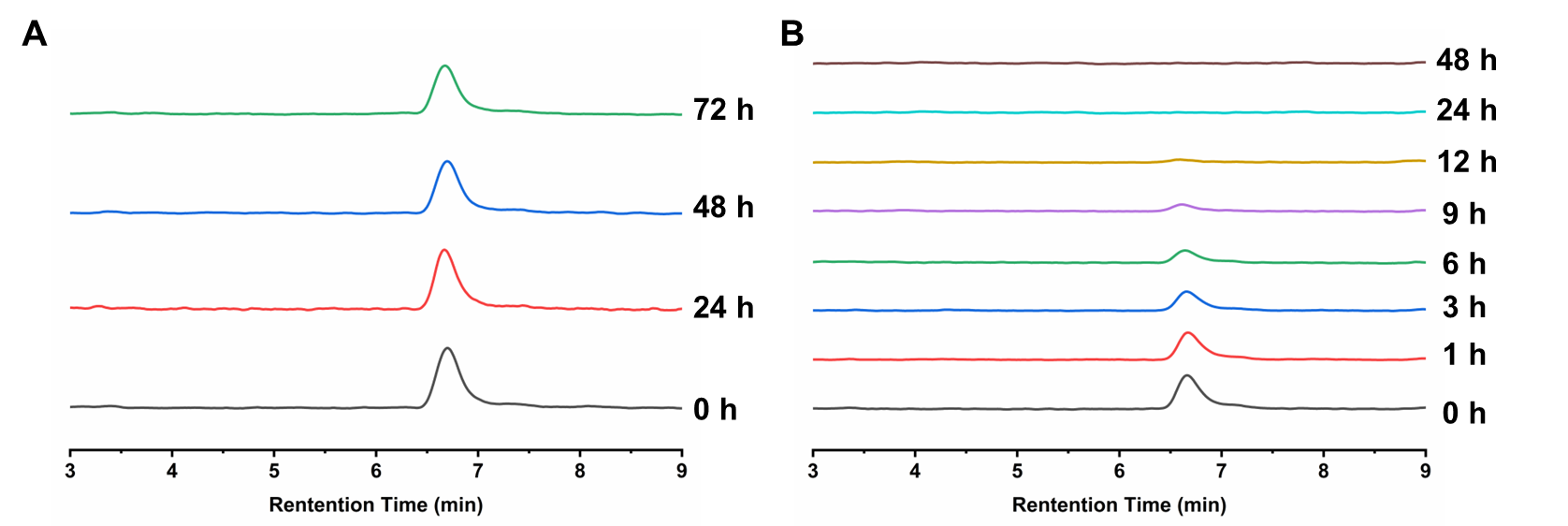
**

**Figure S20. (**A) Stability of MRP in PBS/MeOH (9/1, V/V, pH 7.4) tested by RP-HPLC at 37 °C in the dark; (B) Reduction of 50 μM MRP in 2 mM ascorbic acid in PBS/MeOH (9/1, V/V, pH 7.4) solution was tested by RP-HPLC at 37 ºC in the dark.

**
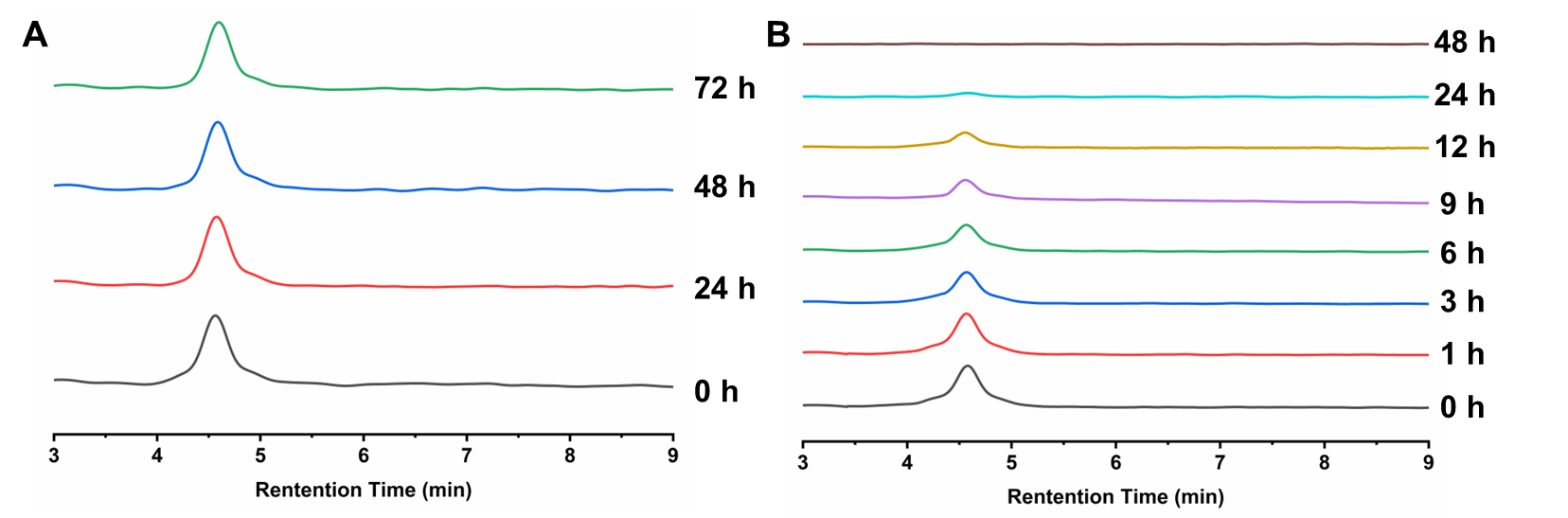
**

**Figure S21.** (A) Stability of DRP in PBS/MeOH (9/1, V/V, pH 7.4) tested by RP-HPLC at 37 °C in the dark; (B) Reduction of 50 μM HRP in 2 mM ascorbic acid in PBS/MeOH (9/1, V/V, pH 7.4) solution was tested by RP-HPLC at 37 ºC in the dark.

**
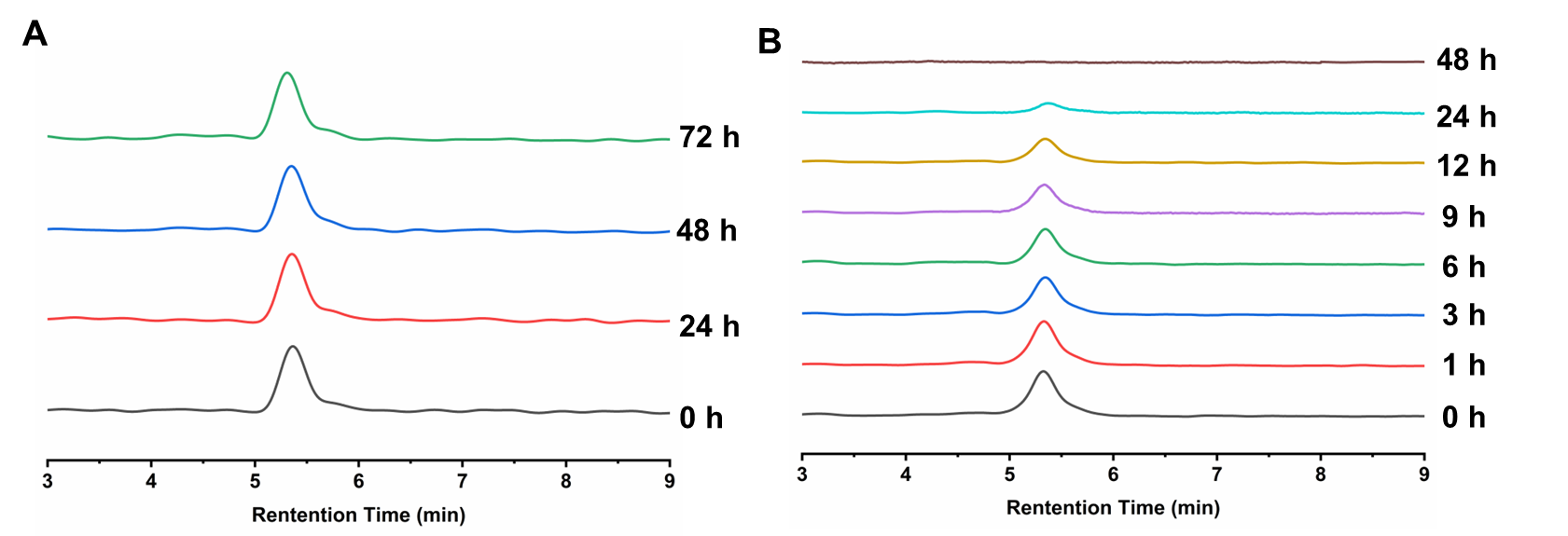
**

**Figure S22. (**A) Stability of HRP in PBS/MeOH (9/1, V/V, pH 7.4) tested by RP-HPLC at 37 °C in the dark; (B) Reduction of 50 μM HRP in 2 mM ascorbic acid in PBS/MeOH (9/1, V/V, pH 7.4) solution was tested by RP-HPLC at 37 ºC in the dark.

Stability of Pt^IV^ complexes in cells (Figures S23-S25).

**
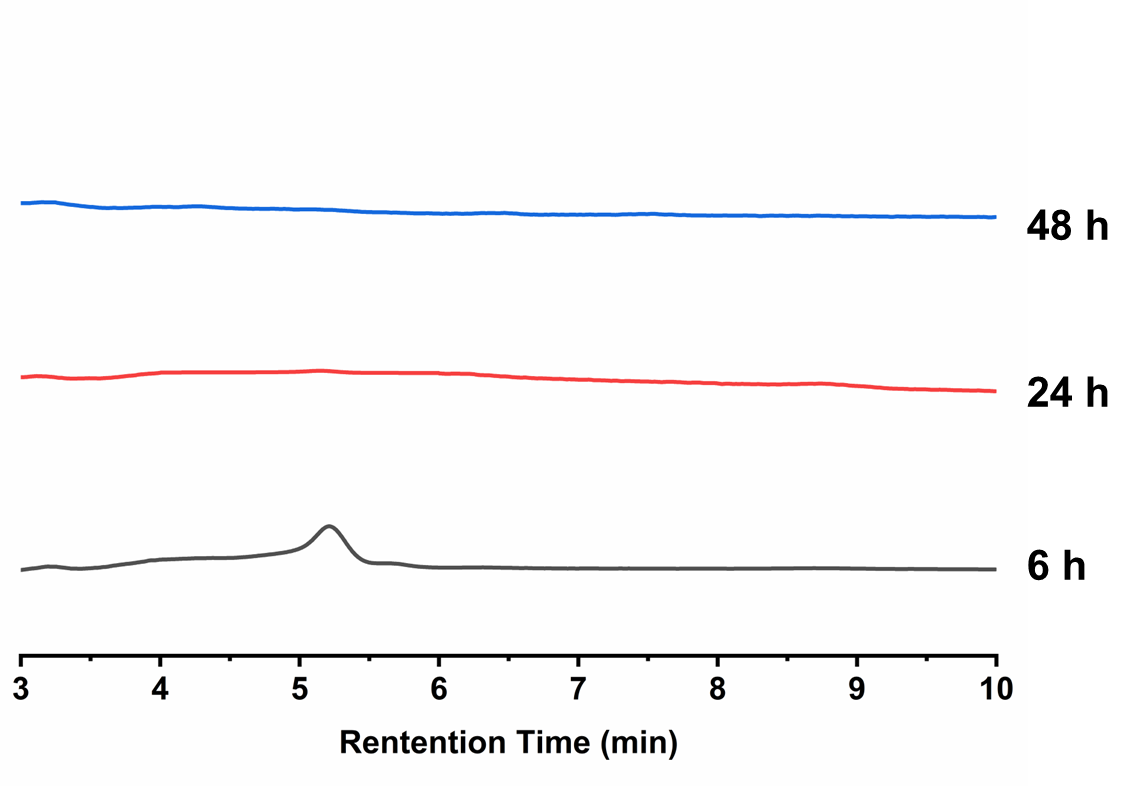
**

**Figure S23.** Stability of MRP after incubation in 4T1 cells at different time tested by RP-HPLC.

**
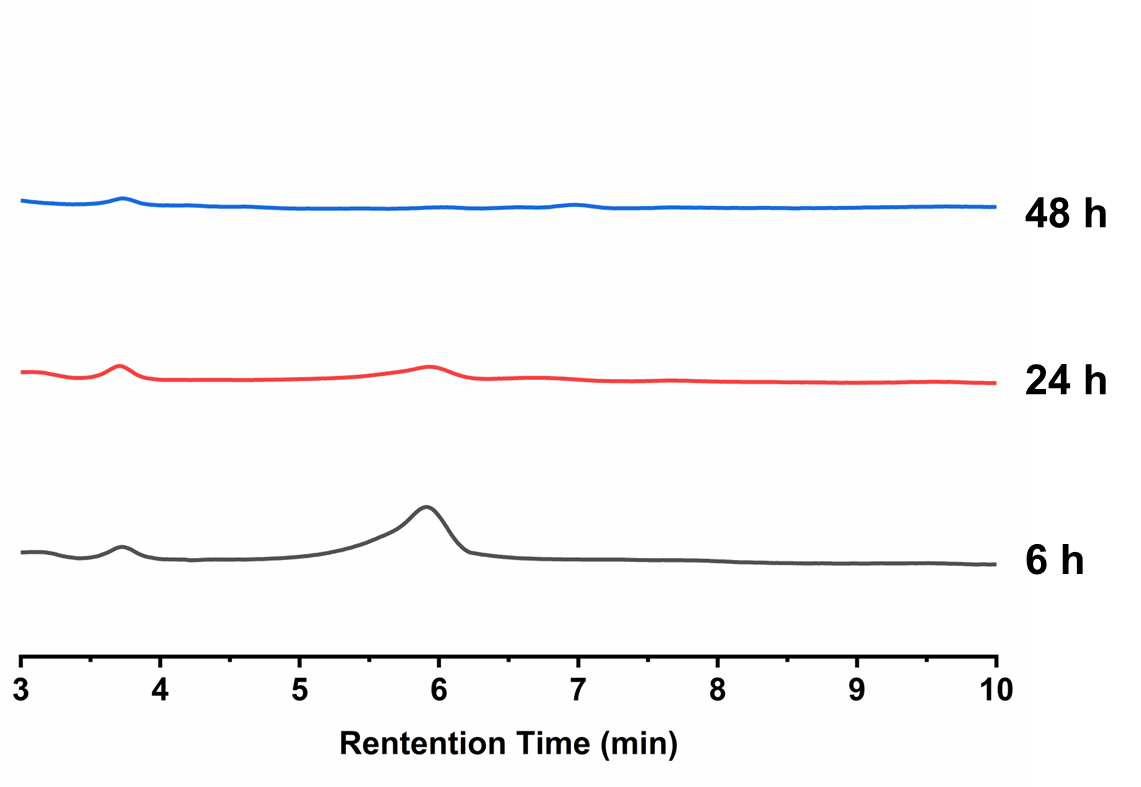
**

**Figure S24.** Stability of DRP after incubation in 4T1 cells at different time tested by RP-HPLC.


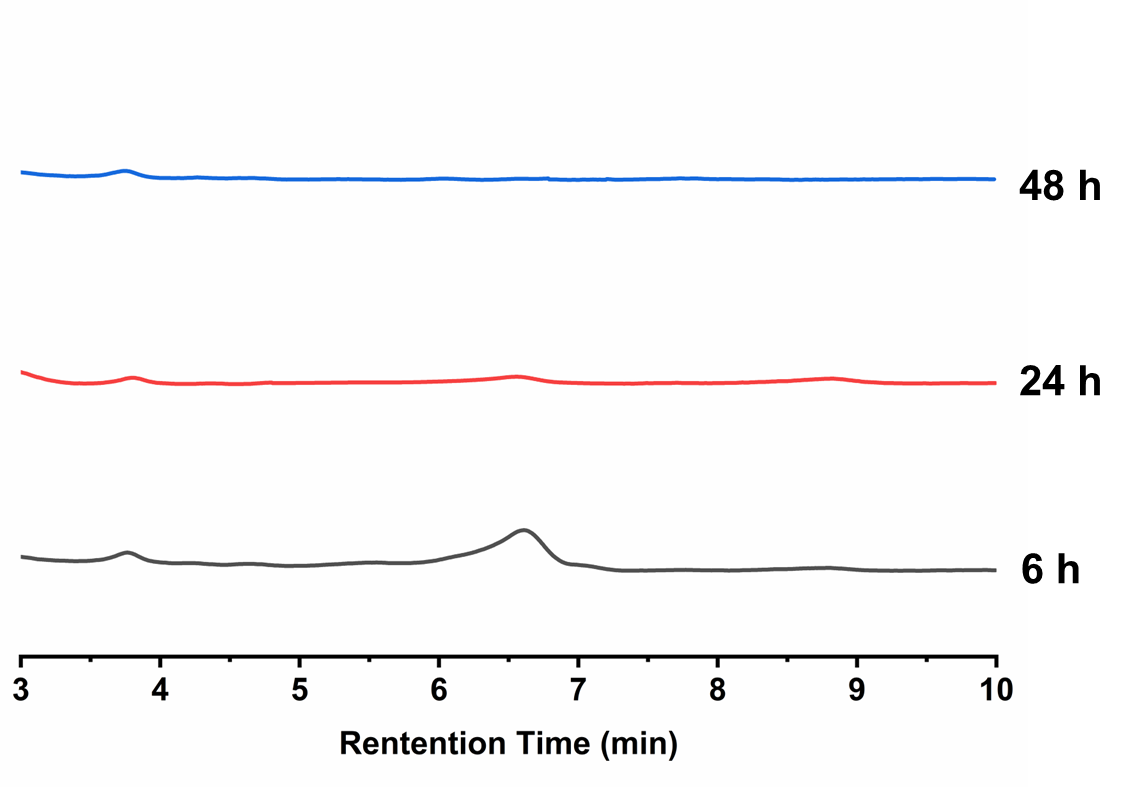


**Figure S25.** Stability of HRP after incubation in 4T1 cells at different time tested by RP-HPLC.

¹⁹⁵Pt NMR spectra of degradation experiments (Figures S26-S28).


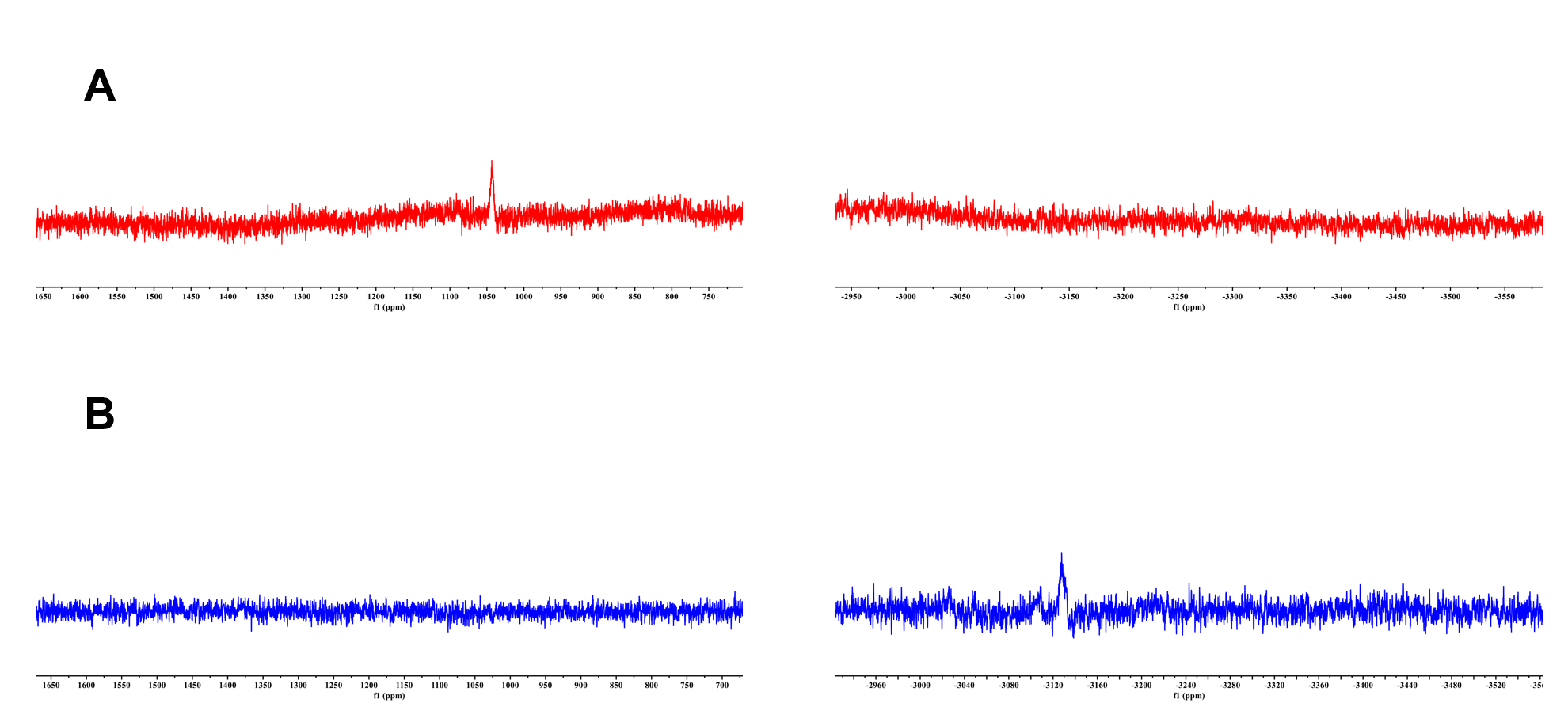


**Figure S26.** ¹⁹⁵Pt NMR spectra of (A) complex MRP and (B) MRP reacted with 40 equivalents of AsA at 37°C for 24 h in 60% DMSO/40% D_2_O. Zooms of the 700 to 1650 and -3550 to -2950 ppm regions are shown.


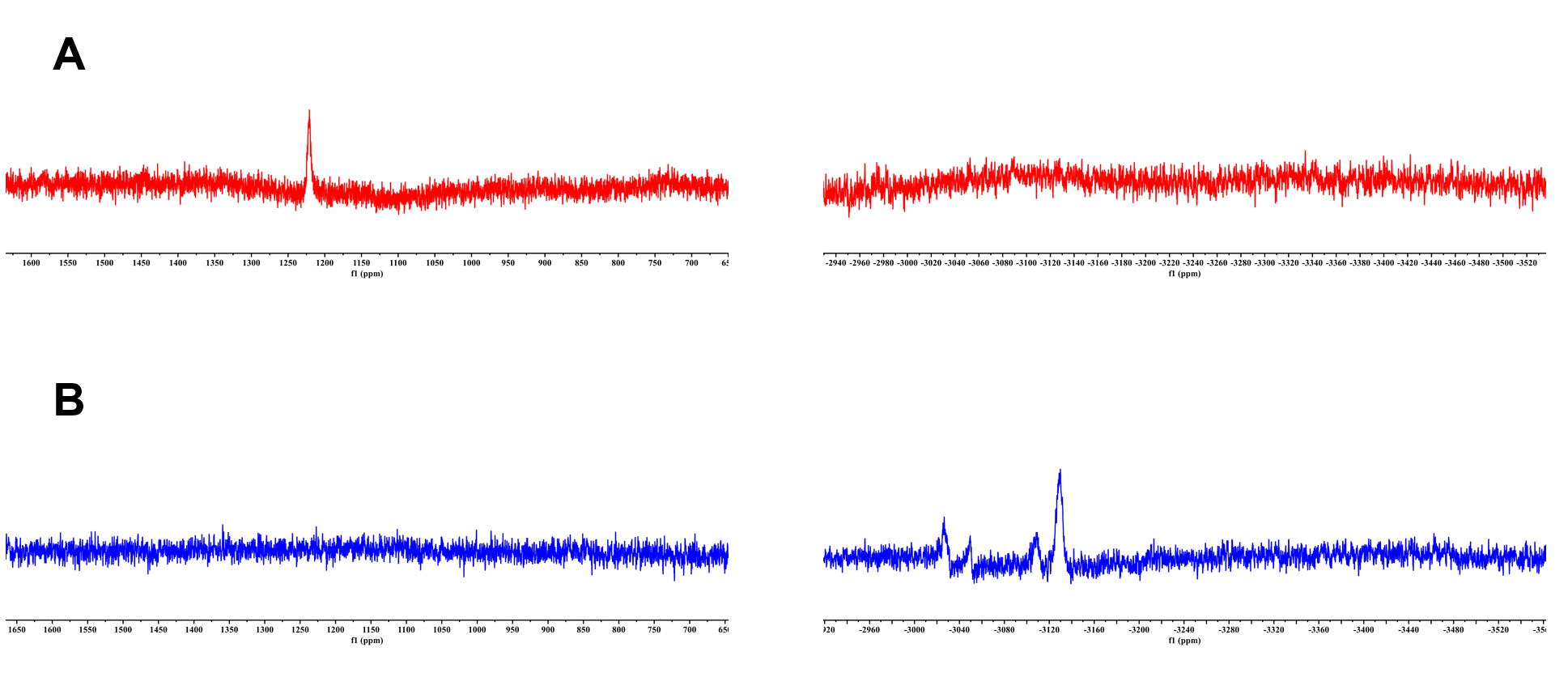


**Figure S27.** ¹⁹⁵Pt NMR spectra of (A) complex DRP and (B) DRP reacted with 40 equivalents of AsA at 37°C for 72 h in 60% DMSO/40% D_2_O. Zooms of the 700 to 1650 and -3550 to -2950 ppm regions are shown.


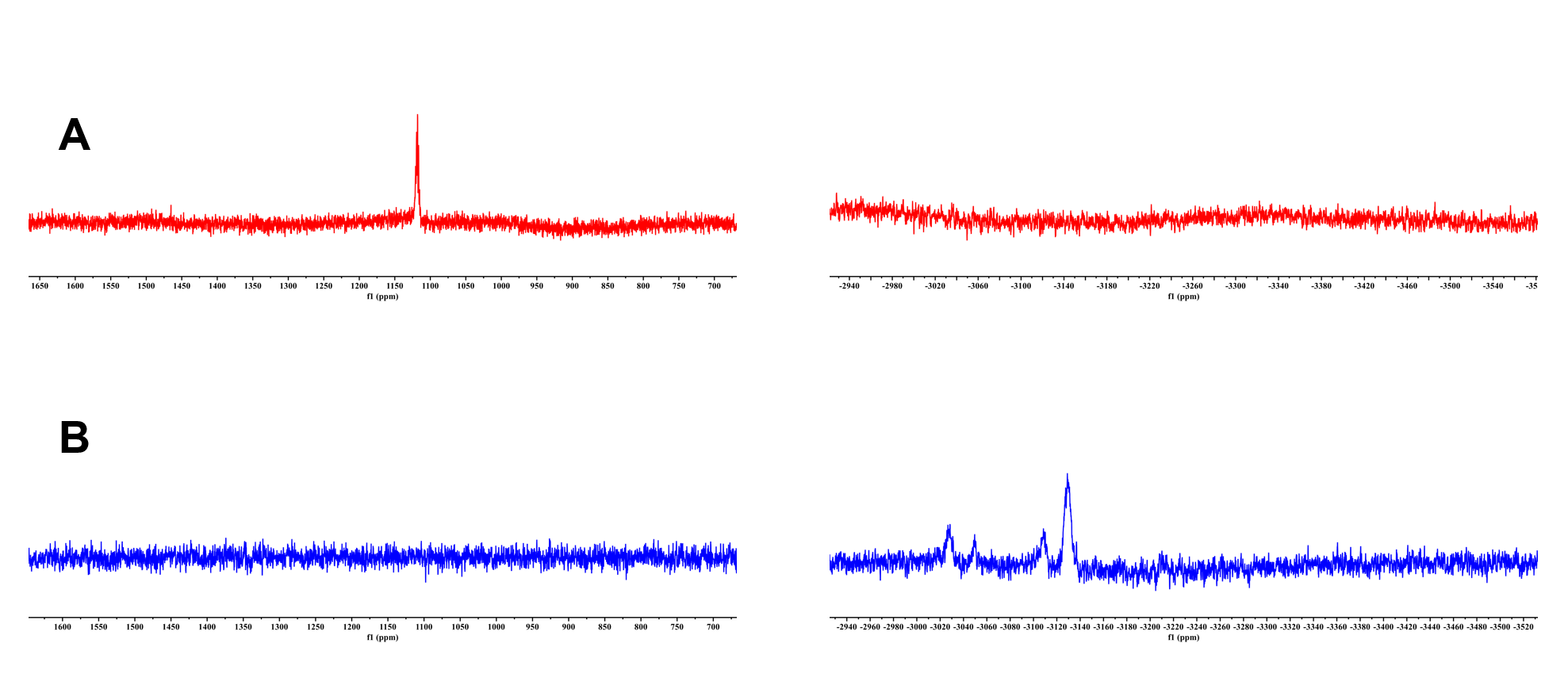


**Figure S28.** ¹⁹⁵Pt NMR spectra of (A) complex HRP and (B) HRP reacted with 40 equivalents of AsA at 37°C for 72 h in 60% DMSO/40% D2O. Zooms of the 700 to 1650 and -3550 to -2950 ppm regions are shown.

Binding assay of MRP, DRP and HRP with 5'-GMP (Figures S29-S31).

**
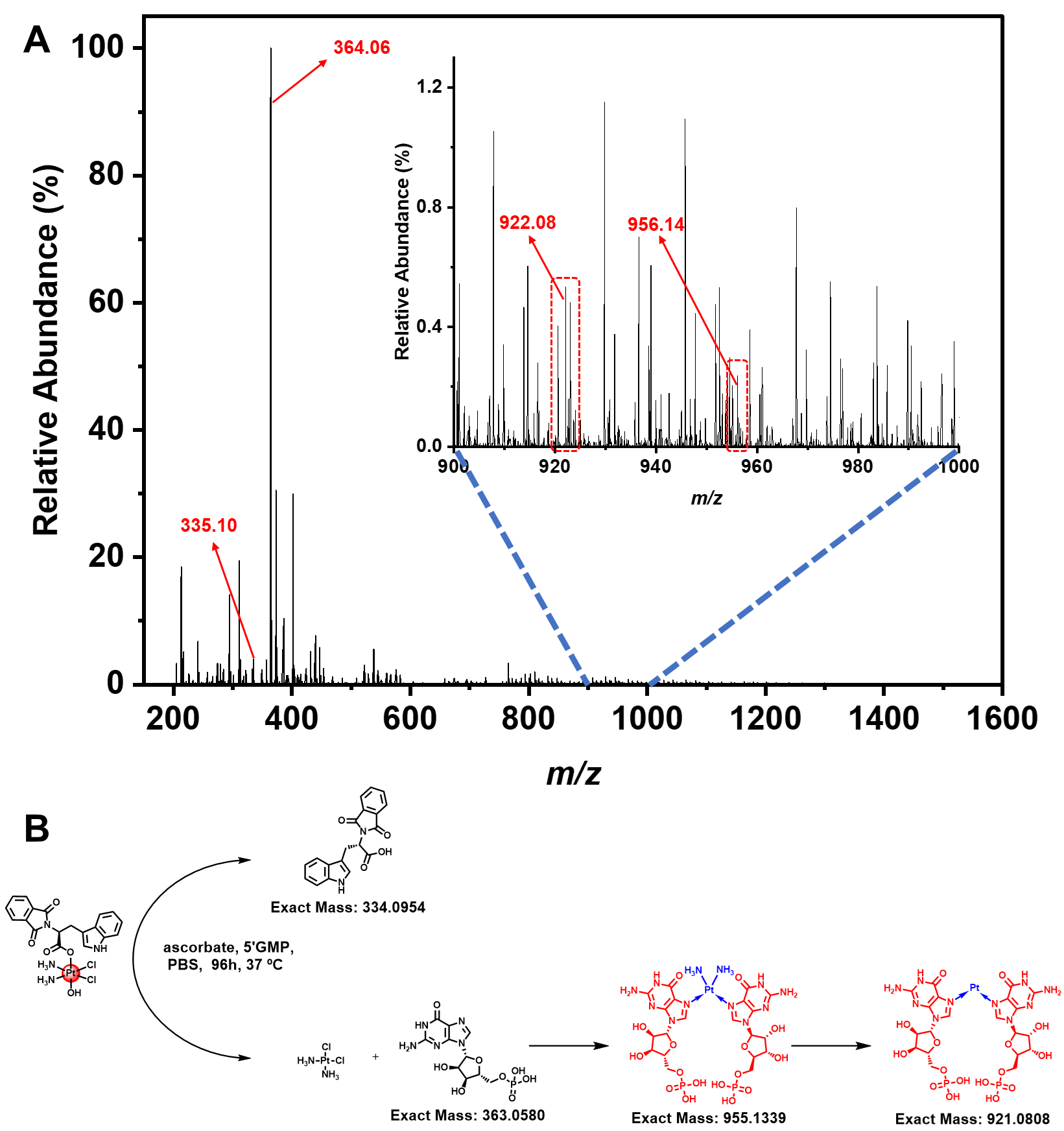
**

**Figure S29. Monitoring the reaction of 5’-GMP with MRP**. (A) Incubate DRP (1 mM), 5'-GMP (4 mM) and ascorbic acid (10 mM) at 37 °C in the dark for 96 h, then measure the mixture by ESI-MS. Several possible peak assignments are marked in red and assume different corresponding chemical structures. B) The probable reaction schematic representation.

**
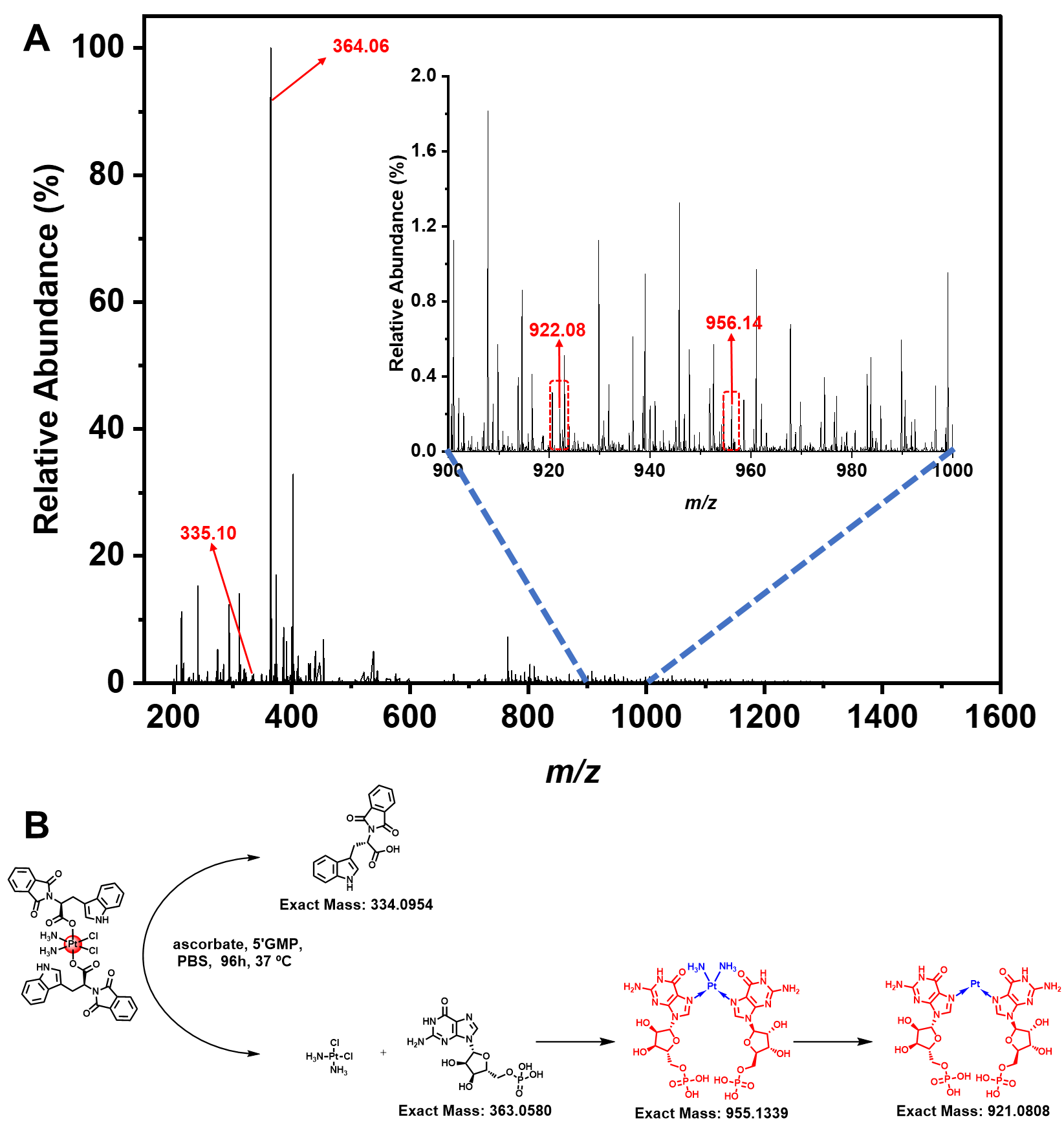
**

**Figure S30.** **Monitoring the reaction of 5’-GMP with DRP**. A) Incubate DRP (1 mM), 5'-GMP (4 mM) and ascorbic acid (10 mM) at 37 °C in the dark for 96 h, then measure the mixture by ESI-MS. Several possible peak assignments are marked in red and assume different corresponding chemical structures. B) The probable reaction schematic representation.

**
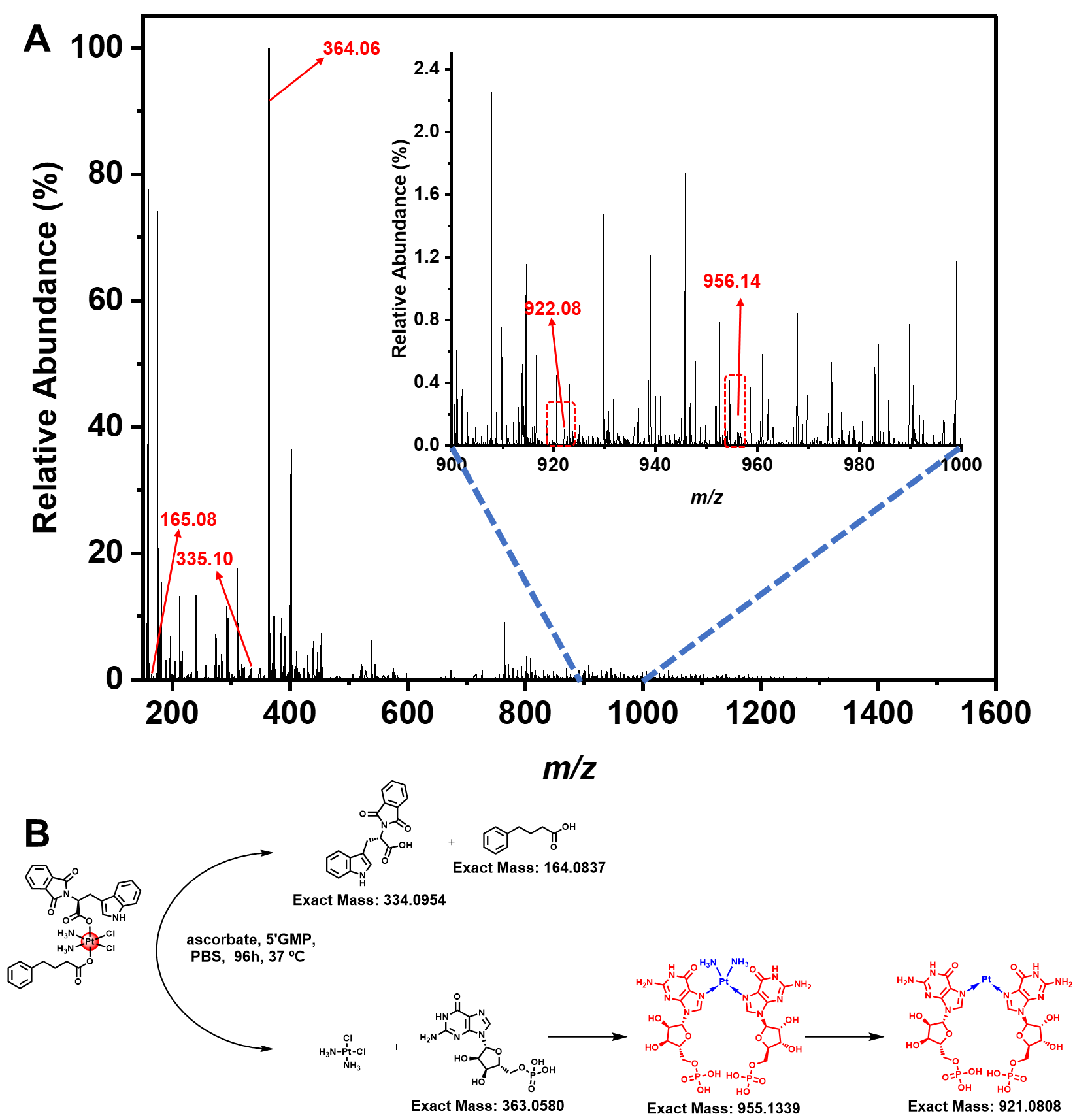
**

**Figure S31.** **Monitoring the reaction of 5’-GMP with HRP**. A) Incubate HRP (1 mM), 5'-GMP (4 mM) and ascorbic acid (10 mM) at 37 °C in the dark for 96 h, then measure the mixture by ESI-MS. Several possible peak assignments are marked in red and assume different corresponding chemical structures. B) The probable reaction schematic representation.

Results of Transcriptomic analysis (Figure S32-39)


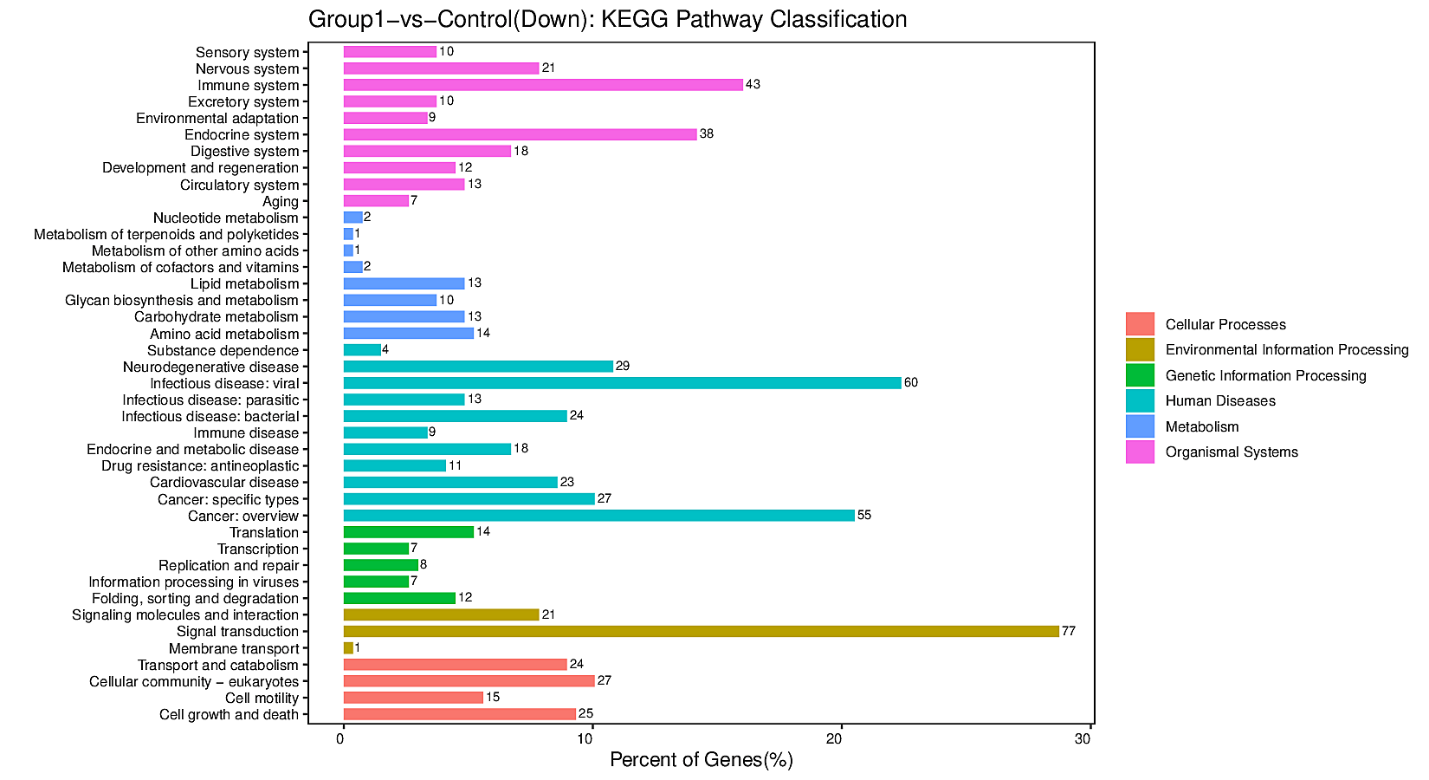


**Figure S32.** CDDP-vs-Saline (Up): KEGG Pathway Classification.

**
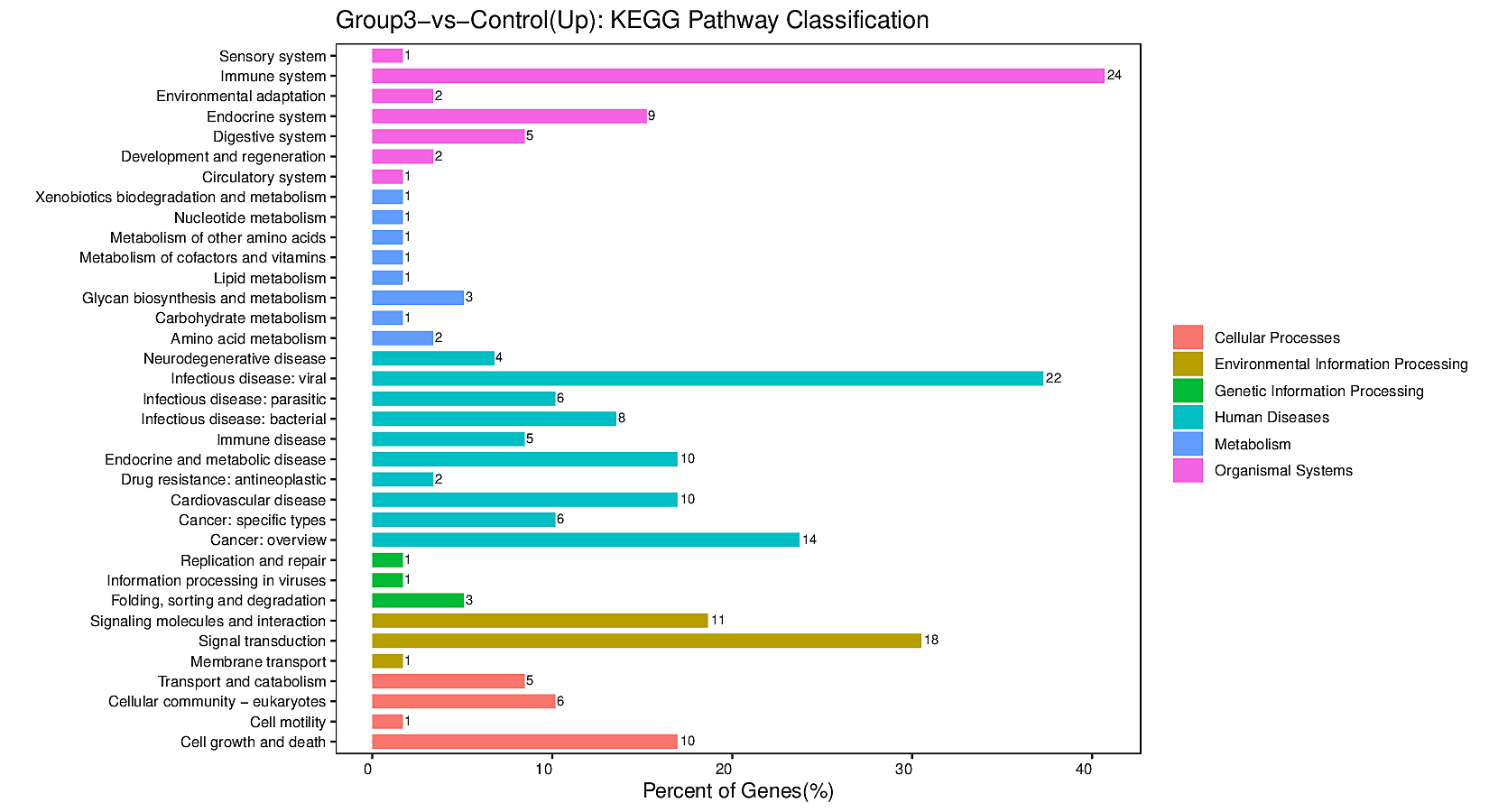
**

**Figure S33.** MRP-vs-Saline (Up): KEGG Pathway Classification.

**
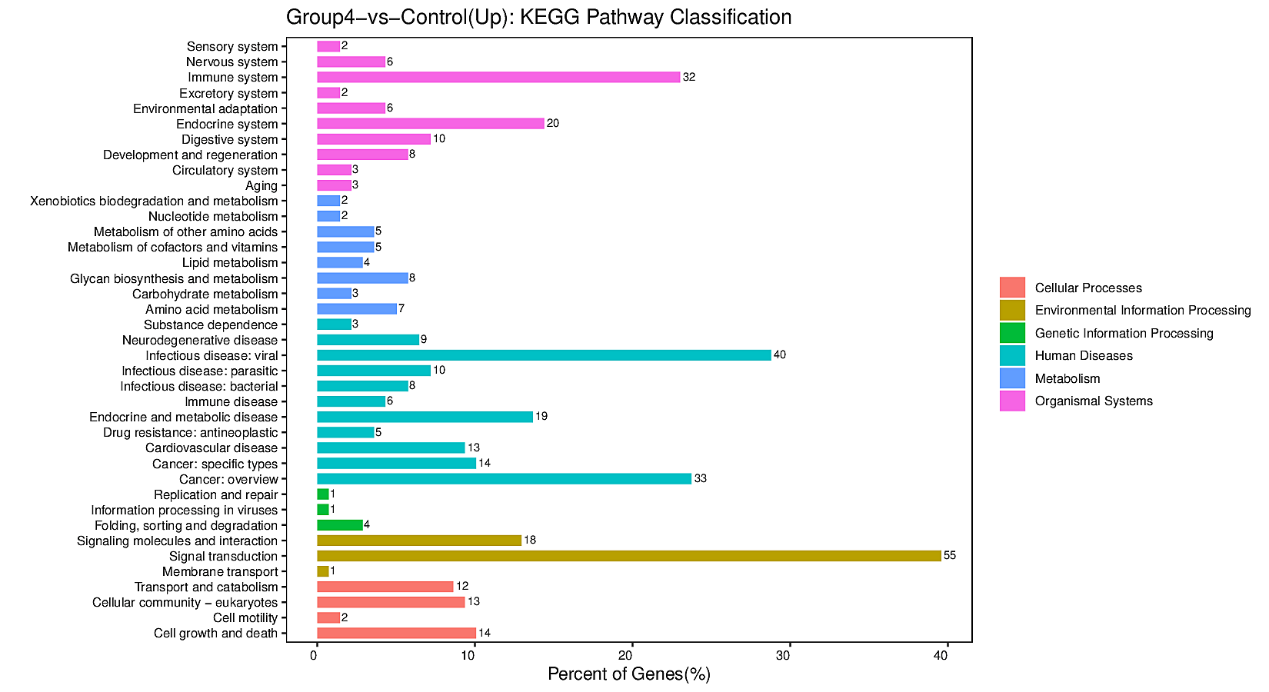
**

**Figure S34.** DRP-vs-Saline (Up): KEGG Pathway Classification.

**
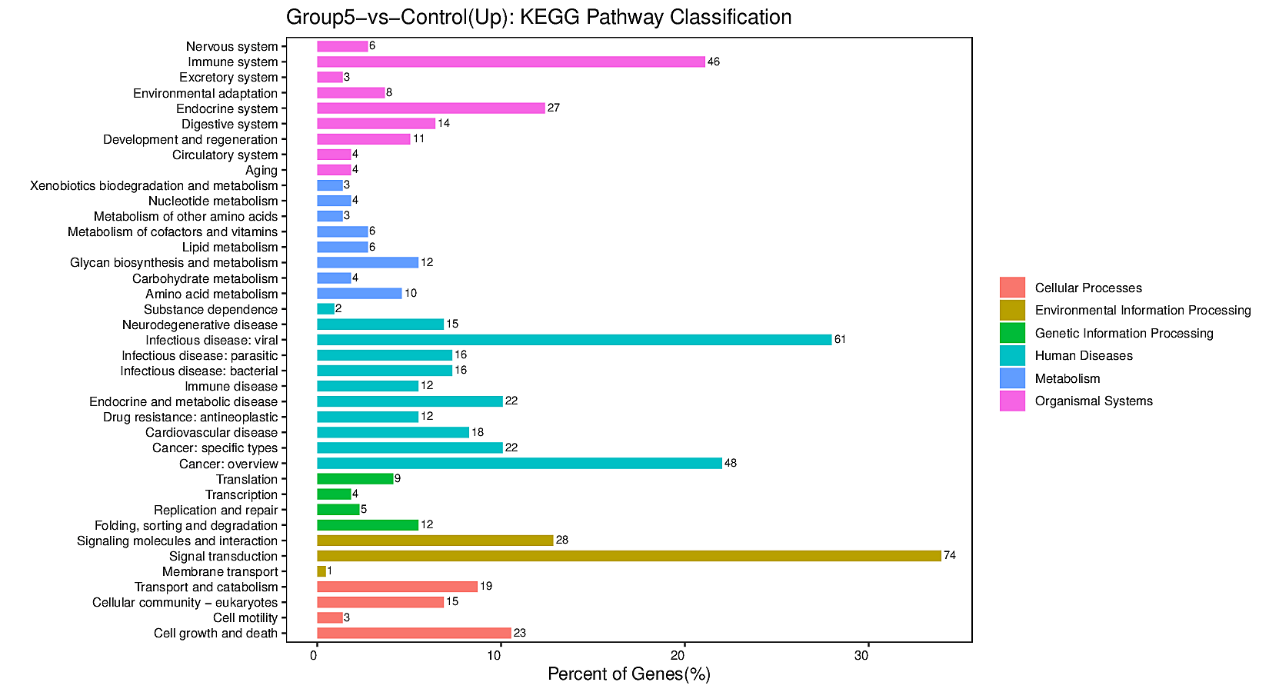
**

**Figure S35.** HRP-vs-Saline (Up): KEGG Pathway Classification.

**
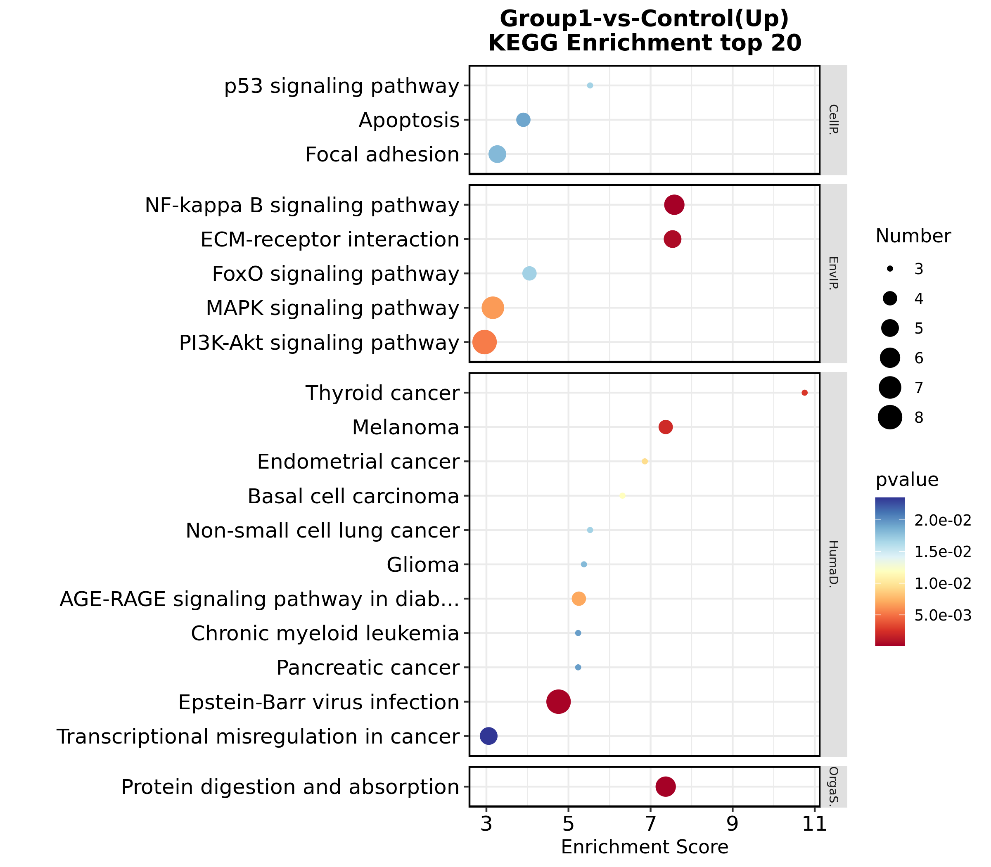
**

**Figure S36.** Group CDDP-vs-Saline (UP) KEGG Enrichment top 20.

**
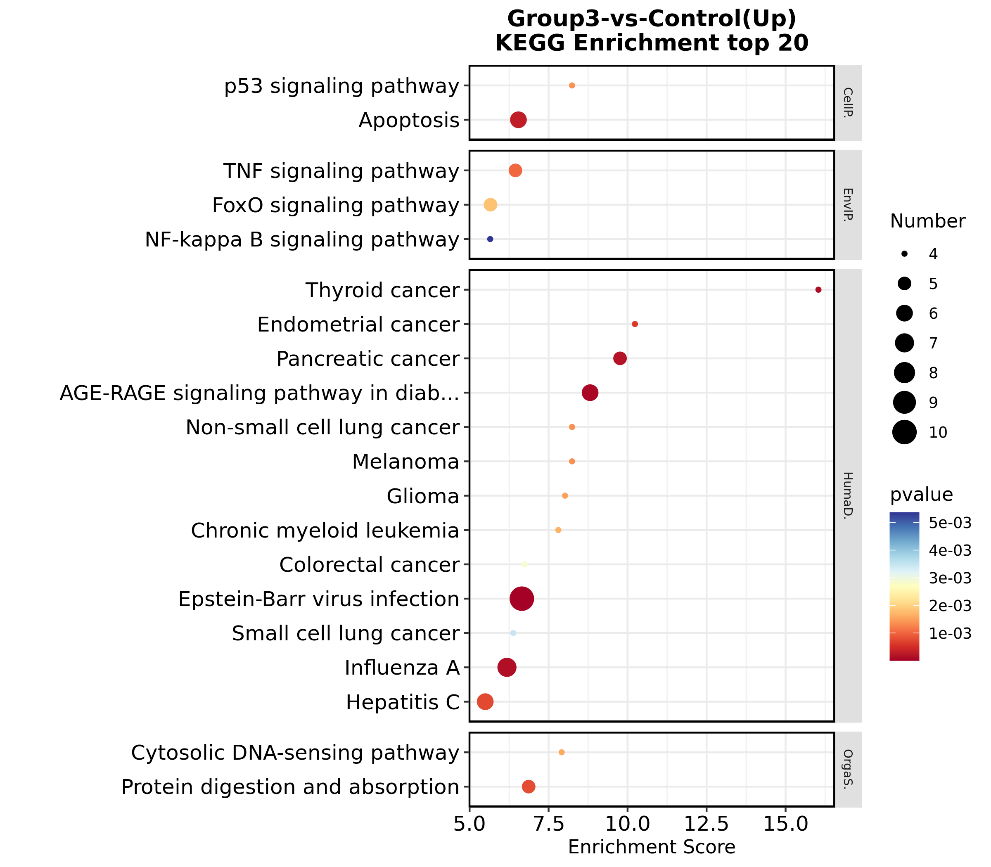
**

**Figure S37.** Group MRP-vs-Saline (UP) KEGG Enrichment top 20.

**
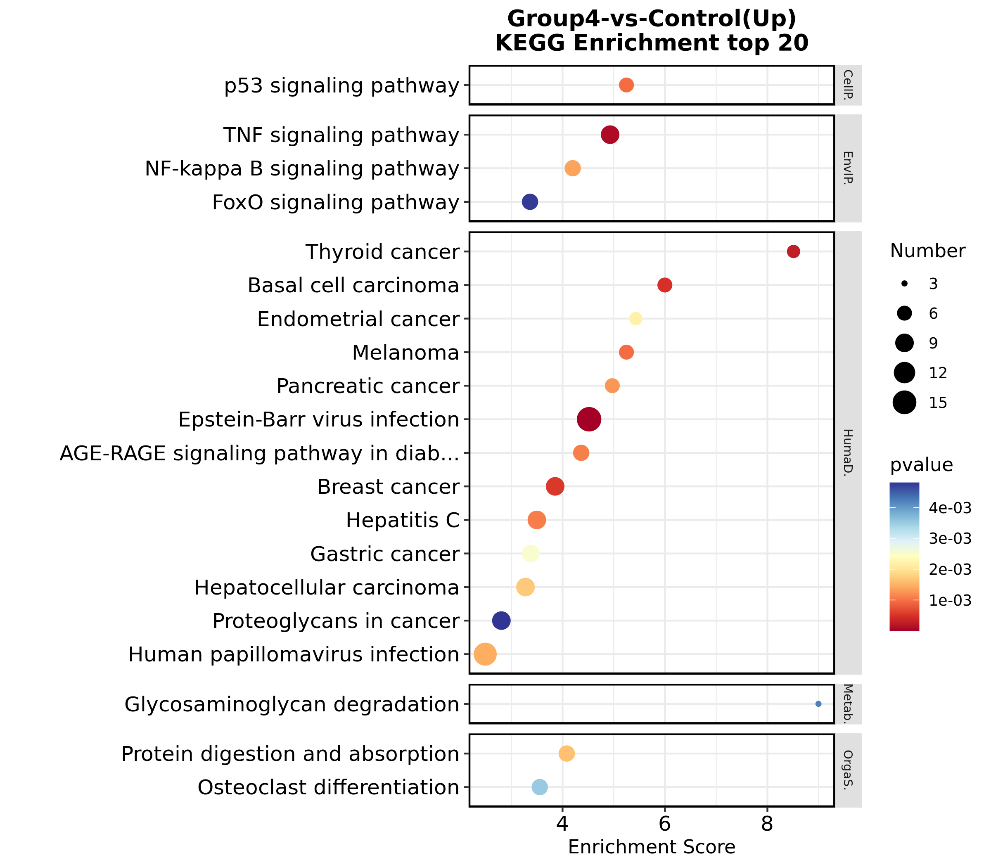
**

**Figure S38.** Group DRP-vs-Saline (UP) KEGG Enrichment top 20.

**
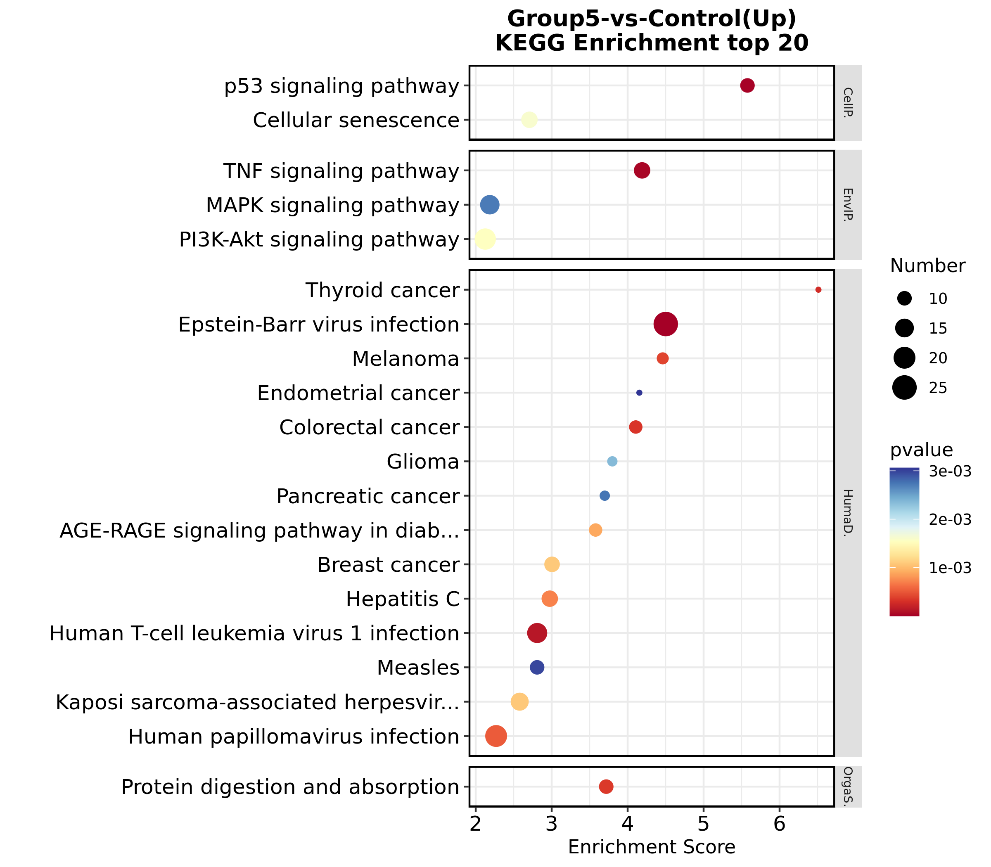
**

**Figure S39.** Group HRP-vs-Saline (UP) KEGG Enrichment top 20.

Statistical count of cells undergoing pyroptosis (Figure S40).

**Figure S40.** Statistical count of cells undergoing pyroptosis, (n = 3). One-way ANOVA with Tukey’s multiple comparison test. Statistical significance is demarked as *P < 0.05, **P < 0.01, and ***P < 0.001, throughout the text. Error bar indicated SEM.

WB results of Caspase-3 induced by CDDP (Figure S41).

**
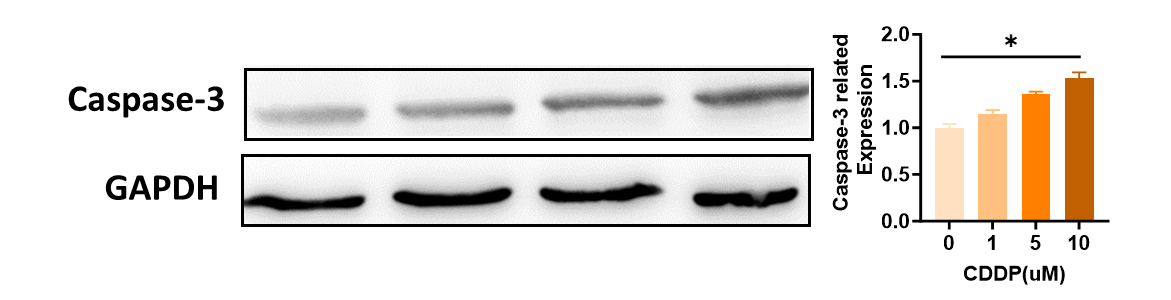
**

**Figure S41.** Expression levels of caspase-3 protein in 4T1 cells after treatment with different concentrations of CDDP and statistical data, (n = 3). Unpaired Student’s t test. Statistical significance is demarked as *P < 0.05, **P < 0.01, and ***P < 0.001, throughout the text. Error bar indicated SEM.

WB results of GSDME induced by RG108 (Figure S42).

**
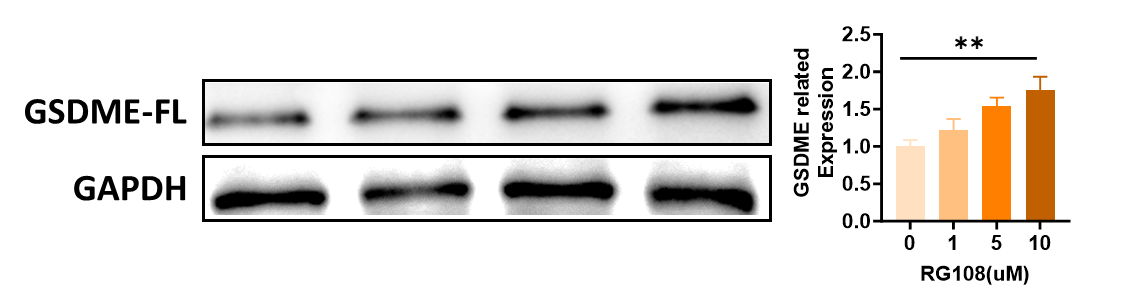
**

**Figure S42.** Expression levels of GSDME protein in 4T1 cells after treatment with different concentrations of RG108 and statistical data, (n = 3). Unpaired Student’s t test. Statistical significance is demarked as *P < 0.05, **P < 0.01, and ***P < 0.001, throughout the text. Error bar indicated SEM.

qPCR statistical analysis of IL-18 and IL-β (Figure S43).

**
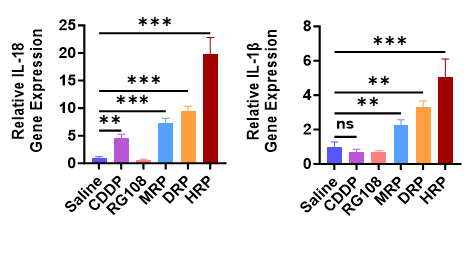
**

**Figure S43.** qPCR statistical analysis of IL-18 and IL-β, (n = 6). One-way ANOVA with Tukey’s multiple comparison test. Statistical significance is demarked as *P < 0.05, **P < 0.01, and ***P < 0.001, throughout the text. Error bar indicated SEM.

Tunnel positive cell counts (Figure S44).

**Figure S44.** Tunnel positive cell counts in representative tumor sections from mice in corresponding treatment groups after 15 days, (n = 6). One-way ANOVA with Tukey’s multiple comparison test. Statistical significance is demarked as *P < 0.05, **P < 0.01, and ***P < 0.001, throughout the text. Error bar indicated SEM.

Immunofluorescence staining of HMGB1 (Figure S45).


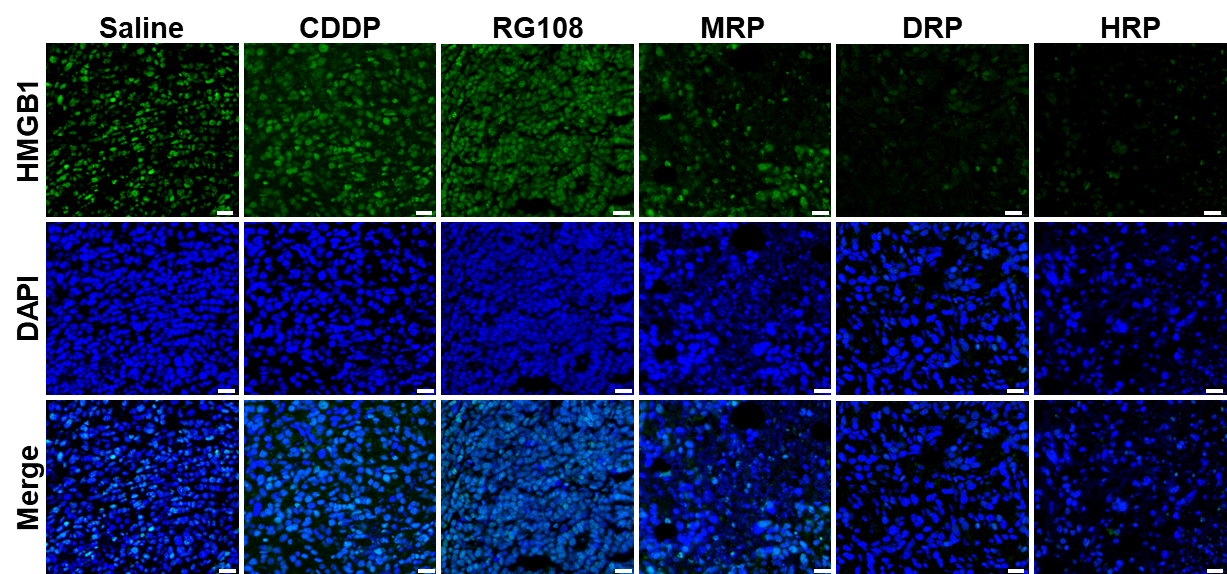


**Figure S45.** Immunofluorescence staining of HMGB1 in tumor tissues after different treatments, Scale bar = 20 μm.

H&E-stained images of heart tissues (Figure S46).

**
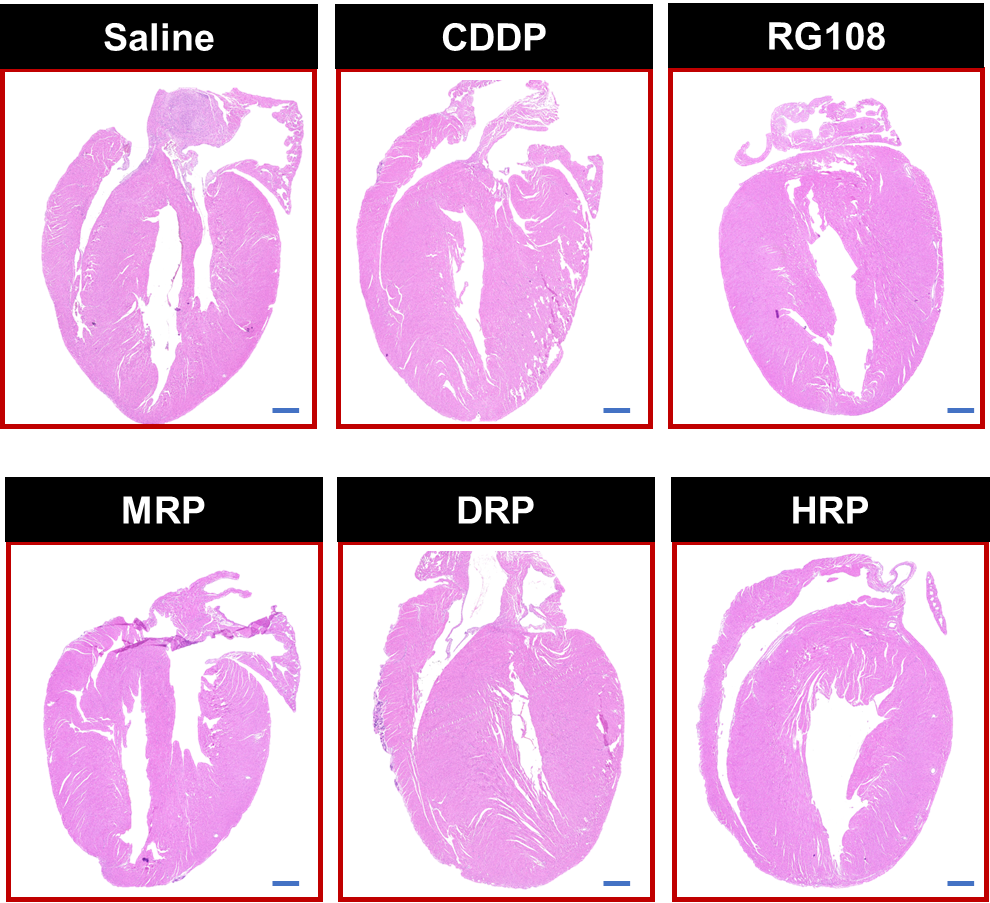
**

**Figure S46.** H&E-stained images of heart tissues after different treatments, bar = 500μm.

H&E-stained images of kidney tissues (Figure S47).

**
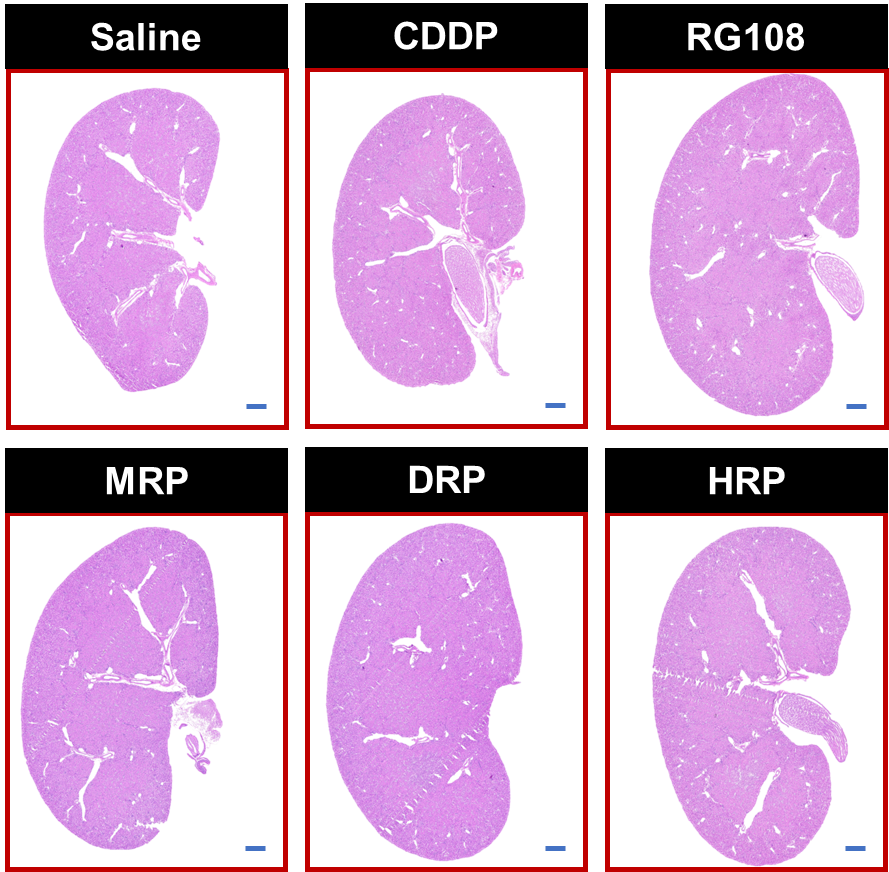
**

**Figure S47.** H&E-stained images of kidney tissues after different treatments, bar = 500μm.

Expression levels of UREA and CREA (Figure S48).

**
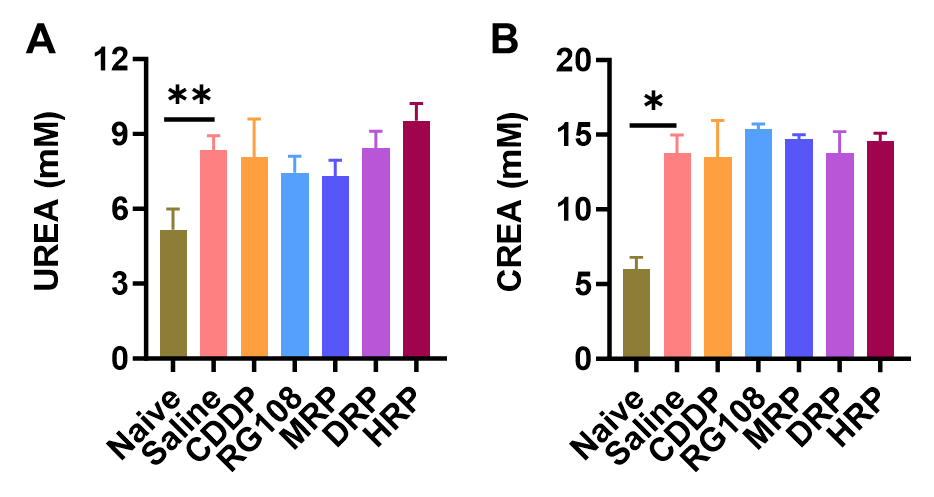
**

**Figure S48.** Expression levels of UREA and CREA in kidney tissues, (n = 6). Unpaired Student’s t test. Statistical significance is demarked as *P < 0.05, **P < 0.01, and ***P < 0.001, throughout the text. Error bar indicated SEM.

The gating strategy for the Flow cytometry analysis (Figures S49-51).


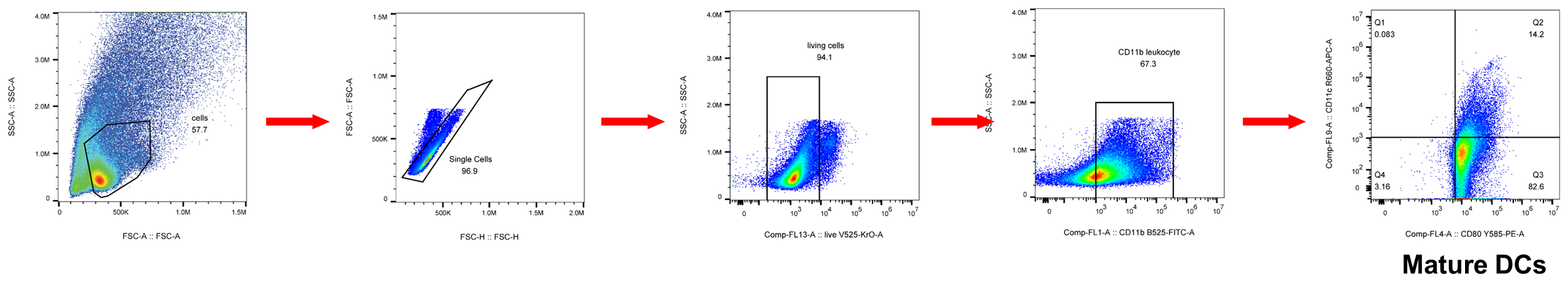


**Figure S49.** The gating strategy for the Flow cytometry analysis of mature DCs.


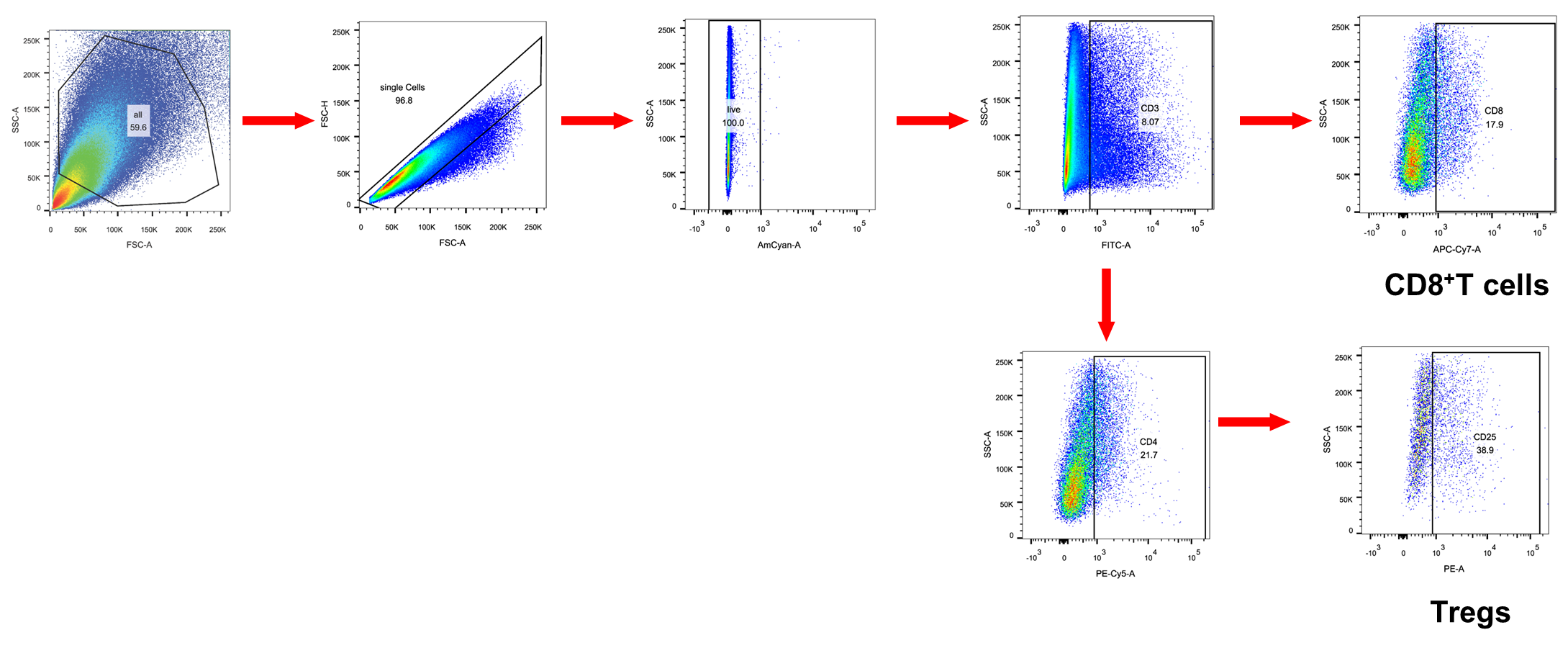


**Figure S50.** The gating strategy for the Flow cytometry analysis of CD8^+^T cells and Tregs.


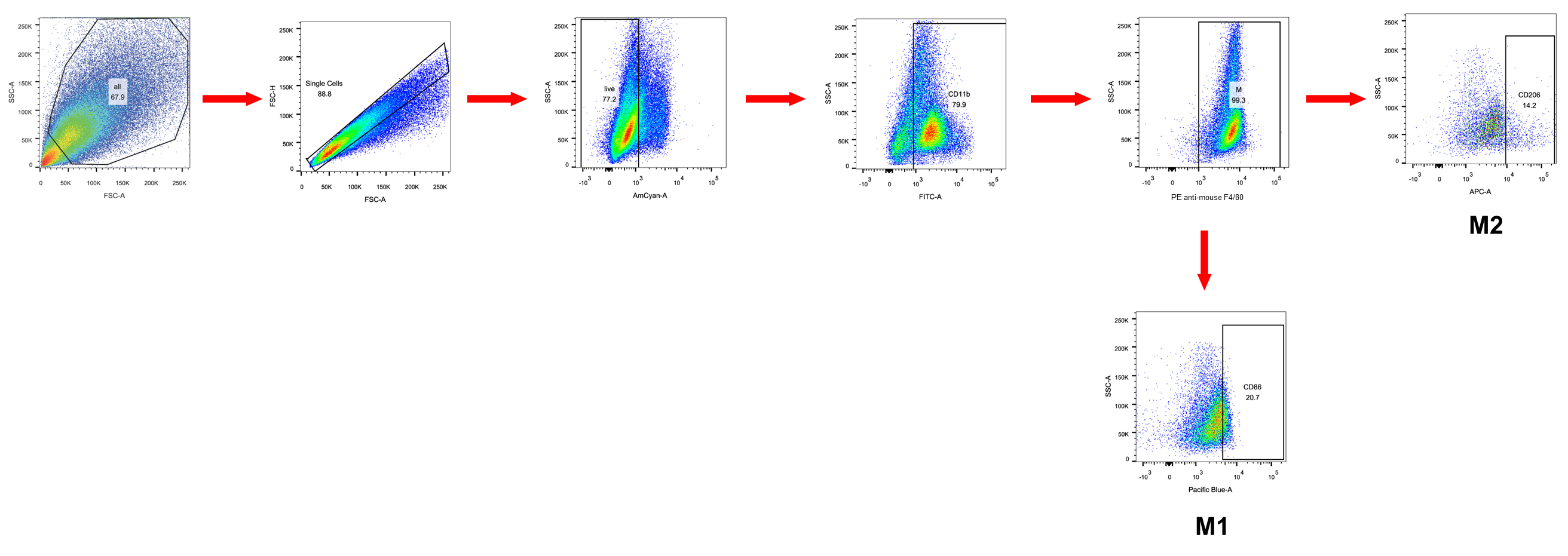


**Figure S51.** The gating strategy for the Flow cytometry analysis of M1 and M2.

.

# Methods

1 Materials

All reagents and solvents were of analytical grade and used as received without further purification. **CDDP** was purchased from Shandong Boyuan Pharmaceutical Co., Ltd., China. 4-Phenylbutyric acid, L-tryptophan, phthalic anhydride, 1-Ethyl-3-(3-dimethylaminopropyl) carbodiimide (EDCI), N-hydroxysuccinimide (NHS), 2-(7-Azabenzotriazol-1-yl)-N, N, N’, N’-tetramethyluronium hexafluorophosphate (HATU), triethylamine (TEA), and hydrogen peroxide (H_2_O_2_, 30%) were purchased from Shanghai Titan Scientific Co., Ltd. and used as received without further purification. Deionized water was purchased from Shanghai Aladdin Biochemical Technology Co., Ltd. 4-Phenylbutanoic anhydride was prepared according to the literature procedure^[1]^.

Annexin V conjugated with fluorescein isothiocyanate (Annexin V-FITC), propidium iodide (PI), Hoechst33342 staining solutions were purchased from KeyGen Biotech Co., Ltd. (Nanjing, China). The Genomic DNA mini preparation kit (G8156-50T-PKG1) was purchased from Adamas Life (Shanghai) Co., Ltd. Pro Caspase-3 (ab32499), GSDME (ab215191), GAPDH (ab9485) antibodies, and goat anti-rabbit HRP (IgG H&L) (ab97051) secondary antibody were purchased from Abcam. Cleaved Caspase-3 (9661) antibody was purchased from Cell Signaling Technology, Inc. IL1-β (E-EL-M0037c) ELISA kit was purchased from Elabscience Biotechnology Co., Ltd., and IL-18 (GEM0-96T) ELISA kit was purchased from Servicebio Biotechnology Co., Ltd. Reverse Transcription Kit PrimeScriptTmRT Master Mix (RRO36A) was purchased from TaKaRa Biotechnology (Dalian) Co., Ltd.

Flow cytometry antibodies, including Brilliant Violet 510™ anti-mouse CD45 (103138), FITC anti-mouse CD3 (100203), PE/Cyanine5 anti-mouse CD4 (100513), APC/Cyanine7 anti-mouse CD8a (100713), FITC anti-mouse/human CD11b (101206), PE-Cyanine7 anti-mouse Ly-6G (127617), PE anti-mouse F4/80 (123109), Brilliant Violet 421™ anti-mouse CD86 (105032), Alexa Fluor® 647 anti-mouse CD206 (141712), PE anti-mouse CD80 (104707), and Zombie Aqua™ Fixable Viability Kit (423102) were purchased from Biolegend Biotechnology Co., Ltd.

2 Cell Lines

The human breast cancer cell lines MDA-MB-231 (ER/PR-negative, HER2-negative, TNBC), BT-474 (ER-positive, HER2-positive, PR negative) and MCF-7 (ER-positive, PR-positive, HER2-negative), the human bladder cancer cell line T24, the human prostate cancer cell DU145, the human non-small cell lung cancer cell A549 and the mouse mammary carcinoma cell line 4T1 were purchased from American Type Culture Collection (ATCC). These cells were cultured in high-glucose DMEM medium containing 10% FBS and RPMI-1640 medium containing 10% FBS, respectively. The cells were incubated at 37°C in a humidified incubator with 5% CO_2_ for subsequent experiments and observations.

3 Animals

Female BALB/c mice (5-6 weeks old) were purchased from Jihui Laboratory Animal Care (Shanghai, China). Mice were housed at a temperature of 20-26°C, relative humidity of 40-60%, and a day/night cycle of 12 h of light and 12 h of darkness while avoiding direct exposure to bright sunlight. All animal studies were approved by the Institutional Animal Care and Use Committee of Tongji University (TJAC00122101).

4 General Methods

^1^H-, ^13^C-,^195^Pt-NMR NMR spectra were obtained on a Bruker 600 MHz NMR spectrometer at 298 K. Electrospray ionization mass spectra (ESI-MS) were obtained using an LCQ spectrometer (Finnigan). Electrospray ionization high-resolution mass spectrometry (ESI-HRMS) spectra were obtained with Thermo Scientific Q Exactive combined quadrupole Orbitrap mass spectrometer. Platinum contents were measured by an inductively coupled plasma optical emission spectrometer (PE Optima 8300 ICP-OES).

5 Synthesis and characterization

5.1 Synthesis of RG108

RG108 was synthesized and optimized with a 93% yield according to a literature procedure^[2]^. The detailed steps are as follows:

Phthalic anhydride (0.74 g, 0.005 mol) and L-tryptophan (1.02 g, 0.01 mol) were refluxed in 300 mL of pyridine under argon for 12 h. The resulting solution was concentrated to 5 mL and added dropwise into ice-cold 10% hydrochloric acid with vigorous stirring to precipitate a solid. The solid was filtered, washed with water (3 × 20 mL), and dried in an oven at 40 °C for several hours, yielding a pure faint yellow powder with a 93% yield.

^1^H NMR (600 MHz, DMSO-*d*_6_) δ 13.33 (s, 1H, H1), 10.75 (s, 1H, H2), 7.81 (s, 4H, H3 - H6), 7.47 (d, *J* = 7.9 Hz, 1H, H7), 7.24 (d, *J* = 8.1 Hz, 1H, H8), 7.02 (d, *J* = 2.4 Hz, 1H, H9), 7.01 - 6.96 (m, 1H, H10), 6.89 (t, *J* = 7.5 Hz, 1H, H11), 5.11 (dd, *J* = 10.2, 5.8 Hz, 1H, H12), 3.60 - 3.52 (m, 2H, H13, H14).^13^C NMR (151 MHz, DMSO-*d*_6_) δ 170.35, 167.20, 136.02, 134.82, 130.88, 126.91, 123.31, 120.96, 118.38, 117.89, 111.43, 109.72, 52.63, 24.08. ESI-HRMS (positive ion mode) m/z: [M + H]^+^ calcd. C_19_H_14_N_2_O_4_ for 335. 1026, found 335.1022, [M + Na]^+^ calcd. C_19_H_14_N_2_O_4_ for 357. 0954, found 357.0846.

5.2 Synthesis of RG108-NHS

To a mixture of RG108 (167 mg, 0.5 mmol) and NHS (69 mg, 0.6 mmol) in CH_2_Cl_2_ (20 mL), EDCI (115 mg, 0.6 mmol) was added. The reaction mixture was stirred overnight at room temperature. The resulting solution was washed with water (3 × 20 mL) and saturated saline (20 mL), then dried with anhydrous sodium sulfate. The product was further purified by silica gel column chromatography using CH_2_Cl_2_ as the eluent, yielding a faint yellow powder. Yield: 200.5 mg (93%).

^1^H NMR (600 MHz, CDCl_3_) δ 8.09 (s, 1H, H1), 7.76 (dd, *J* = 5.4, 3.1 Hz, 2H, H2, H3), 7.65 (dt, *J* = 6.0, 3.1 Hz, 2H, H4, H5), 7.59 (d, *J* = 8.0 Hz, 1H, H6), 7.27 (dd, *J* = 2.4, 1.0 Hz, 1H, H7), 7.15 - 7.11 (m, 1H, H8), 7.08 - 7.02 (m, 2H, H9, H10), 5.62 (dd, *J* = 10.7, 5.3 Hz, 1H, H11), 3.92 (dd, *J* = 15.2, 10.7 Hz, 1H, H12), 3.79 (dd, *J* = 15.2, 5.3 Hz, 1H, H13), 2.81 (s, 4H, H14 - H17). ^13^C NMR (151 MHz, CDCl_3_) δ 168.62, 166.94, 165.15, 136.18, 134.35, 131.57, 127.08, 123.76, 123.19, 122.34, 119.81, 118.50, 111.31, 109.81, 50.86, 25.68, 25.04. ESI-HRMS (positive ion mode) m/z: [M + H]^+^ calcd. for C_23_H_17_N_3_O_6_ 432.1190, found 432.11772, [M + NH_4_]^+^ calcd. for C_23_H_17_N_3_O_6_ 449.1456, found 449.1440.

5.3 Synthesis of Oxoplatin

Oxoplatin was synthesized and optimized with a 94% yield following a literature procedure^[3]^. involving the oxidation of cisplatin with 30% hydrogen peroxide (H_2_O_2_). The detailed steps are as follows:

Cisplatin (1 g, 3.45 mmol) was suspended in a mixture of deionized water and 100 mL of 30% H_2_O_2_. An additional 100 mL of 30% H_2_O_2_ was added dropwise over 1 h at 60 °C. The reaction mixture was then stirred at 60 °C overnight. The resulting clear solution was concentrated to 20 mL and crystallized with 200 mL of ethanol, yielding oxoplatin (1.08 g, 3.24 mmol, 94%) as yellowish crystals.

5.4 Synthesis of MRP

To a suspension of oxoplatin (50 mg, 0.14 mmol) in DMSO (4 mL), RG108-NHS ester (60.4 mg, 0.14 mmol) was added, and the reaction mixture was stirred for 24 h at 60 °C to form a clear yellow solution. DMSO was removed by excessive addition of tert-butyl ether (50 mL), yielding the crude product. The crude product was dissolved in 1 mL of methanol and then added to 30 mL of tert-butyl ether. This process was repeated several times, and the product was dried under vacuum to yield an off-white solid. Yield: 54 mg (56%).

^1^H NMR (600 MHz, DMSO-d6) δ 10.70 (s, 1H, H1), 7.87 - 7.74 (m, 4H, H2 - H5), 7.46 (d, J = 7.9 Hz, 1H, H6), 7.25 (d, J = 8.0 Hz, 1H, H7), 7.04 - 6.97 (m, 2H, H8, H9), 6.91 (t, J = 7.4 Hz, 1H, H10), 5.94 (q, J = 61.6, 56.9 Hz, 6H, H11 - H16), 5.03 (dd, *J* = 8.9, 6.9 Hz, 1H, H17), 3.58 (d, *J* = 8.5 Hz, 2H, H18, H19). ^13^C NMR (151 MHz, DMSO-*d*_6_) δ 175.86, 167.60, 136.05, 134.56, 131.19, 126.98, 123.04, 122.91, 120.88, 118.23, 117.88, 111.41, 110.77, 54.62, 24.90. ^195^Pt NMR (129 MHz, DMSO-*d*_6_) δ = 1043.49. ESI-HRMS (positive ion mode) m/z: [M + H]^+^ calcd. for [C_19_H_20_C_l2_N_4_O_5_Pt 650.0532, found 650.0526, [M + Na]^+^ calcd. for C_19_H_20_C_l2_N_4_O_5_Pt 672.0356, found 672.0423.

5.5 Synthesis of DRP

To a mixture of oxoplatin (50 mg, 0.14 mmol) and RG108 (200 mg, 0.56 mmol) in DMSO (5 mL), HATU (212 mg, 0.56 mmol) and TEA (33.4 mg, 0.66 mmol) were added. The reaction mixture was stirred at room temperature for 72 h under an argon atmosphere to obtain a yellow solution. This solution was then added dropwise to an excess of methyl tert-butyl ether (50 mL) to remove the DMSO, yielding the crude product. After being dried under vacuum, the crude product was washed with water (3 × 20 mL) and dried under vacuum again to obtain an off-white solid. Yield: 82 mg (56.6%).

^1^H NMR (600 MHz, DMSO-*d*_6_) δ 10.73 (s, 2H, H1, H1’), 7.80 (m, *J* = 4.3 Hz, 8H, H2 – H5, H2’ – H5’), 7.48 (d, *J* = 7.9 Hz, 2H, H6, H6’), 7.25 (d, *J* = 8.1 Hz, 2H, H7, H7’), 7.11 - 6.96 (m, 4H, H8, H9, H8’, H9’), 6.92 (t, *J* = 7.5 Hz, 2H, H10, H10’), 6.71 - 6.34 (m, 6H, H11 - H16 ), 5.18 (dt, *J* = 10.7, 5.4 Hz, 2H, H17, H17’), 3.69 - 3.54 (m, 4H, H18, H19, H18’ , H19’). ^13^C NMR (151 MHz, DMSO-*d*_6_) δ 175.88, 167.36, 136.04, 134.58, 131.04, 126.87, 123.07, 123.01, 120.89, 118.23, 117.85, 111.38, 110.31, 53.74, 24.85. ^195^Pt NMR (129 MHz, DMSO-*d*_6_) δ = 1218.02. ESI-HRMS (positive ion mode) m/z: [M + H]^+^ calcd. for C_38_H_32_C_l2_N_6_O_8_Pt 650.0532, found 650.0526.

5.6 Synthesis of the HRP

MRP (64.9 mg, 0.1 mmol) and 4-phenylbutanoic anhydride (155 mg, 0.5 mmol) were stirred in 5 mL of DMF at 40 °C for 72 h under an argon atmosphere. After the reaction, the solution was added dropwise to an excess of methyl tert-butyl ether (50 mL) to obtain the crude product. The HRP was further purified by silica gel column chromatography using MeOH/CH_2_Cl_2_ (1:10) as the eluent, yielding a faint yellow powder. Yield: 42 mg (52.8%).

^1^H NMR (600 MHz, DMSO-*d*_6_) δ 10.72 (s, 1H, H1), 7.80 (p, *J* = 4.5 Hz, 4H, H2 - H5), 7.48 (d, *J* = 7.9 Hz, 1H, H6), 7.27 (q, *J* = 7.6 Hz, 3H, H7 - H9), 7.19 (dd, *J* = 19.5, 7.3 Hz, 3H, H10 - H12), 7.09 - 6.95 (m, 2H, H13, H14), 6.92 (t, *J* = 7.5 Hz, 1H, H15), 6.53 (q, *J* = 49.6, 43.2 Hz, 6H, H16 - H21), 5.14 (dd, *J* = 9.6, 6.2 Hz, 1H, H22), 3.69 - 3.52 (m, 2H, H23, H24), 2.59 (t, *J* = 7.7 Hz, 2H, H25, H26), 2.25 (t, *J* = 7.5 Hz, 2H, H27, H28), 1.75 (p, *J* = 7.4 Hz, 2H, H29, H30).^13^C NMR (151 MHz, DMSO-*d*_6_) δ 180.50, 175.95, 167.48, 141.99, 136.08, 134.69, 131.09, 128.50, 128.33, 128.25, 126.92, 125.84, 125.72, 123.16, 123.05, 120.98, 118.31, 117.93, 111.48, 110.42, 53.96, 34.81, 34.47, 27.51, 24.88. ^195^Pt NMR (129 MHz, CD_3_OD) δ = 1117.86. ESI-HRMS (positive ion mode) m/z: [M + H]^+^ calcd. for C_29_H_30_C_l2_N_4_O_6_Pt 797.1263, found 797.12609, [M + Na]^+^ calcd. for C_29_H_30_C_l2_N_4_O_6_Pt 819.1082, found 819.11258.

7 Measurement of Partition Coefficient

The partition coefficient of the complexes was measured using the shake-flask method^[4]^. To prepare n-octanol saturated water, equal volumes of n-octanol and water were mixed and shaken on a mechanical shaker for 24 h at room temperature. The compounds were dissolved in n-octanol that was pre-saturated with phosphate-buffered saline (PBS, 10 mM, pH 7.4). Half of the prepared solution was then mixed with an equal volume of water-saturated n-octanol and shaken for 24 h at room temperature. The mixture was centrifuged at 2500 rpm for 15 minutes to separate the phases. The concentration of the solute in the aqueous phase was determined by spectrophotometry (λ_max_ = 254 nm). According to the principle of mass conservation, the drug concentration in the corresponding n-octanol phase and the lipophilic-hydrophilic partition coefficient P_o/w_ (Po/w = Co/Cw =Ao/Aw, where A stands for absorbance) were calculated using the following equation:

8 Stability and Reducibility Degradation of Pt^IV^ Complexes

The stability and reducibility of Pt^IV^ complexes was conducted with reference to a literature procedure^[4-5]^. Prepare stock solutions by dissolving the Pt^IV^ complexes in DMSO. In testing the stability of compounds, 50 μM Pt^IV^ solution were dissolved in PBS/methanol (9:1, V/V, pH 7.4) and incubated in the dark at 37ºC for 24, 48 and 72 h. In testing the Reducibility of compounds, a 50 μM Pt^IV^ solution was mixed with 1 mM ascorbic acid in PBS/methanol (9:1, V/V, pH 7.4) and incubated in the dark at 37ºC for 1, 3, 6, 9, 12, 24, and 48 h. Each sample was analyzed by HPLC using an Agilent ZORRBAX C18 column (4.6 x 150 mm), with an injection volume of 10 μL. The mobile phase consisted of phase A (H_2_O) and phase B (methanol). The absorbance wavelength was set to 254 nm. The stability and reducibility of Pt^IV^ complexes were analyzed using isocratic elution. The elution conditions for MRP were set at 55% phase A and 45% phase B, while the elution conditions for DRP and HRP were set at 35% phase A and 65% phase B. After each elution, 100% phase B was used to thoroughly wash the column to remove any ligands and other molecules generated from the degradation of the final products, preventing interference with the observation of the platinum complexes. The percentages of remaining Pt^IV^ complexes were calculated based on the peak area ratios. The retention times were listed as follows: MRP is 6.696 min, DRP is 4.567 min, and HRP is 5.332 min.

**Intracellular Stability Evaluation of Pt(IV) Prodrugs**

4T1 cells were seeded into culture dishes and allowed to grow to near confluence. MRP, DRP, and HRP were added at a final concentration of 5 μM. MRP was incubated for 6, 12, and 24 h, while DRP and HRP were incubated for 6, 24, and 48 h, respectively. After incubation, the cells were collected by centrifugation, washed three times with PBS, and then lysed with 1 mL methanol under ice-bath conditions followed by ultrasonication for 2 h. After centrifugation, the methanol extracts were collected for analysis. Residual drug content in the methanol extracts was analyzed using reverse-phase high-performance liquid chromatography (RP-HPLC) with isocratic elution. The elution conditions were as follows: 50% H₂O and 50% methanol for MRP, and 40% H₂O and 60% methanol for DRP and HRP.

**Reductive Degradation Analysis of Pt(IV) Prodrugs by ¹⁹⁵Pt NMR Spectroscopy**

MRP, DRP, and HRP were dissolved in a 60% DMSO/40% D₂O mixture at a concentration of 20 mM, followed by the addition of 40 equivalents of ascorbic acid (AsA) as the reducing agent. The solutions were incubated at 37 °C in the dark. MRP was incubated for 24 h, while DRP and HRP were incubated for 72 h. After incubation, ¹⁹⁵Pt nuclear magnetic resonance (NMR) spectroscopy was performed directly to detect platinum signals and evaluate the reductive degradation behavior of the Pt(IV) prodrugs.

1 mM Pt^IV^ complexes were incubated with 5’-GMP (4 mM) in 2 mL PBS (10 mM, pH 5.7) containing 10 mM Ascorbic Acid for 96 h, and then the chelation products were measured by HRMS (ESI-MS).

9 Cell viability assay

4T1 cell lines, MFC cell lines, and EMT-6 cell lines were provided by Sciencelight Biology Science & Technology Co., Ltd. (Shanghai, China). These cells were cultured in high-glucose DMEM medium containing 10% FBS and RPMI-1640 medium containing 10% FBS, respectively. The cells were incubated at 37°C in a humidified incubator with 5% CO_2_ for subsequent experiments and observations. Briefly, the cytotoxicity of different compounds was assessed using the CCK-8 assay. Briefly, cells were seeded in a 96-well plate at a density of 2 × 10³ cells per well in 100 µL of growth medium and were preincubated for 24 h before exposure to the complexes. The stock solution of CDDP was prepared in physiological saline, while those of RG108, MRP, DRP and HRP were prepared in DMSO. The stock solutions were diluted to different concentrations and then added in aliquots of 200 µL per well (DMSO < 0.1%). After 72 h of exposure, the cells were treated with CCK-8 reagent (10 µL) and incubated for 2 h at 37°C. The absorbance of the solution was measured at 450 nm using a Varioskan flash multimode reader (Tecan, Switzerland), which is directly proportional to the number of viable cells.

**10 Cell viability assay of Caspase-3 inhibitor combination**

The cell viability assay for both the Vehicle and Z-DEVD-FMK groups was performed as described above. In addition to the different complexes, 10 μM of Z-DEVD-FMK was further added to the Z-DEVD-FMK group, and the incubation time was 48 h for both groups.

11 In vitro Clonogenic Assay

Harvest cells using trypsin-EDTA and prepare a single-cell suspension, then count and dilute the cells to the desired concentration (e.g., 1000 cells/ml). Seed 100-200 cells per well on a 6-well plate, gently swirl to evenly distribute the cells, and incubate at 37°C with 5% CO₂. Change the medium every 2-3 day without disturbing the cells and continue incubation for 7-14 day until colonies become obvious. Carefully remove the medium, wash with PBS, and fix the cells with 4% paraformaldehyde for 10-15 min at room temperature. Stain the colonies with 0.1% crystal violet for 10-30 min, rinse with water, and let the plates air dry. Count the colonies under a microscope and calculate the colony-forming efficiency using the formula: CFE (%) = (Number f colonies / Number of cells implanted) times 100%.

12 In vitro 2D Cell Migration Assay

The wound healing assay was carried out as previously described. MC38 cells suspension (2.5 × 10^5^ cells/mL) were seeded into the 6-well plate. Used the 200 μL sterile pipette tip to cut out parallel lines when the cells reached nearly 90% confluence. Wash each well carefully to remove the scratched cells using serum-free culture medium. The cells were maintained in serum free culture medium containing the tested compounds or complexes (2 μM) at 37 °C under normoxia for 18 h. At the beginning and end of the experiments, the cross lines were photographed by an inverted microscope.

13 Tumor spheroid 3D migration assay

**Matrigel (HY-K6002, MCE) is thawed on a shaker at 4℃ for 2 h. Matrigel is added onto the cell pellet in an Eppendorf tube and mixed gently avoiding generation of bubbles.1μl of Matrigel is used per drop and these steps are performed on ice. Cells that are mixed with cold Matrigel are gently pipetted into the middle of a well in a 24-well plate into a drop-like shape. For seeding the cells as a drop-like shape, the pipet has to be placed exactly in 90°angle in the middle of the well and the edge of the tip could touch to the plate gently. The cells and Matrigel mixture were pipetted slowly without generating any bubbles in the droplets. The cells and Matrigel mixture should be prepared using extra volumes to account for pipetting errors (for triplicate, a mixture for four drops should be prepared:2×10^7^cells should be mixed with 50μl of Matrigel). The Matrigel drops are solidified in a 37℃ incubator with 5% CO_2_ injection for 3min.The Matrigel drop solidification should not exceed 10minutes to avoid drying out the cells. After the drops are solidified,2ml of cell type specific media is added into each well. The media should be added to the wall of the well to avoid destruction of the 3D Matrigel drop structure. The cells are kept in culture for 72 h. Images of the spheroids were taken at regular intervals to observe cell migration. ImageJ software was used to analyze the images by calculating the migration area and comparing it to the untreated control spheroids. Statistical analysis was performed to assess the significance of differences in cell migration among the various treatment groups.**

After incubating for 72 h, the cells were stained with Calcein-AM (4 × 10^-6^ M) and PI solutions (4 × 10^-6^ M) in PBS buffer solution and incubated for 30 minutes. Finally, the cells were washed three times with PBS buffer solution and observed by Fluorescence microscope to examine their live/dead status.

14 Live/dead cell staining assay

4T1 cells at a density of 2 × 10^5^ cells per well were cultured in a 6-well plate for 12 h to allow cell attachment. The cells were washed twice with PBS, and then compounds CDDP, RG108, MRP, DRP, and HRP were added to the culture medium. After incubating for 48 h, the cells were stained with Calcein-AM (4 × 10^-6^ M) and PI solutions (4 × 10^-6^ M) in PBS buffer solution and incubated for 30 minutes. Finally, the cells were washed three times with PBS buffer solution and observed by Fluorescence microscope to examine their live/dead status.

**15 Live/dead cell staining assay of Caspase-3 inhibitor combination**

The Live/dead cell staining assay for both the Vehicle and Z-DEVD-FMK groups was performed as described above. In addition to the different complexes, 10 μM of Z-DEVD-FMK was further added to the Z-DEVD-FMK group.

16 Intracellular Accumulation

4T1 cells were seeded in a 75 cm² culture flask at a density of 2 × 10^7^ cells per flask. After incubation for 24 h, the cells were treated with 1 μM and 2 μM of the complex at 37 °C for 24 h. The medium was removed and the attached cells were washed twice with cold PBS. The cell pellet was suspended in 500 μL of PBS to obtain a homogeneous cell suspension. The suspension was divided into two parts: one part was used to analyze the platinum content in the whole cells, and the other part was used to analyze the protein content in the cells using the Bicinchoninic Acid Assay (BCA) method. The cell pellets were collected by centrifugation and then digested with nitric acid (200 µL) at 95 °C for 2 h, followed by the addition of H_2_O_2_ (100 µL) and HCl (100 µL) to obtain a fully homogenized solution. The solution was diluted with water to an appropriate volume, and the platinum content was determined by ICP-MS.

17 DNA-binding Assay

4T1 cells were seeded in a 75 cm² culture flask at a density of 2 × 10^7^ cells per flask. After incubation for 24 h, the cells were treated with 1 μM and 2 μM of the complex at 37 °C for 24 h. The medium was removed and the attached cells were washed twice with cold PBS, harvested by trypsinization (0.5 mL), and washed with PBS (1 mL). Cell pellets were lysed in DNAzol reagent (1 mL, Genomic DNA Mini Preparation Kit, Tiangen Biotech, Beijing, Co., LTD). Genomic DNA was extracted from the lysate using pure ethanol (0.5 mL) by incubating the sample at room temperature for 1-3 minutes. The DNA concentration was determined using a Nanodrop 1000 spectrophotometer at 260 nm. The platinum content bound to the DNA was quantified by ICP-MS.

18 mRNA sequencing experimental

18.1 RNA Isolation and Library Preparation

Total RNA was extracted using the TRIzol reagent (Invitrogen, CA, USA) according to the manufacturer’s protocol. RNA purity and quantification were evaluated using the NanoDrop 2000 spectrophotometer (Thermo Scientific, USA). RNA integrity was assessed using the Agilent 2100 Bioanalyzer (Agilent Technologies, Santa Clara, CA, USA). Then the libraries were constructed using VAHTS Universal V6 RNA-seq Library Prep Kit according to the manufacturer’s instructions. The transcriptome sequencing and analysis were conducted by OE Biotech Co., Ltd. (Shanghai, China).

18.2 RNA Sequencing and Differentially Expressed Genes Analysis

The libraries were sequenced on a llumina Novaseq 6000 platform and 150 bp paired-end reads were generated. About 53 raw reads for each sample were generated. Raw reads of fastq format were firstly processed using fastp and the low quality reads were removed to obtain the clean reads. Then about 50 clean reads for each sample were retained for subsequent analyses. The clean reads were mapped to the reference genome using HISAT2. FPKM of each gene was calculated and the read counts of each gene were obtained by HTSeq-count. PCA analysis were performed using R (v 3.2.0) to evaluate the biological duplication of samples.

Differential expression analysis was performed using the DESeq2. Q value < 0.05 was set as the threshold for significantly differential expression gene (DEGs). Hierarchical cluster analysis of DEGs was performed using R (v 3.2.0) to demonstrate the expression pattern of genes in different groups and samples. The radar map of top 30 genes was drew to show the expression of up-regulated or down-regulated DEGs using R packet ggradar. Based on the hypergeometric distribution, GO, KEGG pathway, Reactome and WikiPathways enrichment analysis of DEGs were performed to screen the significant enriched term using R (v 3.2.0), respectively. R (v 3.2.0) was used to draw the column diagram, the chord diagram and bubble diagram of the significant enrichment term.

Gene Set Enrichment Analysis (GSEA) was performed using GSEA software. The analysis was used a predefined gene set, and the genes were ranked according to the degree of differential expression in the two types of samples. Then it is tested whether the predefined gene set was enriched at the top or bottom of the ranking list.

19 Western blot assays

Western blotting was conducted to determine the expression levels of pro-caspase-3, cleaved caspase-3, GSDME, and GSDME-N in 4T1 cells and tumor tissues. 4T1 cells at a density of 2 × 10^5^ cells per well were cultured in a 6-well plate for 12 h to allow cell attachment. The cells were washed twice with PBS, and then compounds CDDP, RG108, MRP, DRP, and HRP were added to the culture medium. After incubating for 48 h, the cells were harvested, lysed, and the total protein was extracted. Similarly, tumor tissues were collected from the treated mice, homogenized, and lysed to extract proteins. Equal amounts of protein (40 μg per lane) were loaded onto an 10% SDS-PAGE gel for separation. Subsequently, the proteins were transferred onto PVDF membranes.

The membranes were blocked for 1 h with TBS containing 5% milk and 0.1% Tween-20 to prevent nonspecific binding. Following the blocking step, the membranes were incubated for 1 h with primary antibodies specific for pro-caspase-3, cleaved caspase-3, GSDME, and GSDME-N, each diluted appropriately (usually 1:1000). After washing with TBS containing 0.1% Tween-20, the membranes were incubated with HRP-conjugated secondary antibodies (diluted 1:1000) for another h. The membranes were then washed again, and the protein bands were visualized using chemiluminescent detection reagents and an imaging system (BIO-RAD, USA). To ensure equal loading of protein, GAPDH was used as loading controls.

The grayscale values of Western blot bands were quantified using ImageJ software. Firstly, the integrated optical density (IOD) of both the target protein bands and internal reference protein bands (e.g., β-actin or GAPDH) was measured, and their ratios were calculated to determine the relative expression levels of the target protein. Subsequently, one experimental group (e.g., the control group) was selected as the reference for normalization to minimize experimental variability and enable cross-comparison between different batches of data.

20 qPCR Experiment

4T1 cells were seeded in a 6-well plate at a density of 2 × 10⁵ cells per well and incubated at 37°C with 5% CO₂ for 12 h to allow cell attachment. Subsequently, the cells were treated with PBS, CDDP (5 μM), RG108 (5 μM), MRP (5 μM), DRP (5 μM) and HRP (5 μM) for 48 h. After incubation, RNA was extracted from 4T1 cells reverse transcribed into cDNA using a reverse transcription kit. Specific primers for the target gene and an appropriate reference gene (β-actin) were designed, the primer sequences are listed in Table S3. The reaction mixture (20 µL) included 10 µL of 2x SYBR Green PCR Master Mix, 0.5 µM forward and reverse primers, and 1 µL of cDNA template. The PCR amplification program included an initial denaturation at 95°C for 3 minutes, followed by 40 cycles of denaturation at 95°C for 15 seconds, annealing at 60°C for 30 seconds, and extension at 72°C for 30 seconds. A melting curve analysis was performed at the end of the amplification. Relative gene expression was calculated using the 2^-ΔΔCt^ method, and normalized to the expression of the reference gene.

21 IL-18 and IL-1β detection

4T1 cells (2 × 10⁵ per well) were seeded in a 6-well plate and incubated for 12 h for attachment. Cells were washed with PBS and treated with 5 µM **CDDP**, RG108, **MRP**, **DRP** or **HRP** for 48 h. After incubation, the supernatant was collected by centrifugation, and IL-18 and IL-1β levels were measured using an ELISA kit according to the manufacturer’s instructions.

22 Flow cytometry assay of immune cells population

In vivo experiments were conducted by administering compounds CDDP, RG108, MRP, DRP, and HRP injection to BALB/c female mice bearing 4T1 tumors (n = 6 mice per group). The treatments were administered three times, with a 3-d interval between each injection. 24 h after the final injection, tumor tissues, and lymph node tissues were harvested for flow cytometry analysis. The tissues were digested and processed into single-cell suspensions using 70 μm cell strainers. Red blood cells were lysed with 5 mL red blood cell lysis buffer, and the remaining cells were washed and resuspended in 200 μL PBS. To block non-specific binding, 10 μL of 1% BSA was added to the suspensions. The cells were then stained with a cocktail of fluorescence-conjugated antibodies, including CD45, CD3, CD4, CD8A, CD25, CD11b, CD86, CD206, CD11c, CD80 and LY-6G, for 30 minutes. Following staining, the cells were fixed and permeabilized using a fixation/permeabilization buffer. Immune cells were further stained with anti-CD16/32 to block Fc receptors and LIVE/DEAD Fixable Violet Dead Cell Staining Kit to distinguish live/dead cells. The stained cells were then analyzed using a flow cytometer (BD, Melody). The resulting data were processed and analyzed using FlowJo software. The gating strategy for the Flow cytometry analysis of immune cells were shown in Figures. S49-S51.

23 In Vivo Antitumor Study

The animal experiments were carried out in compliance with the Regional Ethics Committee for Animal Experiments, and the care and use regulations were approved by the Institutional Animal Care and Use Committee of Tongji University (TJAC00122101).

4T1 tumor model was established via subcutaneous injecting 1 × 10^6^ cells into the hind leg of female BALB/c mice (5 - 6 weeks old). When the volumes of tumor reached 80-150 mm^3^, all the mice were randomly divided into 6 groups of 6 animals each. Mice were injected through angular vein with CDDP, RG108, MRP, DRP, and HRP (CDDP, MRP, DRP, and HRP: 1.5 mgPt/kg body weight, RG108: 2.57 mg/kg body weight). As for the control, the mice were injected with the same buffer. Tumor growth was monitored by measuring the perpendicular diameters of the tumors with a vernier caliper every three days. The injections were administered every three days for a total of 5 injections. Tumor volumes were calculated using the formula: Tumor volume (mm³) = 0.5 × width² × length. The body weights of the mice were also recorded every three days. The mice were sacrificed after the final treatment, and the tumors were excised and weighed. The tumors were then fixed in 4% paraformaldehyde for subsequent histological examination, which included hematoxylin and eosin (H&E) staining, HMGB1 staining and TUNEL staining. H&E staining of the metastatic tumor tissue sections was performed to confirm homology with 4T1 cells. In each group, the number of metastatic foci (pigmented foci at the surface of the organ) was counted, and pictures were taken of the organs containing metastases. The metastatic area percentage was calculated using ImageJ software according to the following formula: metastatic area percentage = pigmented area/total surface area. The number of metastatic and micrometastatic foci in the bulk tissue of a metastasis-containing organ was calculated with a low-power lens.

24 TNF-α, IL-6, IL-12 and IFN-γ detection

Cytokines secreted by cells or tumor tissues were measured using an ELISA assay. Cells and tumor tissue were isolated from mice that received single treatments in different groups, then homogenized and lysed with RIPA lysis buffer. After centrifugation, the supernatant was collected, and the levels of IL-18, IL-1β, TNF-α, IL-6, IL-12, and IFN-γ were analyzed using an ELISA kit, following the manufacturer’s protocol.

25 Statistical Analysis

Relative gene expression was calculated using the 2^-ΔΔCt^ method, and normalized to the expression of the reference gene. Western blot band intensities were quantified using Image J by measuring the IOD ratios of target proteins to internal references (e.g., β-actin/GAPDH), followed by normalization to a control group to minimize variability and enable cross-comparisons. All data were analyzed using GraphPad Prism 10.0. All data were presented as means ± standard error of the mean (SEM). An unpaired Student’s t-test, one-way analysis of variance (ANOVA) or two-way repeated measures ANOVA Bonferroni's multiple comparisons test was performed to determine statistical significance. Statistical significance was indicated as follows: *p < 0.05, **p < 0.01, ***p < 0.001. The sample sizes (n) for each experiment conducted in this study were as follows: colony formation assay (n = 3), 2D migration assay (n = 4-5), 3D tumor spheroid migration assay (n = 7-8), cellular and DNA platinum accumulation in 4T1 cells (n = 3), cell viability assay (n = 3), qPCR analysis (n = 6), Western blot analysis (n = 3-6), ELISA detection of IL-18 and IL-1β release (n = 3-6), comparison of gene expression regulation (n = 3), tumor weight analysis (n = 6), tumor growth curves (n = 6), flow cytometry analysis of immune cell populations (n = 5), metastasis analysis in tissue sections (n = 6), hematological parameter analysis (n = 5-7), body weight monitoring in mice (n = 6), pyroptosis cell quantification (n = 3), and TUNEL assay for cell apoptosis (n = 6).

# Reference

[1] J. Karges, T. Yempala, M. Tharaud, D. Gibson, G. Gasser, A multi‐action and multi‐target Ru^II^-Pt^IV^ conjugate combining cancer‐activated chemotherapy and photodynamic therapy to overcome drug resistant cancers, *Angew. Chem., Int. Ed.* **2020**, *59*, 7069.

[2] D. Chen, K. H. L. Po, P. Blasco, S. Chen, X. Li, Convergent synthesis of calcium-dependent antibiotic CDA3a and analogues with improved antibacterial activity via late-stage serine ligation, *Org. Lett.* **2020**, *22*, 4749.

[3] K. Fang, Y. Sun, J. Yang, X. Hu, M. Chen, R. Li, X. Yang, T. Fan, J. Wu, X. Tong, A dual stimuli-responsive nanoplatform loaded Pt^IV^‐triptolide prodrug for achieving synergistic therapy toward breast cancer, *Adv. Healthcare Mater.* **2023**, *12*, 2301328.

[4] S. Jin, N. Muhammad, Y. Sun, Y. Tan, H. Yuan, D. Song, Z. Guo, X. Wang, Multispecific platinum(IV) complex deters breast cancer via interposing inflammation and immunosuppression as an inhibitor of COX‐2 and PD‐L1, *Angew. Chem., Int. Ed.* **2020**, *132*, 23513.

[5] Q. Cao, D. J. Zhou, Z. Y. Pan, G. G. Yang, H. Zhang, L. N. Ji, Z. W. Mao, CAIXplatins: highly potent platinum(IV) prodrugs selective against carbonic anhydrase IX for the treatment of hypoxic tumors, *Angew. Chem., Int. Ed.* **2020**, *132*, 18715.
